# Convenient Synthesis of Functionalized Unsymmetrical Vinyl Disulfides and Their Inverse Electron-Demand Hetero-Diels-Alder Reaction †

**DOI:** 10.3390/ma14061342

**Published:** 2021-03-10

**Authors:** Bartosz Jędrzejewski, Mateusz Musiejuk, Justyna Doroszuk, Dariusz Witt

**Affiliations:** Department of Organic Chemistry, Faculty of Chemistry, Gdansk University of Technology, Narutowicza 11/12, 80-233 Gdansk, Poland; barjedrz@student.pg.edu.pl (B.J.); mateusz.musiejuk@interia.eu (M.M.); juswiecz@student.pg.gda.pl (J.D.)

**Keywords:** alkenes, cycloaddition, hetero-Diels-Alder, thiosulfonates, vinyl disulfides

## Abstract

The simple, convenient, and efficient methods for the preparation of unsymmetrical vinyl disulfides with additional functional groups under mild conditions with moderate to high yields were designed. The developed methods include the reaction of *S*-vinyl phosphorodithioate with thiotosylates or *S*-vinyl thiotosylate with thiols. The designed methods allow for the synthesis of unsymmetrical vinyl disulfides with additional functionalities such as hydroxy, carboxy, protected amino, or ester groups. Vinyl disulfides reacted with the generated transient *o*-iminothioquinones in an inverse electron-demand [4+2] cycloaddition to produce benzo[*b*][1,4]thiazine derivatives.

## 1. Introduction

The disulfide bond is one of the most important structural functionalities which plays a crucial role affecting the stability, folding, and biological function of proteins and peptides. It also allows the maintenance of the cellular redox balance in cells. Although aforementioned biological properties are significant in life science, disulfides [1,2,3] are also important and versatile compounds due to their applications in material and food chemistry.

The unsymmetrical disulfides can be applied in the formation of self-assembled monolayers (SAMs) on gold or other metals [4,5,6]. Good quality SAMs can be produced both from thiols and disulfides [5]. However, the disulfides provide several practical advantages. They are more stable and significantly more resistant to oxidation. Moreover, in the case of disulfides, the problems associated with intra or intermolecular reactivity of the thiol group can be avoided [7]. The unsymmetrical disulfides give monolayers of well-defined surface compositions without phase separation [8]. When a mixture of two different thiols is used, in some cases, the elimination of cooperative effects associated with the co-adsorption of corresponding thiols cannot be avoided [9]. The surface composition modified by the unsymmetrical disulfides has been applied for double-stranded DNA–protein microarrays [10], DNA immobilization via intercalation [11], and studies on surface reactions on nanoparticles [9]. Unsymmetrical disulfides have been involved in the preparation of the electrostatic self-assembly of nanostructured materials [12,13] and chemosensors for biological applications [3].

Moreover, the synthesis of unsymmetrical disulfides is an important step for the preparation of a variety of compounds involved in medicinal chemistry and advanced organic synthesis [14,15,16,17]. The developments in disulfide bond synthesis have been reviewed recently [18,19,20,21,22]. Although disulfides are very important in numerous fields, effective methods for the preparation of unsymmetrical disulfides are still rare. The most common synthesis of disulfide functionality is based on the nucleophilic substitution reaction of a sulfenyl derivative with a thiol or thiol derivative. The most frequently utilized electrophilic sulfenyl derivatives are: sulfenyl chlorides [23,24], *S*-alkylsulfanylisothioureas [25,26], *S*-alkyl thiosulfates and *S*-aryl thiosulfates (Bunte salts) [27], benzotriazolyl sulfanes [28,29], benzothiazol-2-yl disulfides [30], (alkylsulfanyl)dialkylsulfonium salts [31,32], dithioperoxyesters [33], 2-pyridyl disulfides and derivatives [34,35], sulfonamides [36], *N*-alkyltetrazolyl disulfides [37], sulfenyl thiocyanates [38], sulfenyldimesylamines [39], thiolsulfinates [40] and thiosulfonates [41,42,43], 4-nitroarenesulfenanilides [44], thionitrites [45], thioimides [46], sulfenyl sulfanylsulfinamidines [47,48,49], and thiophosphonium salts [50]. The disulfides can also be efficiently obtained by the reaction of a thiol with a sulfinylbenzimidazole [51], a disulfide exchange reaction promoted by rhodium catalyst [52,53], an electrochemical method [54], using tetrathiomolybdate in the presence of a symmetrical disulfide to promote a ring opening of an aziridine [55,56], or the application of diethyl azodicarboxylate (DEAD) [57] or a solid support [58] to promote a sequential coupling of two different thiols. The oxidation of a mixture of two different thiols to obtain an unsymmetrical disulfide has also been reported recently. The reactions can be accomplished by using 2,3-dichloro-5,6-dicyanobenzoquinone (DDQ) [59,60,61] or iridium (III) photoredox catalysis [62].

The 5,5-dimethyl-2-thioxo-1,3,2-dioxaphosphorinane-2-disulfanyl derivatives are readily available and can be applied for the synthesis of unsymmetrical disulfides with additional functional groups. The synthetic methodology based on the electrophilic disulfanyl derivatives allow one to obtain alkyl-aryl disulfides [63], dialkyl disulfides [64], “bioresistant” disulfides [65], unsymmetrical disulfides of L-cysteine and L-cystine [66], and diaryl disulfides [67]. The electrophilic properties of disulfanyl derivatives of phosphorodithioic acid can also be applied for the synthesis of α-sulfenylated carbonyl compounds [68], phosphorothioates with additional functional groups [69], unsymmetrical alkynyl sulfides [70,71], and symmetrical [72,73] and unsymmetrical trisulfides [74,75].

Block and co-workers isolated ajoene as an E/Z isomers mixture in 1984 [76]. Ajoene was produced as a rearrangement product of allicin from freshly crushed garlic. The structure was established as an allyl sulfoxide containing a vinyl disulfide functionality. The presence of an unusual vinyl disulfide functionality was unexpected and other natural products with such functionality are rare. The activity of Z-ajoene as an anti-thrombotic agent [77] is higher than its *E*-isomer. Due to the higher biological activity of the *Z*-isomer, anticancer studies have focused primarily on this isomer [78,79].

**Scheme 1:**
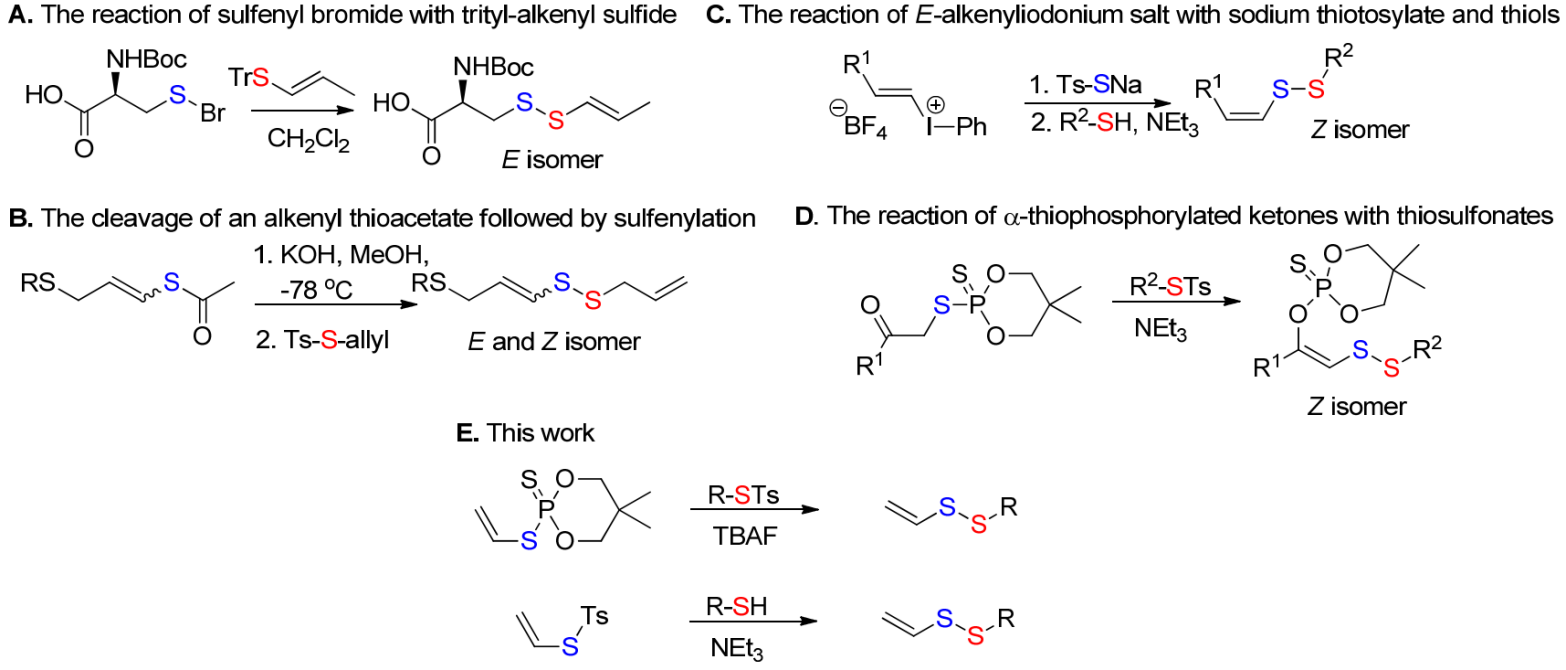
Previously reported methods for the synthesis of alkenyl disulfides (**A**–**D**) and our new synthesis approach (**E**).

Although unsymmetrical disulfides can be obtained by several different synthetic methods, the synthesis of unsymmetrical alkenyl disulfides can be accomplished by only four methods (Scheme 1A–D).

The first method involves the reaction of sulfenyl bromide with trityl-alkenyl sulfide [80] (Scheme 1A). The alkenyl disulfides can also be obtained by the low-temperature cleavage of an alkenyl thioacetate with hydroxide to give alkenethiolate and the subsequent sulfenylation reaction with corresponding *S*-alkyl *p*-toluenethiosulfonate. The appropriate vinyl disulfide was obtained with a high yield after column chromatography in the second method [81,82,83] (Scheme 1B). Unfortunately, the formation of the *E* isomer or a mixture of *Z*/*E* alkenyl disulfides for both methods (Scheme 1A,B) was observed. The synthesis of unsymmetrical *Z*-alkenyl disulfides with additional functional groups can be accomplished with readily available staring materials under mild conditions with moderate to high yields (Scheme 1C). The third method is diastereoselective and an exclusive formation of Z-isomer is observed. The developed method includes the reaction of *E*-alkenyliodonium salt with sodium thiotosylate and thiols in the presence of a base [84]. The fourth method [85] is based on the base-promoted rearrangement of a-thiophosphorylated ketones followed by thioalkylation with thiotosylates (Scheme 1D).

There are a limited amount of synthetic methods available for the synthesis of alkenyl disulfides (Scheme 1). We were interested in the development of an experimentally practical and versatile method to access vinyl disulfides with additional functional groups. The designed method is based on the readily available *S*-vinyl phosphorodithioate and *S*-vinyl thiosulfonate (Scheme 1E).

The synthetic potential of vinyl disulfides can involve formation of complexes with metals, multicomponent reactions, Heck reaction, olefin metathesis, or the variety of cycloaddition reactions. Due to the poor availability of vinyl disulfides, aforementioned transformations has not been examined yet.

## 2. Materials and Methods

Preparation of thiotosylates 1a–1e; 1k; 1m–1n; 1r was described previously [71,85]. All bromides were purchased from ProChimia (Sopot, Poland) and were used for synthesis of required thiotosylates. Sodium 4-methylbenzenesulfenate was purchased from Merck and was used for preparation of sodium 4-methylbenzenesulfonothioate as described previously [85]. Vinyl magnesium bromide solution (1M) in THF (tetrahydrofuran) and tetrabutylammonium fluoride (TBAF) solution (1M) in THF were purchased from Merck. Tetrahydrofuran was pre-dried over KOH pellets and distilled. Subsequently, tetrahydrofuran (THF) was dried by heating under reflux over potassium in the presence of benzophenone as an indicator and distilled. Silica gel plates Supelco UV254 (St. Louis, MS, USA) were used for thin layer chromatography (TLC). A silica gel 60 (230-400 mesh, Merck, Darmstadt, Germany) was used for column chromatography. NMR spectra were recorded on Brucker 400 MHz spectrometers. The residual solvent peak was used as the internal reference (CDCl_3_: δ = 7.26 ppm for ^1^H, δ = 77.0 ppm for ^13^C). Nicolet Is50 Fourier-transform infrared (FT-IR) spectrometer (Wien, Austria) was used to record the IR spectra by attenuated total reflectance (ATR) method. A Gallenkamp 7936B apparatus (Warwick, UK) was used to determine melting points.

### 2.1. Synthesis of 5,5-Dimethyl-2-thioxo-2-vinylsulfanyl-[1,3,2]dioxaphosphorinane

A stirred solution of 868 mg (2.2 mmol) bis-(5,5-dimethyl-2-thioxo-1,3,2-dioxaphosphorinan-2-yl) disulfide in dry THF (3 mL) was cooled to −5 °C under nitrogen, then vinylmagnesium bromide (2.0 mmol, 1M solution in THF, 2 mL) was added dropwise. After complete addition, the mixture was stirred for 15 min at rt, and the solvent was removed in vacuo. Crude product was purified by silica gel column chromatography(petroleum ether/DCM 4:1) to provide 296 mg of *S*-vinyl phosphorodithioate as a white powder with 66% yield.

Chromatography: PE/DCM 4/1 (R*_f_* = 0.2), Yield 0.296 g 66%,white solid, mp. 57.8–58.8 °C

^1^H NMR (400 MHz, CDCl_3_) δ 6.50 (dt, *J* = 16.6, 9.3 Hz, 1 H), 5.79–5.63 (m, 2 H), 4.21 (dd, *J* = 10.8, 7.0 Hz, 2 H), 4.02 (dtd, *J* = 11.2, 2.4, 1.2 Hz, 2 H), 1.29 (s, 3H), 0.97 (s, 3 H).

^13^C NMR (101 MHz, CDCl_3_) δ 124.0 (d, *J* = 4.5 Hz), 123.5 (d, *J* = 12.6 Hz), 77.6 (d, *J* = 9.0 Hz), 32.5 (d, *J* = 7.0 Hz), 21.0 (d, *J* = 1.2 Hz).

^31^P NMR (202 MHz, CDCl_3_) δ 82.46.

HRMS (ESI): m/z [M + H]^+^ calcd for C_7_H_14_O_2_PS_2_: 225.0167; found: 225.0168.

### 2.2. A Typical Procedure for the Preparation of Vinyl Disulfides 2 from S-vinyl Thiotosylate and Representative Analytical Data

To a stirred, ice-cooled solution of *S*-vinyl thiotosylate 428 mg (2.0 mmol) and thiol 4 (1.0 mmol) in dry DCM (10 mL) under nitrogen, NEt_3_ (1.0 mmol, 140 µL) was added in one portion. The mixture was stirred at rt for 15 min. Then, the solvent was evaporated and the reside was purified by column chromatography (SiO_2_) to provide disulfide 2.

1-Vinyldisulfanyldodecane 2a

Chromatography: Hexene (R*_f_* = 0.6), Yield 0.253 g, 97%, colorless oil.

^1^H NMR (400 MHz, CDCl_3_) δ 6.41 (dd, *J* = 16.2, 9.6 Hz, 1 H), 5.56 (d, *J* = 16.2 Hz, 1 H), 5.36 (d, *J* = 9.6 Hz, 1 H), 2.73 (t, *J* = 7.3 Hz, 2 H), 1.74–1.64 (m, 2 H), 1.44–1.26 (m, 18 H), 0.91 (t, *J* = 6.9 Hz, 3 H).

^13^C NMR (101 MHz, CDCl_3_) δ 133.8, 113.1, 38.3, 31.9, 29.6, 29.6, 29.6, 29.5, 29.3, 29.2, 29.1, 28.5, 22.7, 14.1.

HRMS (ESI): m/z [M + H]^+^ calcd for C_14_H_29_S_2_: 261.1705; found: 261.1711.

11-Vinyldisulfanylundecanoic acid methyl ester 2c

Chromatography: Hexene/DCM 2/1(R*_f_* =0.25), Yield 0.256 g, 88%, colorless oil.

^1^H NMR (400 MHz, CDCl_3_) δ 6.40 (dd, *J* = 16.2, 9.6 Hz, 1 H), 5.55 (d, *J* = 16.3 Hz, 1 H), 5.36 (d, *J* = 9.6 Hz, 1 H), 3.69 (s, 3 H), 2.72 (t, *J* = 7.3 Hz, 2 H), 2.32 (t, *J* = 7.5 Hz, 2 H), 1.77–1.62 (m, 4 H), 1.48–1.20 (m, 12 H).

^13^C NMR (101 MHz, CDCl_3_) δ 174.3, 133.8, 113.1, 51.5, 38.2, 34.1, 29.4, 29.3, 29.2, 29.2, 29.1, 29.1, 28.5, 24.9.

HRMS (ESI): m/z [M + H]^+^ calcd for C_14_H_27_O_2_S_2_: 291.1447; found: 291.1452.

### 2.3. A Typical Procedure for the Preparation of benzo[b][1,4]thiazine disulfanyl derivatives 7 and Representative Analytical Data

To a solution of 2-*N*-sulfonylthiophthalimide 5.242 mg (0.5 mmol) and vinyl disulfide 2 (0.75 mmol) in dry CHCl_3_ (20 mL) under nitrogen, triethylamine (0.5 mmol, 70 µL) was added. Mixture was stirred under reflux for 17 h. Then, the solvent was evaporated and the reside was purified by column chromatography (SiO_2_) to provide 7.

3-(Dodec-1-yldisulfanyl)-6,8-dimethoxy-4-(4-toluenesulfonyl)-3,4-dihydro-2*H*-benzo[1,4]thiazine 7a

Chromatography: Hexane/DCM 2/1 (R*_f_* = 0.32), Yield 0.150 g, 50%, thick yellow oil

IR (ATR): 2922(w), 2851(w), 1578(w), 1455(w), 1434(w), 1308(s), 1284(w), 1228(w), 1185(w), 1060(w), 1039(w), 842(s), 829(s), 812(s), 705(w), 694(s), 644(s) cm^−1^

^1^H NMR (400 MHz, CDCl_3_) δ 7.52 (d, *J* = 8.3 Hz, 2 H), 7.21 (d, *J* = 8.1 Hz, 2 H), 7.03 (d, *J* = 2.4 Hz, 1 H), 6.37 (d, *J* = 2.4 Hz, 1 H), 5.89 (t, *J* = 5.2 Hz, 1 H), 3.83 (s, 3 H), 3.83 (s, 3 H), 3.15-2.85 (m, 2 H), 2.87–2.74 (m, 2 H), 2.40 (s, 3 H), 1.71-1.54 (m, 2 H), 1.44–1.21 (m, 18 H), 0.88 (t, *J* = 6.9 Hz, 3 H).

^13^C NMR (101 MHz, CDCl_3_) δ 157.8, 156.0, 144.2, 135.9, 133.4, 129.6, 127.4, 109.2, 105.1, 97.4, 65.4, 56.1, 55.6, 39.2, 31.9, 29.7, 29.7, 29.5, 29.4, 22.7, 21.6, 14.1.

HRMS (ESI): m/z [M + H]^+^ calcd for C_29_H_44_NO_4_S_4_: 598.2148; found: 598.2153.

Synthesis of starting materials, vinyl disulfides 2 and benzo[b][1,4]thiazine disulfanyl derivatives 7 with analytical data, copy of IR, and NMR spectra are in the Supplementary Materials.

## 3. Results and Discussion

The corresponding *S*-vinyl phosphorodithioate was obtained by the reaction of bis-(5,5-dimethyl-2-thiono-1,3,2-dioxaphosphorinanyl)disulfide with vinylmagnesium bromide in THF with 66% yield. We examined several methods to prepare *S*-vinyl thiotosylate. The most effective reaction was the reaction of ditosylsulfide (1,3-di-*p*-toluene-trisulfane-1,1,3,3-tetraoxide) with vinylmagnesium bromide in THF at −78 °C to produce the required *S*-vinyl thiotosylate with 60% yield.

The first method developed for the preparation of unsymmetrical vinyl disulfides with additional functional groups included the reaction of *S*-vinyl phosphorodithioate with thiotosylates 1 in the presence of tetrabutylammonium fluoride (TBAF) in THF at 0 °C for 15 min. We selected a variety of thiotosylates 1a–r to determine the limitations and scope of the designed transformation. Compound 1 contained alkyl and aryl groups with additional thioacetyl, ester, protected amino, nitro or carbon–carbon double-bond functionalities. The results are presented in Table 1.

Although vinyl disulfides 2a–i were obtained with high or very high yields of 62–93% (entries 1–9), other vinyl disulfides 2j–r could not be obtained by the developed method. We noticed that thiosulfonate 1 could be converted to symmetrical disulfide 3 in the presence of TBAF when *S*-vinyl phosphorodithioate was not added. The success of the above method depended on the rate of the reaction of fluoride anion with *S*-vinyl phosphorodithioate and thiotosylate. When the reaction of the fluoride anion with *S*-vinyl phosphorodithioate was faster than the reaction with thiotosylate, the corresponding vinylthiolate anion was generated, and the subsequent reaction with thiotosylate provided vinyl disulfide 2. However, when the reaction of the fluoride anion with thiotosylate was faster, symmetrical disulfide 3 was produced. As shown in Table 1, the developed method is efficient for alkyl thiosulfonates. In the case of aryl- or benzyl-type thiosulfonates, the corresponding symmetrical disulfides 3 were produced exclusively.

We developed another method for the synthesis of unsymmetrical vinyl disulfides to overcome the above limitations. The transformation comprises the reaction of *S*-vinyl thiotosylate with thiols 4 in the presence of NEt_3_ at room temperature. The obtained results are presented in Table 2.

As shown in Table 2, the corresponding functionalized unsymmetrical vinyl disul-fides 2a–t were obtained with very high yields of 80–98%. The developed method is ef-fective for alkyl-vinyl disulfides 2a and 2c (entries 1,2) and for disulfides 2j–r, which could not be obtained with *S*-vinyl phosphorodithioate (Table 1 entries 10–17). The developed method is more convenient and versatile. The method allows for a broad range of products to be accessed, and all starting materials are readily available.

Benzo[*b*][1,4]thiazine is a valuable heterocyclic system with promising and wide applications in medical chemistry [86,87]. We decided to explore the possibility of benzo[*b*][1,4]thiazine derivative synthesis with a disulfide functionality. The het-ero-Diels–Alder reaction [88] is the most convenient approach for the synthesis of benzo[*b*][1,4]thiazine derivatives based on the generation of transient *o*-iminothioquinone 6 from 2-*N*-sulfonylthiophthalimides 5 and subsequent reaction with vinyl disulfides 2 in an inverse electron-demand [4+2] cycloaddition to produce compounds 7. The preliminary results are summarized in Table 3.

Although the reaction conditions were not optimized, the corresponding benzo[*b*][1,4]thiazine disulfanyl derivatives 7 were obtained with moderate yields of 25–50%. Moreover, there is no alternative method that allows for the preparation of compounds 7a, 7c, 7m, 7n, 7r. The recovered vinyl disulfides 2 demonstrated the possibility of improving the yield of product 7 by prolonging the reaction time or selecting a solvent with a higher boiling point. The optimal conditions, scope of starting materials and stereoselectivity of the hetero-Diels-Alder reaction are under investigation.

## 4. Conclusions

In summary, we developed a convenient and experimentally practical method for preparing unsymmetrical vinyl disulfides with additional functional groups under mild conditions. The method is based on readily available starting materials. The applied mild reaction conditions tolerate a variety of additional functionalities, including esters, carboxy, carbon–carbon double bonds, and protected amino, nitro, cyano, and hydroxy groups. We demonstrated that functionalized unsymmetrical vinyl disulfides can be used in the inverse electron-demand [4+2] hetero-Diels–Alder reaction to produce benzo[*b*][1,4]thiazine disulfanyl derivatives.

## Figures and Tables

**Table 1:**
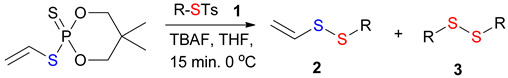
Synthesis of vinyl disulfides **2** from S-vinyl phosphorodithioate.

Entry ^1^	R	Yield (%) ^2^	Yield (%) ^2^
1	–n–C_12_H_25_ 1a	93 2a	-
2	–(CH_2_)_9_CH=CH_2_ 1b	82 2b	-
3	–(CH_2_)_10_COOMe 1c	73 2c	-
4	–(CH_2_)_11_OMe 1d	62 2d	-
5	–(CH_2_)_11_SAc 1e	85 2e	-
6	–(CH_2_)_2_NHBoc 1f	75 2f	-
7	–(CH_2_)_2_C_6_H_4_–4–CH_3_ 1g	76 2g	-
8	–(CH_2_)_2_–3–indyl 1h	75 2h	-
9	–(CH_2_)_2_C_6_H_4_–4–CF_3_ 1i	65 2i	-
10	–(CH_2_)_2_C_6_H_4_–4–F 1j	-	100 3j
11	–C_6_H_4_–4–CH_3_ 1k	-	100 3k
12	–CH_2_–2–naphthyl 1l	-	80 3l
13	–CH_2_C_6_H_4_–4–NO_2_ 1m	-	70 3m
14	–CH_2_C_6_H_4_–4–OMe 1n	-	85 3n
15	–CH_2_C_6_H_4_–4–CN 1o	-	75 3o
16	–(CH_2_)_2_C_6_H_4_–4–OMe 1p	-	86 3p
17	–CH_2_Ph 1r	-	76 3r

^1^ Reaction conditions: TBAF (1.1 mmol) was added to a solution of S-vinyl phosphorodithioate (1.0 mmol) and thiotosylate **1** (1.0 mmol) in dry THF (5 mL) at 0 °C. A mixture was stirred for 15 min under a N_2_ atmosphere at 0 °C. ^2^ Isolated yields.

**Table 2:**
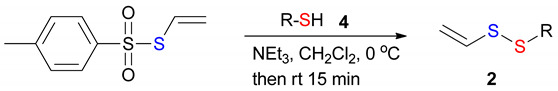
Synthesis of vinyl disulfides 2 from *S*-vinyl thiotosylate.

Entry ^1^	R ^1^	Yield (%) ^2^
1	–*n*–C_12_H_25_ 4a	97 2a
2	–(CH_2_)_10_COOMe 4c	88 2c
3	–(CH_2_)_2_C_6_H_4_–4–F 4j	90 2j
4	–C_6_H_4_–4–CH_3_ 4k	96 2k
5	–CH_2_–2–naphthyl 4l	92 2l
6	–CH_2_C_6_H_4_–4–NO_2_ 4m	80 2m
7	–CH_2_C_6_H_4_–4–OMe 4n	87 2n
8	–CH_2_C_6_H_4_–4–CN 4o	89 2o
9	–CH_2_Ph 4r	98 2r
10	–(CH_2_)_10_COOH 4s	84 2s
11	–(CH_2_)_11_OH 4t	91 2t

^1^ Reaction conditions: NEt_3_ (1.0 mmol) was added to a solution of *S*-vinyl thiotosylate (2.0 mmol) and thiol **4** (1.0 mmol) in dry CH_2_Cl_2_ (10 mL) at 0 °C. Then the mixture was stirred for 15 min under a N_2_ atmosphere at room temperature. ^2^ Isolated yields.

**Table 3:**
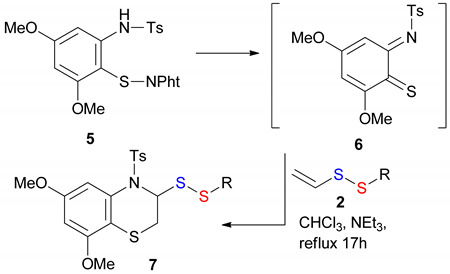
Synthesis of benzo[*b*][1,4]thiazine disulfanyl derivatives **7**.

Entry ^1^	R	Yield (%) ^2^	Recovered 2 (%) ^2^
1	–n–C_12_H_25_ 2a	50 7a	35 2a
2	–(CH_2_)_10_COOMe 2c	30 7c	42 2c
3	–CH_2_C_6_H_4_–4–NO_2_ 2m	29 7m	46 2m
4	–CH_2_C_6_H_4_–4–OMe 2n	27 7n	44 2n
5	–CH_2_Ph 2r	25 7r	52 2r

^1^ Reaction conditions: A solution of 2-*N*-sulfonylthiophthalimides **5** (0.5 mmol), vinyl disulfide **2** (0.75 mmol) and NEt_3_ (0.5 mmol) in dry CHCl_3_ (20 mL) was refluxed for 17 h under N_2_ atmosphere. ^2^ Isolated yields.

## Data Availability

Data sharing is not applicable.

## Supplementary Materials

The following are available online at https://www.mdpi.com/1996-1944/14/6/1342/s1: Synthesis of starting materials, vinyl disulfides 2, and benzo[*b*][1,4]thiazine disulfanyl derivatives **7** with analytical data, copy of IR, and NMR spectra.
