# Supplementary material for: Convenient Synthesis of Functionalized Unsymmetrical Vinyl Disulfides and Their Inverse Electron-Demand Hetero-Diels-Alder Reaction
Source: Materials (Basel). 2021 Mar 10;14(6):1342. doi: 10.3390/ma14061342 (PMC7998207; doi:10.3390/ma14061342)
Supplement: Supplementary file 1 [file materials-14-01342-s001.pdf]

Convenient synthesis of functionalized unsymmetrical vinyl disulfides and their inverse-electron-demand hetero-Diels-Alder reaction

Supporting Information

Bartosz Jędrzejewski, Mateusz Musiejuk, Justyna Doroszuk and Dariusz Witt*

Department of Organic Chemistry, Faculty of Chemistry, Gdansk University of Technology, Narutowicza 11/12, 80-233 Gdansk, Poland

Fax +48(58)3472694; E-mail: dariusz.witt@pg.edu.pl

Abstract: We developed simple and efficient methods for the synthesis of functionalized unsymmetrical vinyl disulfides under mild conditions with moderate to high yields. The designed methods are based on the reaction of *S*-vinyl phosphorodithioate with thiotosylates or *S*-vinyl thiotosylates with thiols. The developed methods allow for the preparation of unsymmetrical vinyl disulfides with additional hydroxy, carboxy, protected amino or ester functionalities. Vinyl disulfides reacted with the generated transient *o*-iminothioquinones in an inverse electron-demand [4+2] cycloaddition to afford benzo[*b*][1,4]thiazine derivatives.

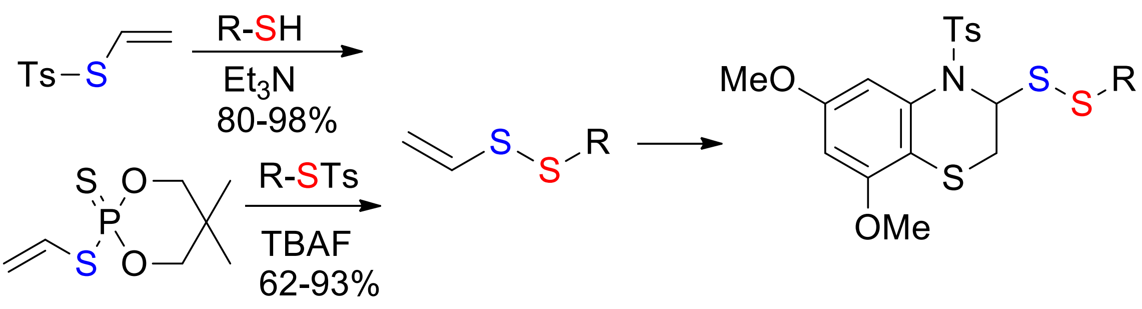

R = -*n*-$C_{12}H_{25}$ -$(CH_2)_9CH{=}CH_2$ -$(CH_2)_{10}COOMe$ -$(CH_2)_{11}OMe$ -CH_2Ph -$(CH_2)_{11}SAc$ -$(CH_2)_2NHBoc$ -$(CH_2)_2$-3-indyl -$(CH_2)_2C_6H_4$-4-CF_3 -$(CH_2)_2C_6H_4$-4-F -C_6H_4-4-CH_3 -CH_2-2-naphthyl -$CH_2C_6H_4$-4-NO_2 -$CH_2C_6H_4$-4-OMe -$(CH_2)_{11}OH$ -$CH_2C_6H_4$-4-CN -$(CH_2)_{10}COOH$

Table of Contents

Contents

General information.	S3
Experimental Procedures	S3
General procedure for the preparation of thiotosylates derivatives 1f-j;1l;1p.	S3
Synthesis of *N*-Boc cysteamine thiotosylate 1f	S4
Synthesis of vinyl disulfide 2 from S-vinyl phophorodithioate	S4
Synthesis of vinyl disulfide 2 from S-vinyl thiotosylate	S5
General procedure for synthesis of benzo[*b*][1,4]thiazine disulfanyl derivatives 7	S6
Spectral characterization of thiotosylates derivatives 1f-j;1l;1p	S7
Spectral characterization of starting materials	S8
Spectral characterization of vinyl disulfides 2	S9
Spectral characterization of benzo[b][1,4]thiazine disulfanyl derivatives 7a-r	S12
References	S14
Author Contributions	S14
NMR SPECTRA	S14

General information.

Preparation of thiotosylates **1a-1e; 1k; 1m-1n; 1r** was described in previous works.[1] All bromides were purchased from ProChimia, preparation of sodium 4-methylbenzenesulfonothioate from sodium 4-methylbenzenesulfonate purchased from Merck, was described previously.[1] Vinyl magnesium bromide solution (1M) in THF and tetrabutylammonium fluoride (TBAF) solution (1M) in THF were purchased from Merck.
Tetrahydrofuran was pre-dried over KOH pellets and distilled. Then it was dried by heating under reflux over potassium in the presence of benzophenone as an indicator. Chloroform, acetonitrile and dichloromethane were dried according to literature procedure. TLC was performed with silica gel Polygram SIL G/UV254. Column chromatography was performed using silica gel 60 (230-400 mesh, Merck).
NMR spectra were recorded on Brucker 400 MHz spectrometers. The residual solvent peak was used as the internal reference ($CDCl_3$: δ=7.26 ppm for ^1H, δ=77.0 ppm for ^{13}C). IR spectra were recorded on Nicolet Is50 Ft-IR spectrometer by ATR method. Melting points were measured with a Gallenkamp 7936B apparatus and were not standardized.

Experimental Procedures

General procedure for the preparation of thiotosylates derivatives 1f-j;1l;1p.

From alkyl halides[1]

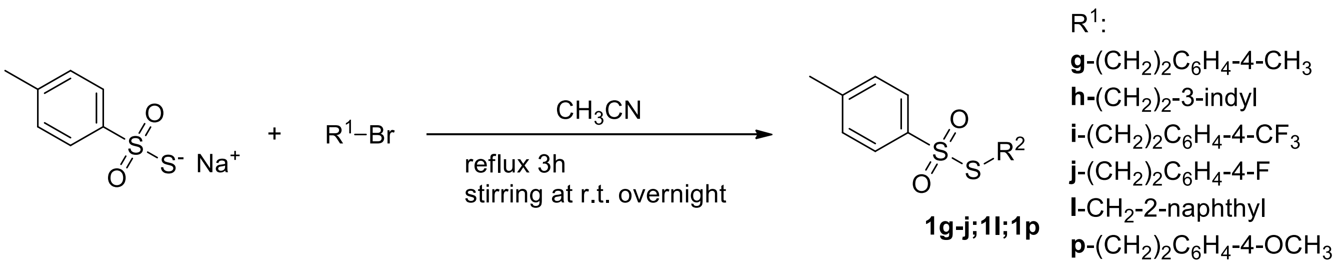

To the solution of alkyl halide (1 eq, 14.7 mmol) in dry acetonitrile (50 mL) under reflux condenser sodium 4-methylbenzenesulfonotioate (1 eq, 14.7 mmol) was added, reaction was refluxed 3 h, then was stirred overnight at rt. After this time solvent was evaporated, residue was washed with water (30 mL), and extracted 3 times with diethyl ether (50 mL). Combined organic layers were dried over $MgSO_4$, filtered and evaporated to constant volume. Crude product was purified by column chromatography (SiO_2). Pure compounds were identified by ^1H NMR and ^{13}C NMR spectra. Results are summarized in Table S1.

Table S1. Preparation of thiotosylates **1**

Entry	Halide	Yield (%)[a]
1	Br-CH_2CH_2-C_6H_4-4-CH_3	80
2	Br-CH_2CH_2-3-indyl	87
3	Br-CH_2CH_2-C_6H_4-4-CF_3	75
4	Br-CH_2CH_2-C_6H_4-4-F	70
5	Br-CH_2-2-naphthyl	78
6	Br-CH_2CH_2-C_6H_4-4-OCH_3	80

[a] Isolated yield

Synthesis of *N*-Boc cysteamine thiotosylate 1f

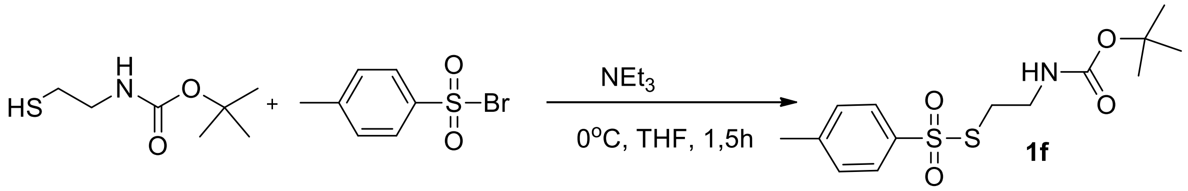

A solution of 564 mg of tosyl bromide (2.4 mmol) and triethylamine (2.4 mmol, 334 μL) in dry THF (20 mL) was cooled to 0°C under nitrogen. Then a solution of 354 mg *N*-Boc cysteamine (2.0 mmol) in dry THF (5 mL) was added for 30 minutes. Mixture was stirred at 0°C for 1 h after complete addition. Then solvent was removed in vacuo and the residue was purified by column chromatography (SiO_2) using DCM as eluent to provide 464 mg of *S*-(2-((tert-butoxycarbonyl)amino)ethyl) 4-methylbenzenesulfonothioate as a white solid with 70% yield.

Synthesis of vinyl disulfides 2 from *S*-vinyl phophorodithioate

a. Synthesis of 5,5-Dimethyl-2-thioxo-2-vinylsulfanyl-[1,3,2]dioxaphosphorinane

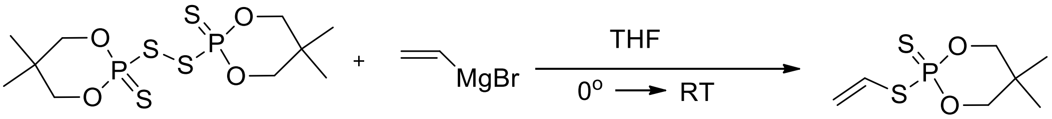

To a stirred solution of 868 mg (2.2 mmol) bis-(5,5-dimethyl-2-thioxo-1,3,2-dioxaphosphorinan-2-yl) disulfide in dry THF (3 mL) was cooled to -5°C under nitrogen, then vinylmagnesium bromide (2.0 mmol, 1M solution in THF, 2 mL) was added dropwise. After complete addition, mixture was stirred for 15 min at rt, and the solvent was removed in vacuo. Crude product was purified by silica gel column chromatography(petroleum ether/DCM 4:1) to provide 296 mg of *S*-vinyl phosphorodithioate as white powder with 66% yield.

b. General procedure for synthesis of vinyl disulfide 2 form *S*-vinyl phosphorodithioate

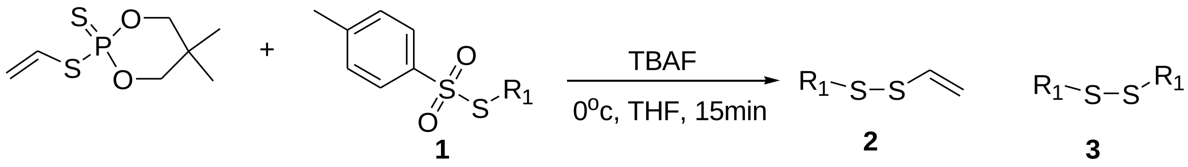

A stirred solution of 224 mg (1.0 mmol) *S*-vinyl phosphorodithioate and thiotosylate **1** (1.0 mmol) in dry THF (5mL) under nitrogen was cooled to 0°C. Then tetrabutylammonium fluoride (1.1 mmol, 1M solution in THF, 1.1 mL) was added in one portion. After addition, mixture was stirred for 15 min at 0°C. Solvent was removed in vacuo and crude product was purified by column chromatography (SiO_2). Results are summarized in Table S2.

Table S2. Synthesis of vinyl disulfide 2 from S-vinyl phosphorodithioate

Entry	R	Yield **2** (%) [a]	Yield **3** (%)[a]
1	$-(CH_2)_{11}CH_3$ **1a**	93 **2a**	-
2	$-(CH_2)_9CH{=}CH_2$ **1b**	82 **2b**	-
3	$-(CH_2)_{10}COOCH_3$ **1c**	73 **2c**	-
4	$-(CH_2)_{11}OCH_3$ **1d**	62 **2d**	-
5	$-(CH_2)_{11}SCOCH_3$ **1e**	85 **2e**	-
6	$-(CH_2)_2$NHBOC **1f**	75 **2f**	-
7	$-(CH_2)_2C_6H_4$-4-CH_3 **1g**	76 **2g**	-
8	$-(CH_2)_2$-3-indyl **1h**	75 **2h**	-
9	$-(CH_2)_2C_6H_4$-4-CF_3 **1i**	65 **2i**	-

10	-$(CH_2)_2C_6H_4$-4-F **1j**	-	100 **3j**
11	-C_6H_4-4-CH_3 **1k**	-	100 **3k**
12	-CH_2-2-naphthyl **1l**	-	80 **3l**
13	-$CH_2C_6H_4$-4-NO_2 **1m**	-	70 **3m**
14	-$CH_2C_6H_4$-4-OCH_3 **1n**	-	85 **3n**
15	-$CH_2C_6H_4$-4-CN **1o**	-	75 **3o**
16	-$(CH_2)_2C_6H_4$-4-OCH_3 **1p**	-	86 **3p**
17	-$CH_2C_6H_5$ **1r**	-	76 **3r**

[a] Isolated yield

Synthesis of vinyl disulfide 2 from *S*-vinyl thiotosylate

a. Synthesis of bis (p-toluenesulfonyl)sulfide

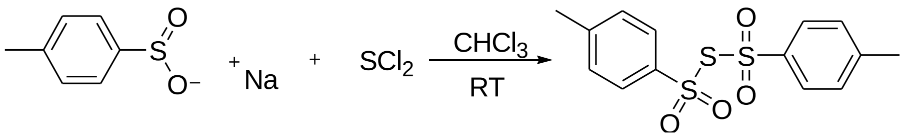

Ditosylsulfide was prepared using modified literature procedure[2]:

To a suspension of 13.54 g (76 mmol) sodium p-toluenesulifnate in dry $CHCl_3$ (150 mL) under N_2, sulfur dichloride (40 mmol, 2.54 mL) was added dropwise. Mixture was stirred for 2 h at rt, then the insoluble material was filtered off. Solvent was removed in vacuo, obtained yellowish solid was recrystallized from boiling glacial acetic acid to provide 9.24g (68%) of bis (p-toluenesulfonyl)sulfide as white needles (mp. 136-137°C, lit. 137°C), spectra were identical as reported in literature[2].

b. Synthesis of S-vinyl thiotosylate

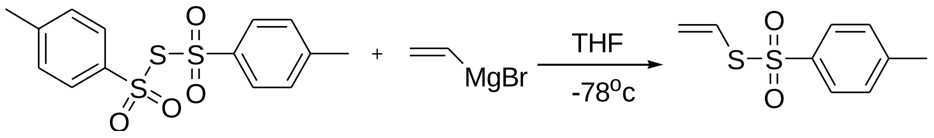

Vinylmagnesium bromide (10 mmol, 1M solution in THF, 10 mL) was added dropwise to a stirred solution of bis-(p-toluenesulfonyl)sulfide 5.47 g (16 mmol) in dry THF (200 mL) at -78°C under nitrogen . After the complete addition, mixture was stirred for 2 h at this temperature, then cooling bath was removed, and mixture was warmed to rt. Solvent was removed in vacuo, and the residue was purified by column chromatography (Hexene : DCM 2:1) to provide 1.29 g (60%) of *S*-vinyl thiotosylate as a yellow oil.

c. General procedure for synthesis of vinyl disulfides 2 form S-vinyl thiotosylate

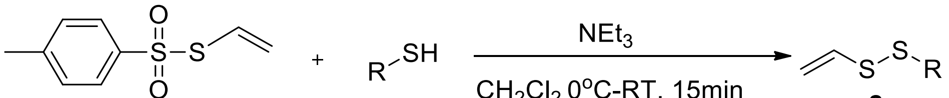

4 **2**

To stirred, ice cooled solution of *S*-vinyl thiotosylate 428 mg (2.0 mmol) and thiol **4** (1.0 mmol) in dry DCM (10 mL) under nitrogen, NEt_3 (1.0 mmol, 140μL) was added in one portion. The mixture was stirred at rt for 15 min. Then solvent was evaporated and the reside was purified by column chromatography (SiO_2) to provide disulfide **2**. Results are summarized in Table S3.

Table S3. Synthesis of vinyl disulfides **2** from *S*-vinyl thiotosylate

Entry	R	Yield 2 (%)[a]
1	-$(CH_2)_{11}CH_3$ **4a**	97 **2a**
2	-$(CH_2)_{10}COOCH_3$ **4c**	88 **2c**
3	-$(CH_2)_2C_6H_4$-4-F **4j**	90 **2j**
4	-C_6H_4-4-CH_3 **4k**	96 **2k**
5	-CH_2-2-naphthyl **4l**	92 **2l**
6	-$CH_2C_6H_4$-4-NO_2 **4m**	80 **2m**
7	-$CH_2C_6H_4$-4-OCH_3 **4n**	87 **2n**
8	-$CH_2C_6H_4$-4-CN **4o**	89 **2o**
9	-$CH_2C_6H_5$ **4r**	98 **2r**
10	-$(CH_2)_{10}COOH$ **4s**	84 **2s**
11	-$(CH_2)_{11}OH$ **4t**	91 **2t**

[a] Isolated yields.

General procedure for synthesis of benzo[*b*][1,4]thiazine disulfanyl derivatives 7

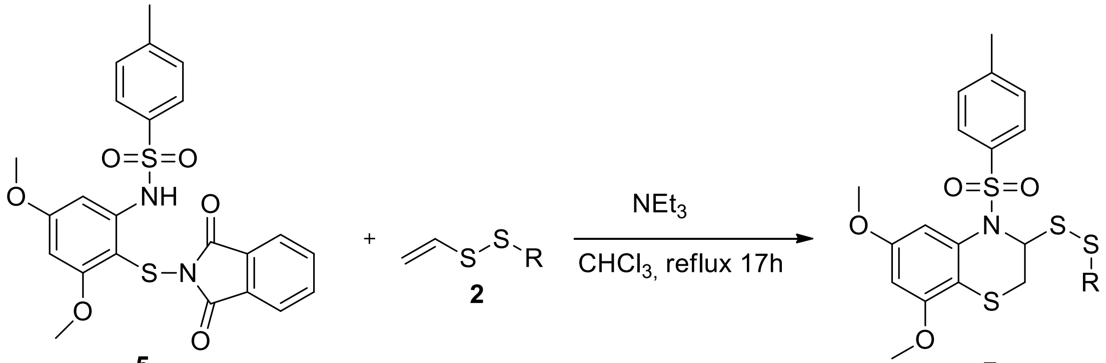

5

7

Benzo[b][1,4]thiazine disulfanyl derivatives were prepared using modified procedure.[3]

To a solution of 2-*N*-sulfonylthiophthalimide **5** 242 mg (0.5 mmol) and vinyl disulfide **2** (0.75 mmol) in dry $CHCl_3$ (20 mL) under nitrogen, triethylamine (0.5 mmol, 70μL) was added. Mixture was stirred under reflux for 17 h, then solvent was removed in vacuo, and the crude product was purified by column chromatography (SiO_2). Results are summarized in Table S4.

Table S4. Synthesis of benzo[*b*][1,4]thiazine disulfanyl derivatives **7**

Entry	R	Yield **7** (%)[a]	Recovered **2** (%)[a]
1	-$(CH_2)_{11}CH_3$	50 **7a**	35 **2a**
2	-$(CH_2)_{10}COOCH_3$	30 **7c**	42 **2c**
3	-$CH_2C_6H_4$-4-NO_2	29 **7m**	46 **2m**
4	-$CH_2C_6H_4$-4-OCH_3	27 **7n**	44 **2n**
5	-$CH_2C_6H_5$	25 **7r**	52 **2r**

[a] Isolated yields.

Spectral characterization of thiotosylates derivatives 1f-j;1l;1p

S-(2-((tert-butoxycarbonyl)amino)ethyl) 4-methylbenzenesulfonothioate 1f

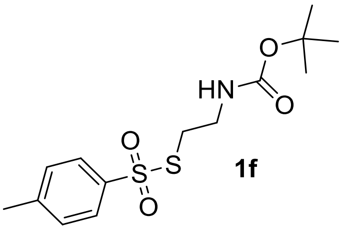

1f

Chromatography: DCM (R_f=0.3), Yield 464 mg 70%, white solid, mp. 120-121°C

1H NMR (400 MHz, $CDCl_3$) δ 7.84 (d, *J* = 8.4 Hz, 2H), 7.37 (d, *J* = 8.5 Hz, 2H), 4.93 (s, 1H), 3.47 – 3.34 (m, 2H), 3.12 (t, *J* = 6.3 Hz, 2H), 2.48 (s, 3H), 1.45 (s, 9H).

13C NMR (101 MHz, $CDCl_3$) δ 155.7, 145.0, 141.7, 130.0, 127.1, 79.8, 39.7, 36.1, 28.3, 21.7.

HRMS (ESI): m/z $[M + H]^+$ calcd for $C_{14}H_{22}NO_4S_2$: 332.0985; found: 332.0990.

S-2-(4-methylphenyl)eth-1-yl 4-methylbenzenesulfonothioate 1g

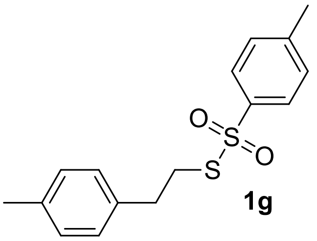

1g

Chromatography : H/DCM 1/1 (R_f=0.28), Yield 3.60g 80%, white solid, mp. 60-61°C

^{1}H NMR (400 MHz, $CDCl_3$) δ 7.84 (d, *J* = 8.4 Hz, 2H), 7.36 (d, *J* = 8.0 Hz, 2H), 7.11 (d, *J* = 7.8 Hz, 2H), 7.01 (d, *J* = 8.0 Hz, 2H), 3.22 (t, *J* = 7.7 Hz, 2H), 2.89 (t, *J* = 7.7Hz, 2H),), 2.48 (s, 3H), 2.34 (s, 3H).

^{13}C NMR (101 MHz, $CDCl_3$) δ 144.7, 142.1, 136.5, 135.7, 129.9, 129.3, 128.4, 127.1, 37.3, 34.7, 21.7, 21.1.

HRMS (ESI): m/z $[M + H]^+$ calcd for $C_{16}H_{19}O_2S_2$: 307.0821; found: 307.0824.

S-(2-(1*H*-indol-3-yl)ethyl) 4-methylbenzenesulfonothioate 1h

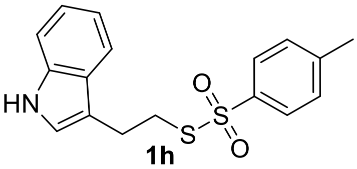

1h

Chromatography : H/DCM 1/1 (R_f=0.25), Yield 3.65g 75%, yellow oil, store at -10°C

^{1}H NMR (400 MHz, $CDCl_3$) δ 8.07 (s, 1H), 7.85 (d, *J* = 8.3 Hz, 2H), 7.52 (d, *J* = 7.9 Hz, 1H), 7.38 (d, *J* = 8.1 Hz, 1H), 7.34 (d, *J* = 8.0 Hz, 1H), 7.26 – 7.20 (m, 1H), 7.17 – 7.11 (m, 1H), 6.99 (d, *J* = 2.3 Hz, 1H), 3.33 (t, *J* = 7.5 Hz, 2H), 3.12 (t, *J* = 7.5 Hz, 2H), 2.48 (d, *J* = 6.6 Hz, 3H).

^{13}C NMR (101 MHz, $CDCl_3$) δ 144.7, 142.1, 136.2, 129.9, 127.0, 126.8, 122.3, 119.6, 118.5, 113.2, 111.3, 36.6, 25.1, 21.7.

HRMS (ESI): m/z $[M + H]^+$ calcd for $C_{17}H_{18}NO_2S_2$: 332.0773; found: 332.0774.

S-2-(4-trifluoromethylphenyl)eth-1-yl 4-methylbenzenesulfonothioate 1i

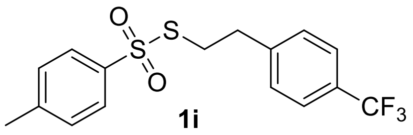

1i

Chromatography : H/DCM 2/1 (R_f=0.27), Yield 3.97g 75%, white solid, mp. 48-49°C

^{1}H NMR (400 MHz, $CDCl_3$) δ 7.83 (d, *J* = 8.3 Hz, 2H), 7.55 (d, *J* = 8.1 Hz, 2H), 7.37 (d, *J* = 8.1 Hz, 2H), 7.24 (d, *J* = 8.0 Hz, 2H), 3.25 (t, *J* = 7.6 Hz, 2H), 3.02 (t, *J* = 7.6 Hz, 2H), 2.48 (s, 3H).

^{13}C NMR (101 MHz, $CDCl_3$) δ 145.0, 142.7, 141.9, 129.9, 129.4, 129.0, 127.1, 125.6 (q, *J* = 3.8 Hz), 122.8, 36.7, 35.0, 21.7.

HRMS (ESI): m/z $[M + H]^+$ calcd for $C_{16}H_{16}F_3O_2S_2$: 361.0538; found: 361.0541.

S-2-(4-fluorophenyl)eth-1-yl 4-methylbenzenesulfonothioate 1j

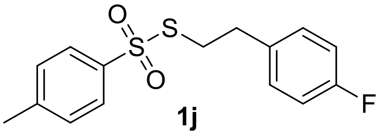

1j

Chromatography : H/DCM 1/1 (R_f=0.3), Yield 3.19g 70%, white solid, mp. 43-44°C

^{1}H NMR (400 MHz, $CDCl_3$) δ 7.83 (d, *J* = 8.4 Hz, 2H), 7.37 (d, *J* = 8.0 Hz, 2H), 7.11 – 7.05 (m, 2H), 7.02 – 6.93 (m, 2H), 3.22 (t, *J* = 7.6 Hz, 2H), 2.92 (t, *J* = 7.6 Hz, 2H), 2.48 (s, 3H).

^{13}C NMR (101 MHz, $CDCl_3$) δ 161.8 (d, *J* = 245.2Hz), 144.9, 142.0, 134.4 (d, *J* = 3.3Hz), 130.1 (d, *J* = 3.3 Hz) , 129.9, 127.0, 115.5 (d, *J* = 21.3 Hz), 37.2, 34.4, 21.7.

HRMS (ESI): m/z $[M + H]^+$ calcd for $C_{15}H_{16}FO_2S_2$: 311.0570; found: 311.0575.

***S*-(naphthalen-2-ylmethyl) 4-methylbenzenesulfonothioate 1l**

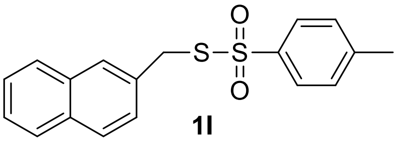

1l

Chromatography : H/DCM 1/1 (R_f=0.35), Yield 3.72g 77%, white solid, mp. 100-101°C

^{1}H NMR (400 MHz, $CDCl_3$) δ 7.81 – 7.76 (m, 1H), 7.73 – 7.66 (m, 4H), 7.60 (s, 1H), 7.52 – 7.46 (m, 2H), 7.30 – 7.26 (m, 1H), 7.13 (d, *J* = 8.5 Hz, 2H), 4.45 (s, 2H), 2.32 (s, 3H).

^{13}C NMR (101 MHz, $CDCl_3$) δ 144.5, 142.1, 133.1, 132.8, 130.9, 129.5, 128.7, 128.2, 127.7, 127.6, 127.0, 126.5, 126.4, 126.3, 40.7, 21.5.

HRMS (ESI): m/z [M + H]+calcd for $C_{18}H_{17}O_2S_2$: 329.0664; found: 329.0665.

***S*-2-(4-methoxyphenyl)eth-1-yl 4-methylbenzenesulfonothioate 1p**

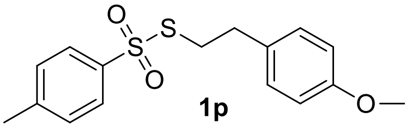

1p

Chromatography : H/DCM 1/2 (R_f=0.32), Yield 3.65g 77%, colorless oil, stored at -10°C

^{1}H NMR (400 MHz, $CDCl_3$) δ 7.88 – 7.77 (m, 2H), 7.41 – 7.31 (m, 2H), 7.11 – 6.99 (m, 2H), 6.91 – 6.73 (m, 2H), 3.81 (s, 3H), 3.21 (t, *J* = 7.6 Hz, 2H),), 2.87 (t, *J* = 7.6 Hz, 2H),), 2.48 (s, 3H).

^{13}C NMR (101 MHz, $CDCl_3$) δ 158.5, 144.8, 142.1, 130.8, 129.9, 129.6, 127.1, 114.0, 55.3, 37.5, 34.3, 21.7.

HRMS (ESI): m/z $[M + H]^+$ calcd for $C_{16}H_{19}O_3S_2$: 323.0770; found: 323.0773.

Spectral characterization of starting materials

5,5-Dimethyl-2-thioxo-2-vinylsulfanyl-[1,3,2]dioxaphosphorinane

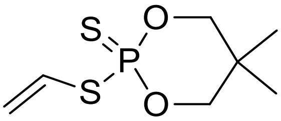

Chromatography: PE/DCM 4/1 (R_f=0.2), Yield 0.296g 66%,white solid, mp. 57.8-58.8 °C

^{1}H NMR (400 MHz, $CDCl_3$) δ 6.50 (dt, *J* = 16.6, 9.3 Hz, 1H), 5.79 – 5.63 (m, 2H), 4.21 (dd, *J* = 10.8, 7.0 Hz, 2H), 4.02 (dtd, *J* = 11.2, 2.4, 1.2 Hz, 2H), 1.29 (s, 3H), 0.97 (s, 3H).

^{13}C NMR (101 MHz, $CDCl_3$) δ 124.0 (d, *J* = 4.5 Hz), 123.5 (d, *J* = 12.6 Hz), 77.6 (d, *J* = 9.0 Hz), 32.5 (d, *J* = 7.0 Hz), 21.0 (d, *J* = 1.2 Hz).

^{31}P NMR (202 MHz, $CDCl_3$) δ 82.46.

HRMS (ESI): m/z $[M + H]^+$ calcd for $C_7H_{14}O_2PS_2$: 225.0167; found: 225.0168.

toluene-4-thiosulfonic acid *S*-vinyl ester

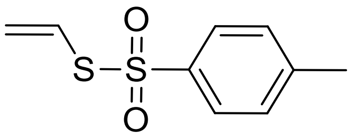

Chromatography: PE/DCM 2/1 (R_f=0.3), Yield 1.29g 60%,yellow oil, stored at -10 °C

^{1}H NMR (400 MHz, $CDCl_3$) δ 7.79 (d, *J* = 8.4 Hz, 2H), 7.35 (d, *J* = 8.1 Hz, 2H), 6.62 (dd, *J* = 16.5, 9.0 Hz, 1H), 5.78 (d, *J* = 9.0 Hz, 1H), 5.61 (d, *J* = 16.5 Hz, 1H), 2.47 (s, 3H).

^{13}C NMR (101 MHz, $CDCl_3$) δ 145.0, 141.4, 129.7, 128.0, 127.3, 125.5, 21.7.

HRMS (ESI): m/z $[M + H]^+$ calcd for $C_9H_{11}O_2S_2$: 215.0195; found: 215.0196.

Spectral characterization of vinyl disulfides 2

1-vinyldisulfanyldodecane 2a

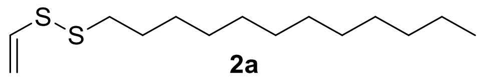

2a

Chromatography: Hexene (R_f=0.6), Yield 0.242g, 93% (Table S2 entry 1); 0.253g, 97% (Table S3 entry 1), colorless oil

^{1}H NMR (400 MHz, $CDCl_3$) δ 6.41 (dd, *J* = 16.2, 9.6 Hz, 1H), 5.56 (d, *J* = 16.2 Hz, 1H), 5.36 (d, *J* = 9.6 Hz, 1H), 2.73 (t, *J* = 7.3 Hz, 2H), 1.74–1.64 (m, 2H), 1.44–1.26 (m, 18H), 0.91 (t, *J* = 6.9 Hz, 3H).

^{13}C NMR (101 MHz, $CDCl_3$) δ 133.8, 113.1, 38.3, 31.9, 29.6, 29.6, 29.6, 29.5, 29.3, 29.2, 29.1, 28.5, 22.7, 14.1.

HRMS (ESI): m/z $[M + H]^+$ calcd for $C_{14}H_{29}S_2$: 261.1705; found: 261.1711.

11-vinyldisulfanylundec-1-ene 2b

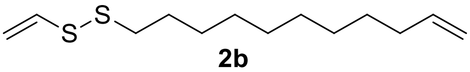

2b

Chromatography: Hexene (R_f=0.57), Yield 0.200g, 82% (Table S2 entry 2), colorless oil

^{1}H NMR (400 MHz, $CDCl_3$) δ 6.41 (dd, *J* =16.2, 9.6 Hz, 1H), 5.92–5.74 (m, 1H), 5.56 (d, *J* =16.2 Hz, 1H), 5.36 (d, *J* =9.6 Hz, 1H), 5.06–4.93 (m, 2H), 2.73 (t, *J* =7.3 Hz, 2H), 2.10–2.03 (m, 2H), 1.74–1.64 (m, 2H), 1.46–1.28 (m, 12H).

^{13}C NMR (101 MHz, $CDCl_3$) δ 139.2, 133.8, 114.1, 113.1, 38.3, 33.8, 29.4, 29.4, 29.2, 29.1, 29.1, 28.9, 28.5.

HRMS (ESI): m/z $[M + H]^+$ calcd for $C_{13}H_{25}S_2$: 245.1392; found: 245.1395.

11-vinyldisulfanylundecanoic acid methyl ester 2c

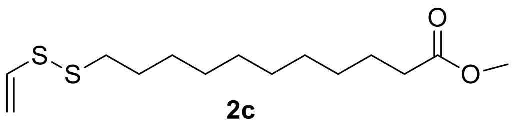

2c

Chromatography: Hexene/DCM 2/1(R_f=0.25), Yield 0.212g, 73% (Table S2 entry 3); 0.256g, 88% (Table S3 entry 2), colorless oil

^{1}H NMR (400 MHz, $CDCl_3$) δ 6.40 (dd, *J* = 16.2, 9.6 Hz, 1H), 5.55 (d, *J* = 16.3 Hz, 1H), 5.36 (d, *J* = 9.6 Hz, 1H), 3.69 (s, 3 H), 2.72 (t, *J* = 7.3 Hz, 2H), 2.32 (t, *J* = 7.5 Hz, 2H), 1.77–1.62 (m, 4H), 1.48–1.20 (m, 12 H).

^{13}C NMR (101 MHz, $CDCl_3$) δ 174.3, 133.8, 113.1, 51.5, 38.2, 34.1, 29.4, 29.3, 29.2, 29.2, 29.1, 29.1, 28.5, 24.9.

HRMS (ESI): m/z $[M + H]^+$ calcd for $C_{14}H_{27}O_2S_2$: 291.1447; found: 291.1452.

11-methoxy-1-vinyldisulfanylundecane 2d

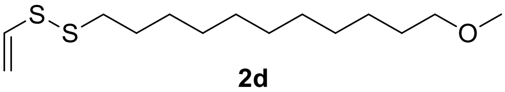

2d

Chromatography: Hexene/DCM 3/1 (R_f=0.44), Yield 0.171g, 62% (Table S2 entry 4), colorless oil

^{1}H NMR (400 MHz, $CDCl_3$) δ 6.40 (dd, *J* =16.2, 9.6 Hz, 1H), 5.56 (d, *J* = 16.2 Hz, 1H), 5.36 (d, *J* = 9.6 Hz, 1H), 3.39 (t, *J* = 6.6 Hz, 2H), 3.35 (s, 3 H), 2.71 (t, *J* =7.4 Hz, 2H), 1.45–1.19 (m, 18 H).

^{13}C NMR (101 MHz, $CDCl_3$) δ 133.8, 113.1, 73.0, 58.5, 38.3, 29.7, 29.5, 29.5, 29.5, 29.2, 29.1, 28.5, 26.1.

HRMS (ESI): m/z $[M + H]^+$ calcd for $C_{14}H_{29}OS_2$: 277.1654; found: 277.1657.

***S*-(11-(vinyldisulfanyl)undec-1-yl) ethanethioate 2e**

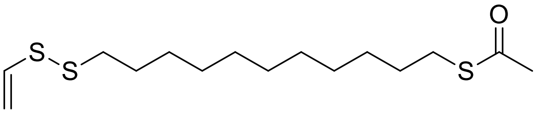

2e

Chromatography: Hexene/Ethyl acetate 3/1 (R_f=0.31), Yield 0.273g, 85% (Table S2 entry 5), yellowish oil

^{1}H NMR (400 MHz, $CDCl_3$) δ 6.40 (dd, *J* =16.2, 9.6 Hz, 1H), 5.55 (d, *J* = 16.2 Hz, 1H), 5.36 (d, *J* = 9.6 Hz, 1H), 2.89 (t, *J* = 7.3 Hz, 2H), 2.72 (t, *J* =7.3 Hz,

2H), 2.34 (s, 3 H), 1.72-1.65 (m, 2H), 1.62–1.54 (m, 2H), 1.46–1.21 (m, 14 H).
^{13}C NMR (101 MHz, $CDCl_3$) δ = 196.1, 133.8, 113.1, 38.2, 30.7, 29.5, 29.5, 29.4, 29.2, 29.1, 29.1, 28.8, 28.5.
HRMS (ESI): m/z $[M + H]^+$ calcd for $C_{15}H_{29}OS_3$: 321.1375; found: 321.1371.

N-(2-vinyldisulfanylethyl)-carbamic acid *tert*-butyl ester 2f

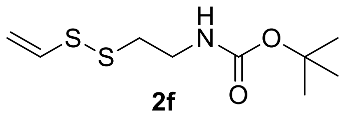

2f

Chromatography: Hexene/DCM 2/1 (R_f=0.35), Yield 0.177g, 75% (Table S2 entry 6), colorless oil

^{1}H NMR (400 MHz, $CDCl_3$) δ 6.40 (dd, *J* = 16.2, 9.5Hz, 1H), 5.58 (d, *J* = 16.2 Hz, 1H), 5.41 (d, *J* = 9.6 Hz, 1H), 4.90 (s, 1 H), 3.45 (dd, *J* =11.6, 5.6 Hz, 2H), 2.83 (t, *J* =6.2 Hz, 2H), 1.47 (s, 9 H).

^{13}C NMR (101 MHz, $CDCl_3$) δ 155.7, 133.1, 114.1, 79.6, 39.1, 37.7, 28.4.
HRMS (ESI): m/z $[M + H]^+$ calcd for $C_9H_{18}NO_2S_2$: 236.0773; found: 236.0772.

4-(2-vinyldisulfanyleth-1-yl)toluene 2g

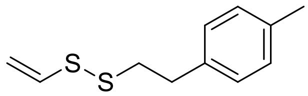

2g

Chromatography: Hexene (R_f=0.55), Yield 0.160g, 76% (Table S2 entry 7), colorless oil

^{1}H NMR (400 MHz, $CDCl_3$) δ 7.16–7.09 (m, 4 H), 6.42 (dd, *J* = 16.2, 9.5 Hz, 1H), 5.57 (d, *J* = 16.3 Hz, 1H), 5.38 (d, *J* = 9.6 Hz, 1H), 2.98–2.93 (m, 4 H), 2.35 (s, 3 H).

^{13}C NMR (101 MHz, $CDCl_3$) δ 133.5, 129.2, 129.2, 128.5, 128.5, 113.5, 39.3, 35.1, 21.1.
HRMS (ESI): m/z $[M + H]^+$ calcd for $C_{11}H_{15}S_2$: 211.0610; found: 211.0614.

3-(2-vinyldisulfanyleth-1-yl)-1*H*-indole 2h

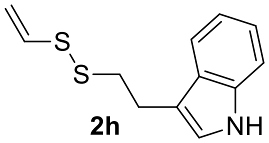

2h

Chromatography: Hexene/DCM 2/1 (R_f=0.27), Yield 0.177g, 75% (Table S2 entry 8), colorless oil, stored at -10°C

^{1}H NMR (400 MHz, $CDCl_3$) δ 8.02 (s, 1H), 7.62 (d, *J* = 7.9 Hz, 1H), 7.39 (dd, *J* = 8.1, 0.8 Hz, 1H), 7.26–7.20 (m, 1H), 7.15 (ddd, *J* = 8.0, 7.1, 1.0 Hz, 1H), 7.08 (d, *J* = 2.3 Hz, 1H), 6.45 (dd, *J* = 16.2, 9.6 Hz, 1H), 5.60 (d, *J* = 16.2 Hz, 1H), 5.39 (d, *J* = 9.6 Hz, 1H), 3.19 (ddd, *J* = 8.1, 3.7, 2.4 Hz, 2H), 3.12–3.01 (m, 2H).

^{13}C NMR (101 MHz, $CDCl_3$) δ 133.7, 122.2, 121.8, 119.4, 118.7, 114.3, 113.5, 111.2, 38.5, 25.3.

HRMS (ESI): m/z $[M + H]^+$ calcd for $C_{12}H_{14}NS_2$: 236.0562; found: 236.0566.

4-(2-vinyldisulfanyleth-1-yl)-trifluoromethylbenzene 2i

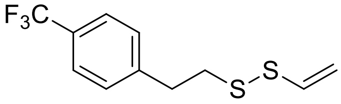

2i

Chromatography: Hexene/DCM 5/1 (R_f=0.33), Yield 0.172g, 65% (Table S2 entry 9), colorless oil

^{1}H NMR (400 MHz, $CDCl_3$) δ 7.58 (d, *J* = 8.0 Hz, 2H), 7.34 (d, *J* = 8.0 Hz, 2H), 6.40 (dd, *J* = 16.2, 9.5 Hz, 1H), 5.57 (d, *J* = 16.3 Hz, 1H), 5.40 (d, *J* = 9.5 Hz, 1H), 3.13 – 3.03 (m, 2H), 3.03 – 2.86 (m, 2H).

^{13}C NMR (101 MHz, $CDCl_3$) δ 143.9, 133.2, 129.0, 128.7, 125.5 (q, *J* = 3.8 Hz), 113.9, 39.5, 35.3.

HRMS (ESI): m/z $[M + H]^+$ calcd for $C_{11}H_{12}F_3S_2$: 265.0327; found: 265.0329.

4-(2-vinyldisulfanyleth-1-yl)-fluorobenzene 2j

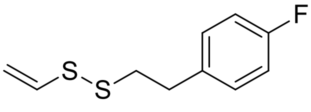

2j

Chromatography: Hexene/DCM 4/1 (R_f=0.44), Yield 0.193g, 90% (Table S3 entry 3), colorless oil,

^{1}H NMR (400 MHz, $CDCl_3$) δ 7.20-7.10 (m, 2H), 7.08-6.95 (m, 2H), 6.41 (dd, *J* = 16.2, 9.6 Hz, 1H), 5.56 (d, *J* = 16.2 Hz, 1H), 5.39 (d, *J* = 9.5 Hz, 1H), 3.02–2.90 (m, 4H).

^{13}C NMR (101 MHz, $CDCl_3$) δ 163.2, 143.8, 135.7(d, *J* = 3.4 Hz), 130.0(d, *J*=8.0Hz), 115.3(d, *J*= 21.1 Hz), 113.6, 39.2, 34.7.
HRMS (ESI): m/z $[M + H]^+$calcd for $C_{10}H_{12}FS_2$: 215.0359; found: 215.0364.

4-vinyldisulfanyltoluene 2k

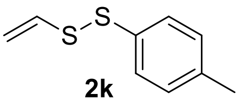

2k

Chromatography: Hexene (R_f=0.6), Yield 0.175g, 96% (Table S3 entry 4), yellowish oil,

^{1}H NMR (400 MHz, $CDCl_3$) δ 7.41 (d, *J* = 8.2 Hz, 2H), 7.15 (d, *J* = 8.0 Hz, 2H), 6.48 (dd, *J* = 16.2, 9.5 Hz, 1H), 5.57 (d, *J* = 16.2 Hz, 1H), 5.39 (d, *J* = 9.5 Hz, 1H), 2.36 (s, 3H).

^{13}C NMR (101 MHz, $CDCl_3$) δ 137.6, 133.1, 132.9, 129.8, 128.7, 114.2, 21.1.

HRMS (ESI): m/z $[M + H]^+$ calcd for $C_9H_{11}S_2$: 183.0297; found: 183.0303.

2-(vinyldisulfanylmethyl)naphthalene 2l

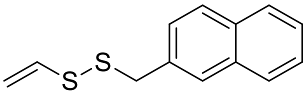

2l

Chromatography: Hexene/Toluene 5/1 (R_f=0.5), Yield 0.214g, 92% (Table S3 entry 5), yellowish oil,

^{1}H NMR (400 MHz, $CDCl_3$) δ 7.87–7.82 (m, 3H), 7.75 (s, 1H), 7.55–7.45 (m, 3H), 6.27 (dd, *J* = 26.3, 16.8 Hz, 1H), 5.51 (d, *J* = 16.3 Hz, 1H), 5.28 (d, *J* = 9.5 Hz, 1H), 4.09 (s, 2H).

^{13}C NMR (101 MHz, $CDCl_3$) δ 134.2, 133.3, 133.2, 132.7, 128.4, 127.8, 127.2, 126.1, 113.8, 42.8.

HRMS (ESI): m/z $[M + H]^+$ calcd for $C_{13}H_{13}S_2$: 233.0453; found: 233.0456.

4-(vinyldisulfanylmethyl)nitrobenzene 2m

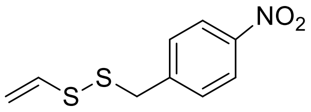

2m

Chromatography: Hexane/Toluene 2/1(R_f=0.3), Yield 0.222g, 80% (Table S3 entry 6), yellow oil,

^{1}H NMR (400 MHz, $CDCl_3$) δ 8.21 (d, *J* = 8.7 Hz, 2H), 7.48 (d, *J* = 8.7 Hz, 2H), 6.20 (dd, *J* = 16.3, 9.5 Hz, 1H), 5.46 (d, *J* = 16.3 Hz, 1H), 5.30 (d, *J* = 9.5 Hz, 1H), 3.96 (s, 2H).

^{13}C NMR (101 MHz, $CDCl_3$) δ 147.3, 144.6, 132.6, 130.2, 123.8, 114.5, 41.3.

HRMS (ESI): m/z $[M + H]^+$ calcd for $C_9H_{10}NO_2S_2$: 228.0147; found: 228.0152.

4-(vinyldisulfanylmethyl)methoxybenzene 2n

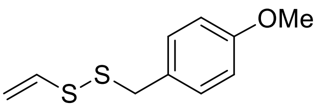

2n

Chromatography: Hexane/DCM 3/1 (R_f= 0.36), Yield 0.185g, 87% (Table S3 entry 7), colorless oil,

^{1}H NMR (400 MHz, $CDCl_3$) δ 7.25 (d, *J* = 8.6 Hz, 2H), 6.88 (d, *J* = 8.6 Hz, 2H), 6.26 (dd, *J* = 16.3, 9.5 Hz, 1H), 5.50 (d, *J* = 16.3 Hz, 1H), 5.32 (d, *J* = 9.5 Hz, 1H), 3.89 (s, 2H), 3.83 (s, 3H).

^{13}C NMR (101 MHz, $CDCl_3$) δ 159.1, 133.2, 130.5, 128.8, 114.0, 113.5, 55.3, 41.9.

HRMS (ESI): m/z $[M + H]^+$ calcd for $C_{10}H_{13}OS_2$: 213.0402; found: 213.0408.

4-(vinyldisulfanylmethyl)benzonitrile 2o

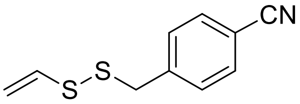

2o

Chromatography: Hexane/DCM 2/1 (R_f= 0.45), Yield 0.185g, 89% Table S3 entry 8), colorless oil,

^{1}H NMR (400 MHz, $CDCl_3$) δ 7.64 (d, *J* = 8.3 Hz, 2H), 7.43 (d, *J* = 8.3 Hz, 2H), 6.19 (dd, *J* = 16.3, 9.5 Hz, 1H), 5.45 (d, *J* = 16.3 Hz, 1H), 5.30 (d, *J* = 9.5 Hz, 1H), 3.92 (s, 2H).

^{13}C NMR (101 MHz, $CDCl_3$) δ 137.8, 127.9, 127.5, 125.4, 114.0, 109.6, 106.6, 36.8.

HRMS (ESI): m/z $[M + H]^+$ calcd for $C_{10}H_{10}NS_2$: 208.0249; found: 208.0246.

Vinyl-benzyl disulfide 2r

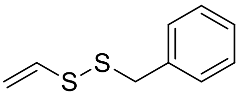

2r

Chromatography: Hexane (R_f=0.6), Yield 0.179g, 98% (Table S3 entry 9), yellowish oil,

^{1}H NMR (400 MHz, $CDCl_3$) δ 7.44– .27 (m, 5H), 6.25 (dd, *J* = 16.3, 9.5 Hz, 1H), 5.49 (d, *J* = 16.3 Hz, 1H), 5.31 (d, *J* = 9.5 Hz, 1H), 3.93 (s, 2H).

^{13}C NMR (101 MHz, $CDCl_3$) δ 136.8, 133.1, 129.4, 128.6, 127.6, 113.7, 42.5.

HRMS (ESI): m/z $[M + H]^+$ calcd for $C_9H_{11}S_2$: 183.0297; found: 183.0302.

11-(vinyldisulfanyl)undecanoic acid 2s

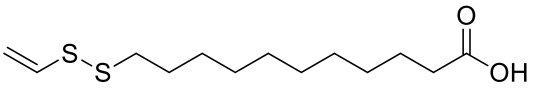

2s

Chromatography: DCM/Ethyl acetate 9/1 (R_f=0.3), Yield 0.232g 84% (Table S3 entry 10), colorless oil,

^{1}H NMR (400 MHz, $CDCl_3$) δ 6.40 (dd, *J* = 16.2, 9.6 Hz, 1H), 5.56 (d, *J* = 16.2 Hz, 1H), 5.36 (d, *J* = 9.6 Hz, 1H), 2.70 (t, *J* = 7.4 Hz,, 2H), 2.37 (t, *J* = 7.5 Hz, 2H), 1.75–1.60 (m, 4H), 1.45–1.26 (m, 12H).

^{13}C NMR (101 MHz, $CDCl_3$) δ 179.5, 133.8, 113.1, 38.2, 29.4, 29.3, 29.2, 29.2, 29.1, 29.0, 28.5, 24.7.

HRMS (ESI): m/z $[M + H]^+$ calcd for $C_{13}H_{25}O_2S_2$: 277.1290; found: 277.1286.

11-(vinyldisulfanyl)-undecan-1-ol 2t

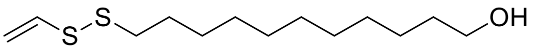

2t

Chromatography: DCM (R_f=0.27), Yield 0.239g, 91% (Table S3 entry 11), colorless oil,

^{1}H NMR (400 MHz, $CDCl_3$) δ 6.40 (dd, *J* = 16.2, 9.6 Hz, 1H), 5.56 (d, *J* = 16.2 Hz, 1H), 5.36 (d, *J* = 9.6 Hz, 1H), 3.67 (t, *J* = 6.6 Hz, 2H), 2.72 (t, *J* = 7.3, 2H), 1.72-1.64 (m, 2H), 1.48–1.14 (m, 16H).

^{13}C NMR (101 MHz, $CDCl_3$) δ 133.8, 113.1, 63.1, 38.2, 32.8, 29.6, 29.5, 29.5, 29.4, 29.2, 29.1, 28.5, 25.7.

HRMS (ESI): m/z $[M + H]^+$ calcd for $C_{13}H_{27}OS_2$: 263.1498; found: 263.1503.

Spectral characterization of benzo[b][1,4]thiazine disulfanyl derivatives 7a-r

3-(dodec-1-yldisulfanyl)-6,8-dimethoxy-4-(4-toluenesulfonyl)-3,4-dihydro-2*H*-benzo[1,4]thiazine 7a

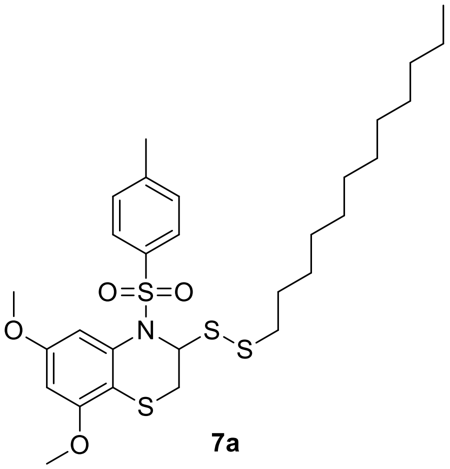

7a

Chromatography: Hexane/DCM 2/1 (R_f=0.32), Yield 0.150g, 50% (Table S4 entry 1), thick yellow oil,

^{1}H NMR (400 MHz, $CDCl_3$) δ 7.52 (d, *J* = 8.3 Hz, 2H), 7.21 (d, *J* = 8.1 Hz, 2H), 7.03 (d, *J* = 2.4 Hz, 1H), 6.37 (d, *J* = 2.4 Hz, 1H), 5.89 (t, *J* = 5.2 Hz, 1H), 3.83 (s, 3H), 3.83 (s, 3H), 3.15-2.85 (m, 2H), 2.87–2.74 (m, 2H), 2.40 (s, 3H), 1.71-1.54 (m, 2H), 1.44–1.21 (m, 18H), 0.88 (t, *J* = 6.9 Hz, 3H).

^{13}C NMR (101 MHz, $CDCl_3$) δ 157.8, 156.0, 144.2, 135.9, 133.4, 129.6, 127.4, 109.2, 105.1, 97.4, 65.4, 56.1, 55.6, 39.2, 31.9, 29.7, 29.7, 29.5, 29.4, 22.7, 21.6, 14.1.

IR (ATR): 2922(w), 2851(w), 1578(w), 1455(w), 1434(w), 1308(s), 1284(w), 1228(w), 1185(w), 1060(w), 1039(w), 842(s), 829(s), 812(s), 705(w), 694(s), 644(s) cm^{-1}

HRMS (ESI): m/z $[M + H]^+$ calcd for $C_{29}H_{44}NO_4S_4$: 598.2148; found: 598.2153.

3-(10-methoxycarbonyldec-1-yldisulfanyl)-6,8-dimethoxy-4-(4-toluenesulfonyl)-3,4-dihydro-2*H*-benzo[1,4]thiazine 7c

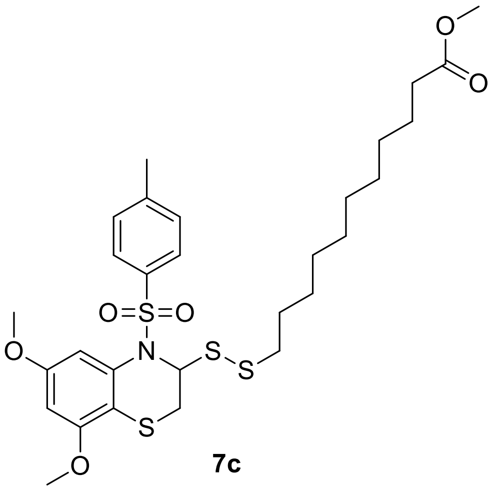

7c

Chromatography: DCM (R_f= 0.45), Yield 0.094g, 30% (Table S4 entry 2), thick yellow oil

^{1}H NMR (400 MHz, $CDCl_3$) δ 7.52 (d, *J* = 8.3 Hz, 2H), 7.21 (d, *J* = 8.1 Hz, 2H), 7.04 (d, *J* = 2.4 Hz, 1H), 6.37 (d, *J* = 2.4 Hz, 1H), 5.89 (t, *J* = 5.2 Hz, 1H), 3.84 (s, 3H), 3.83 (s, 3H), 3.69 (s, 3H), 3.15-2.85 (m, 2H), 2.88–2.74 (m, 2H), 2.40 (s, 3H), 2.33 (t, *J* = 7.5 Hz, 2H), 1.70-1.55 (m, 4H), 1.41–1.25 (m, 12H).

^{13}C NMR (101 MHz, $CDCl_3$) δ 157.8, 156.0, 144.2, 135.9, 129.6, 127.4, 105.1, 97.4, 65.4, 56.1, 55.6, 51.5, 39.2, 34.1, 31.2, 29.4, 29.4, 29.2, 29.2, 29.1, 28.5, 25.0, 21.6.

IR (ATR): 2924(w), 2851(w), 1733(m), 1597(m),1455(m), 1434(m), 1355(m), 1307(m), 1228(m), 1200(vs), 1186(m), 1161(m), 1114(w), 1059(w), 961(w), 829(w), 744(m), 705(s), 694(m) 609(s), 580(m), 566(s), 540(s) cm^{-1}

HRMS (ESI): m/z $[M + H]^+$ calcd for $C_{29}H_{42}NO_6S_4$: 628.1889; found: 628.1901.

3-(4-nitrobenzyldisulfanyl)- 6,8-dimethoxy-4-(4-toluenesulfonyl)-3,4-dihydro-2*H*-benzo[1,4]thiazine 7m

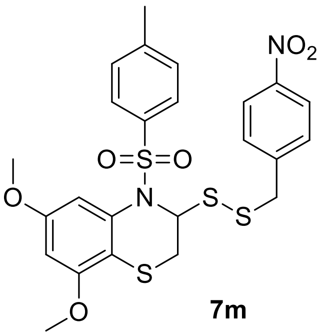

7m

Chromatography: DCM/Toluene 1/2 (R_f= 0.35), Yield 0.082g, 29% (Table S4 entry 3), thick yellow oil

^{1}H NMR (400 MHz, $CDCl_3$) δ 8.19 (d, *J* = 8.7 Hz, 2H), 7.51 – 7.44 (m, 4H), 7.21 (d, *J* = 8.0 Hz, 2H), 7.13 (d, *J* = 2.4 Hz, 1H), 6.39 (d, *J* = 2.4 Hz, 1H), 5.81 (t, *J* = 5.4 Hz, 1H), 4.16 – 4.06 (m, 2H), 3.84 (s, 6H), 2.94 (ddd, *J* = 29.1, 13.3, 5.4 Hz, 2H), 2.41 (s, 3H).

^{13}C NMR (101 MHz, $CDCl_3$) δ 158.1, 156.2, 144.7, 144.5, 135.4, 133.3, 130.3, 129.7, 127.4, 123.8, 105.1, 97.3, 65.5, 56.1, 55.7, 42.3, 31.2, 21.6.

IR (ATR): 2873(w), 2850(w), 1596(m), 1577(m), 1542(m), 1517(m), 1454(w), 1433(w), 1342(vs), 1309(m), 1229(m), 1218(vs), 1201(s), 1186(m), 1095(m), 959(w), 620(w), 579(s), 540(s) cm^{-1}

HRMS (ESI): m/z $[M + H]^+$ calcd for $C_{24}H_{25}N_2O_6S_4$: 565.0590; found: 565.0591.

3-(4-methoxybenzyldisulfanyl)-6,8-dimethoxy-4-(4-toluenesulfonyl)-3,4-dihydro-2*H*-benzo[1,4]thiazine 7n

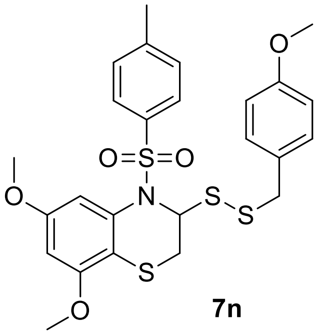

7n

Chromatography: Toluene (R_f=0.21), Yield 0.074g, 27% (Table S4 entry 4), thick yellow oil

^{1}H NMR (400 MHz, $CDCl_3$) δ 7.49 (d, *J* = 8.3 Hz, 2H), 7.28 (d, *J* = 8.6 Hz, 2H), 7.21 (d, *J* = 8.1 Hz, 2H), 7.07 (d, *J* = 2.4 Hz, 1H), 6.88 (t, *J* = 7.3 Hz, 2H), 6.38 (d, *J* = 2.4 Hz, 1H), 5.67 (t, *J* = 5.3 Hz, 1H), 4.13–3.92 (m, 2H), 3.84 (s, 3H), 3.83 (s, 3H), 3.82 (s, 3H), 2.91 (d, *J* = 5.3 Hz, 2H), 2.40 (s, 3H).

^{13}C NMR (101 MHz, $CDCl_3$) δ 159.1, 157.8, 156.0, 144.3, 135.8, 133.3, 130.7, 129.6, 128.9, 127.4, 114.1, 109.3, 105.2, 97.5, 64.9, 56.1, 55.7, 55.3, 43.2, 30.9, 21.6.

IR (ATR): 2998(w), 2836(w), 2361(w), 2331(w), 1596(m), 1577(m), 1510(m), 1454(m), 1355(w), 1303(w), 1248(w), 1200(vs), 1161(s), 1087(s), 1031(s), 958(w), 703(m), 664(m), 540(s) cm^{-1}

HRMS (ESI): m/z $[M + H]^+$ calcd for C25$H_{28}NO_5S_4$: 550.0845; found: 550.0851.

3-(benzyldisulfanyl)-6,8-dimethoxy-4-(4-toluenesulfonyl)-3,4-dihydro-2*H*-benzo[1,4]thiazine 7r

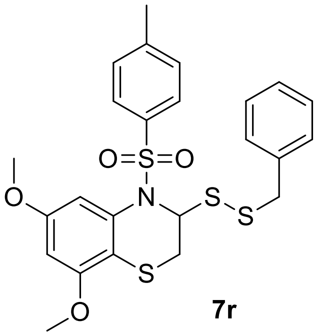

7r

Chromatography: Toluene/Hexane 1/1 (R_f= 0.4), Yield 0.065g, 25% (Table S4 entry 5), thick yellow oil

^{1}H NMR (400 MHz, $CDCl_3$) δ 7.50 (d, *J* = 8.3 Hz, 2H), 7.38–7.30 (m, 5H), 7.21 (d, *J* = 8.0 Hz, 2H), 7.07 (d, *J* = 2.4 Hz, 1H), 6.38 (d, *J* = 2.4 Hz, 1H), 5.70 (t, *J* = 5.3 Hz, 1H), 4.13–3.98 (m, 2H), 3.83 (s, 3H), 3.82 (s, 3H), 2.92 (dt, *J* = 10.9, 5.4 Hz, 2H), 2.41 (s, 3H).

^{13}C NMR (101 MHz, $CDCl_3$) δ 157.9, 156.0, 144.3, 137.0, 135.8, 133.3, 129.6, 129.5, 128.6, 127.4, 109.2, 105.2, 97.5, 65.1, 56.1, 55.7, 43.7, 31.0, 29.7, 21.6.

IR (ATR): 2923(w), 2871(w), 2850(w), 2362(w),1596(m), 1577(m), 1453(m), 1415(m), 1378(m), 1353(m), 1284(vs), 1228(vs), 1200(s), 1185(s), 961(m), 935(m),869(s), 766(s), 579(s) cm^{-1}

HRMS (ESI): m/z $[M + H]^+$ calcd for $C_{24}H_{26}NO_4S_4$: 520.0739; found: 520.0743.

References

[1] a) J. Doroszuk, M. Musiejuk, Ł. Ponikiewski, D. Witt, *Eur. J. Org. Chem.*, **2018**: 6333; b) M. Musiejuk, J. Doroszuk, B. Jędrzejewski, G. Ortiz Nieto, M. Marin Navarro, D. Witt, *Adv. Synth. Catal*. **2020**, *362*, 618-626.
[2] A. Venker, T. Vollgraff, J. Sundermeyer, *Dalton Trans*., **2018**, *47*, 1933-1941.
[3] M. Campo, G. Lamanna, S. Menichetti, *Synlett* **2007**, 2961-2964.

Author Contributions

B. Jędrzejewski, leading investigator (synthesis of starting materials, disulfides and Diels-Alder reactions)
M. Musiejuk, equal investigator (synthesis of starting materials and disulfides)
J. Doroszuk, equal investigator (synthesis of starting materials and disulfides)
D. Witt, principal investigator, (project management, writing of original manuscript)

NMR SPECTRA

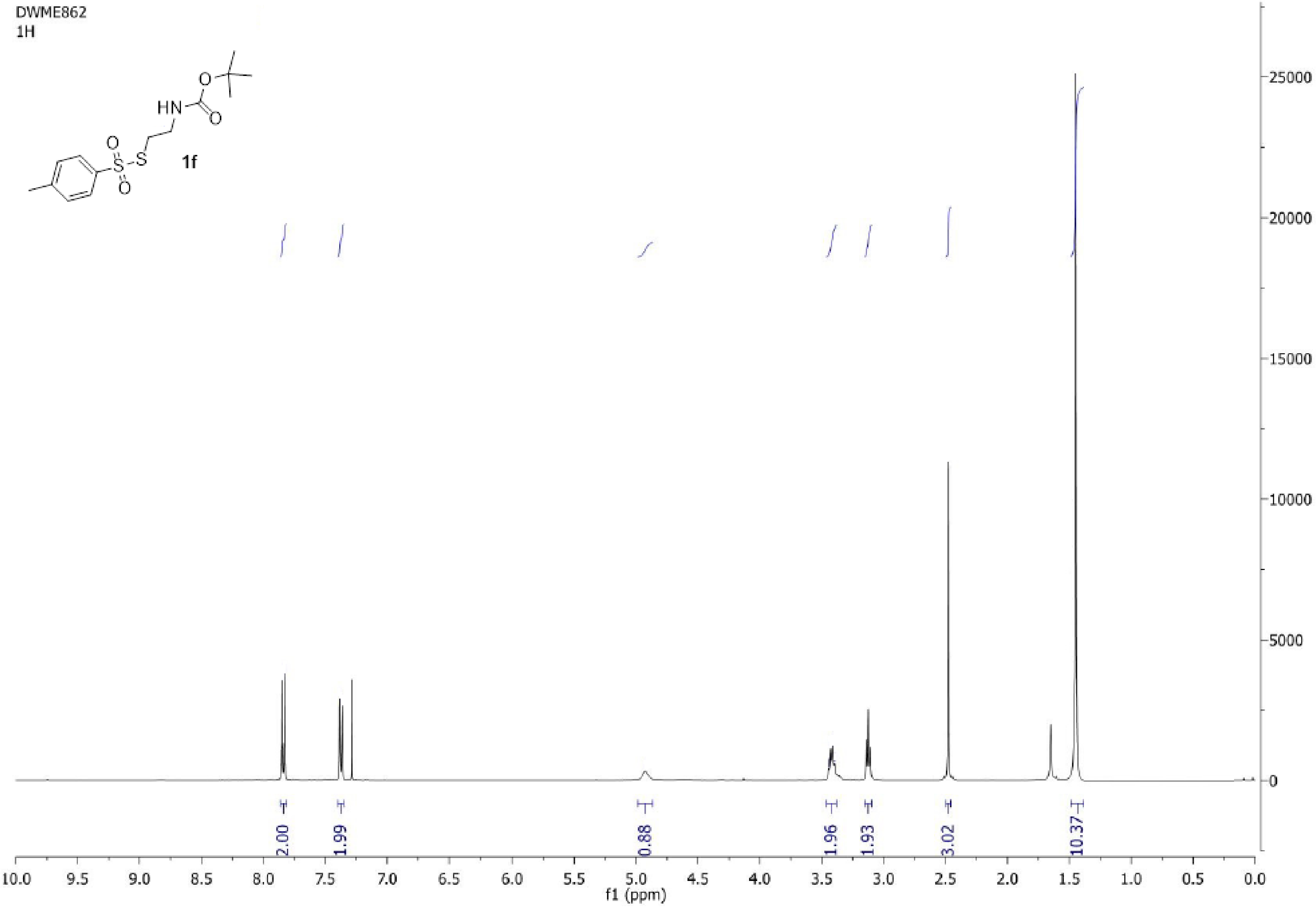
DWME862
1H
HN
O
O
O
S
O
S
1f
2.00
1.99
0.88
1.96
1.93
3.02
10.37
25000
20000
15000
10000
5000
0
10.0 9.5 9.0 8.5 8.0 7.5 7.0 6.5 6.0 5.5 5.0 4.5 4.0 3.5 3.0 2.5 2.0 1.5 1.0 0.5 0.0
f1 (ppm)

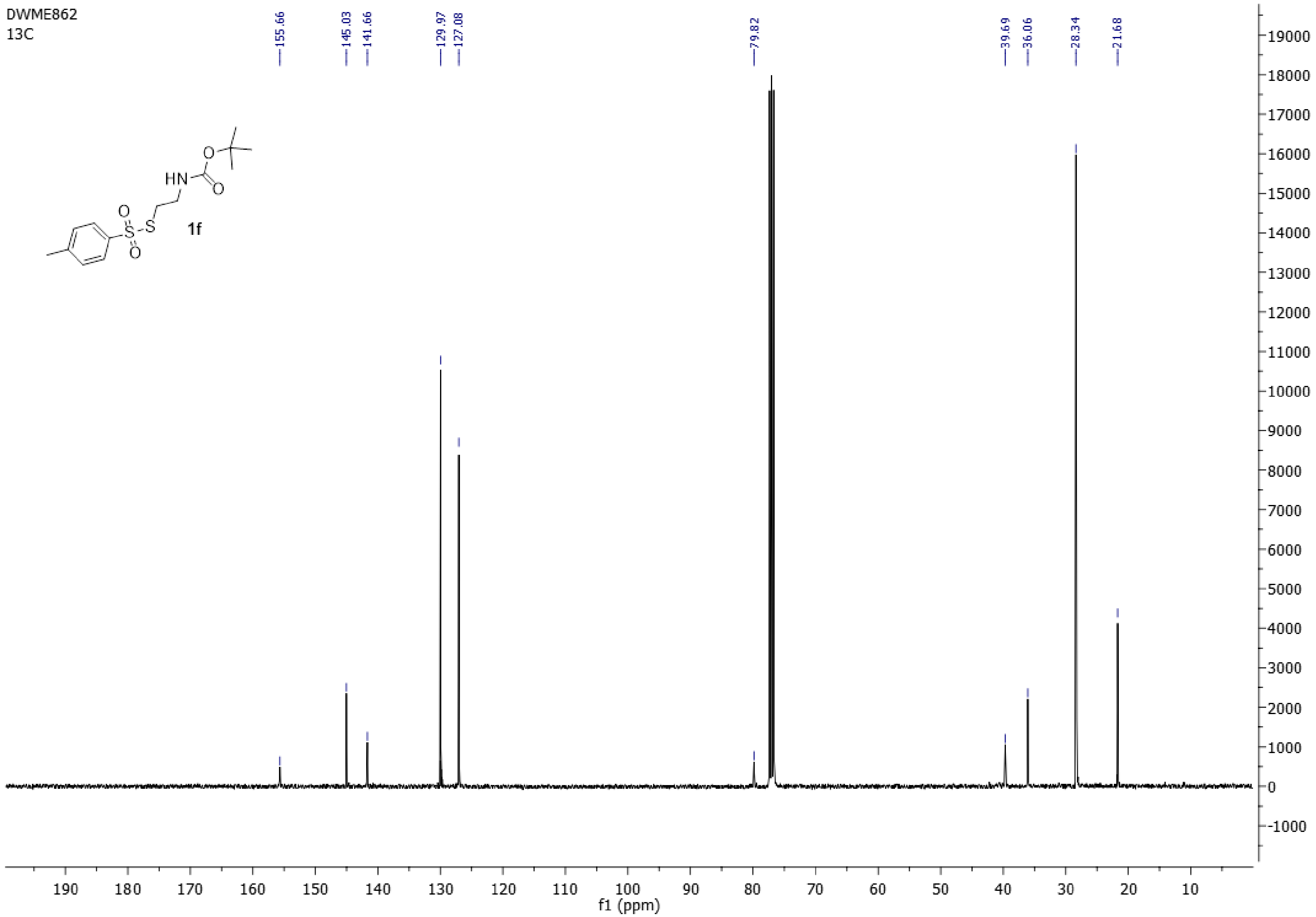
DWME862
13C
155.66
145.03
141.66
129.97
127.08
79.82
39.69
36.06
28.34
21.68
HN
O
O
O
S
S
O
1f
19000
18000
17000
16000
15000
14000
13000
12000
11000
10000
9000
8000
7000
6000
5000
4000
3000
2000
1000
0
-1000
190
180
170
160
150
140
130
120
110
100
90
80
70
60
50
40
30
20
10
f1 (ppm)

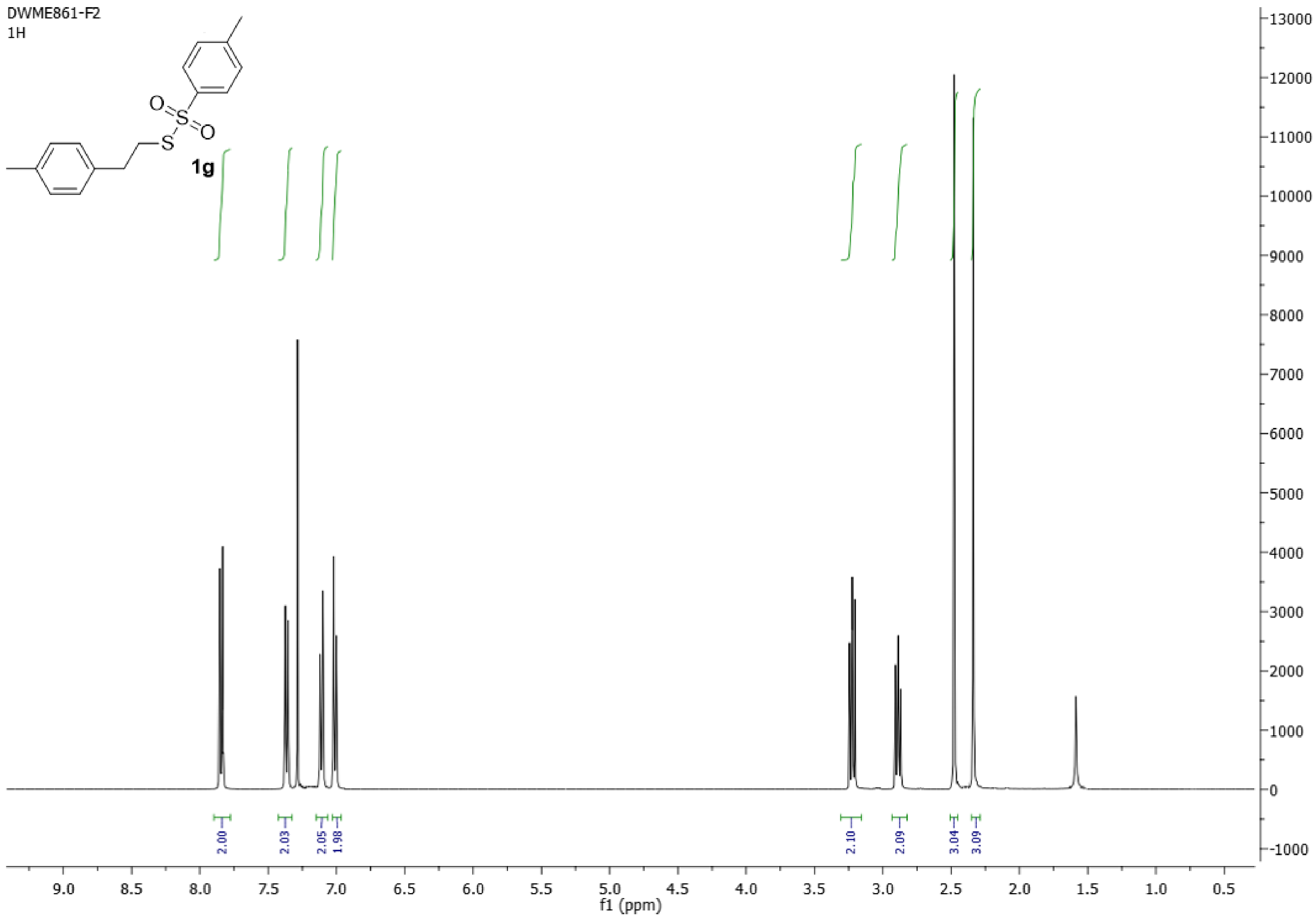
DWME861-F2
1H
O
S
O
S
1g
2.00
2.03
2.05
1.98
2.10
2.09
3.04
3.09
9.0
8.5
8.0
7.5
7.0
6.5
6.0
5.5
5.0
4.5
4.0
3.5
3.0
2.5
2.0
1.5
1.0
0.5
f1 (ppm)
13000
12000
11000
10000
9000
8000
7000
6000
5000
4000
3000
2000
1000
0
-1000

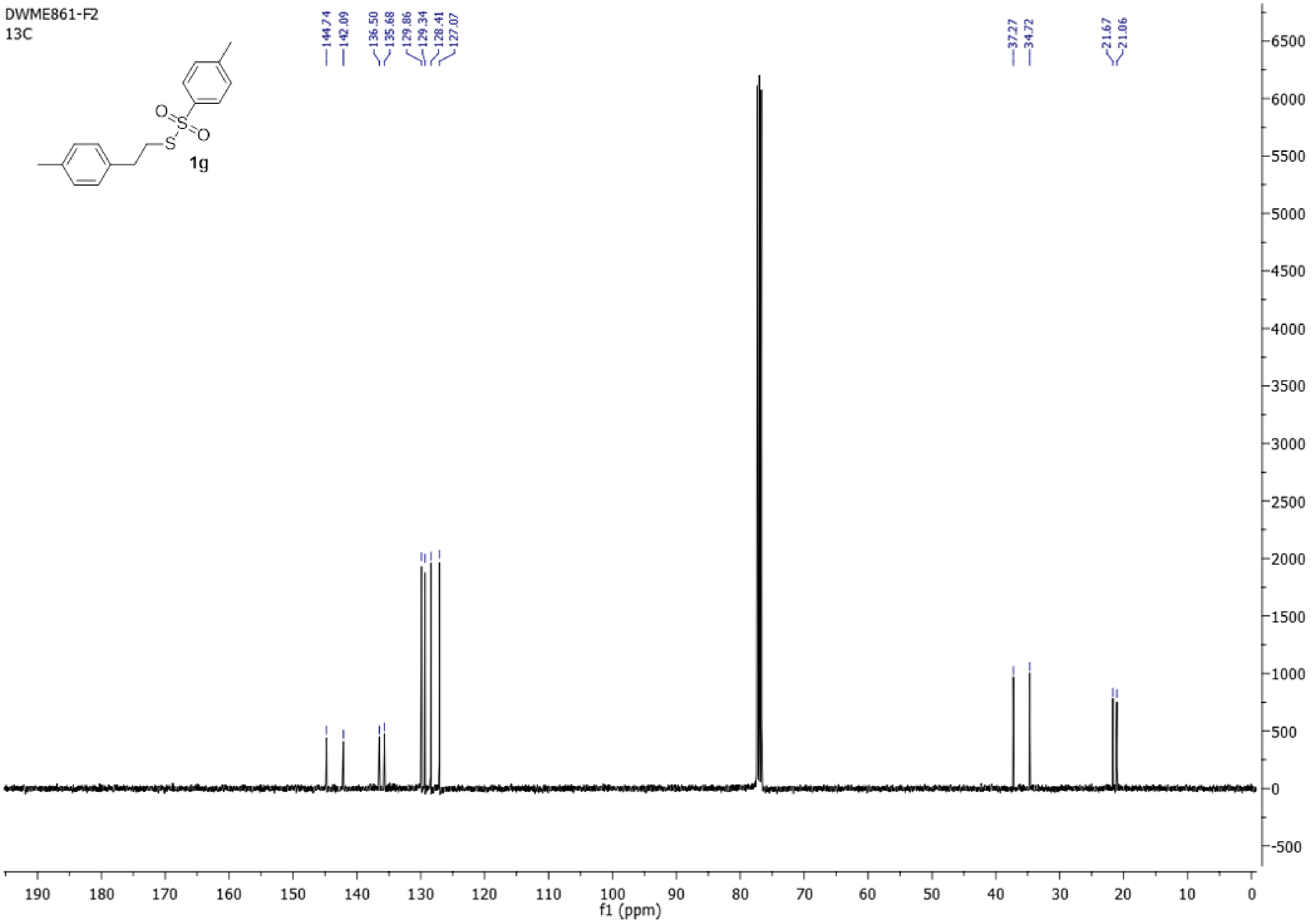
DWME861-F2
13C
1g
144.74
142.09
136.50
135.68
129.86
129.34
128.41
127.07
37.27
34.72
21.67
21.06
f1 (ppm)

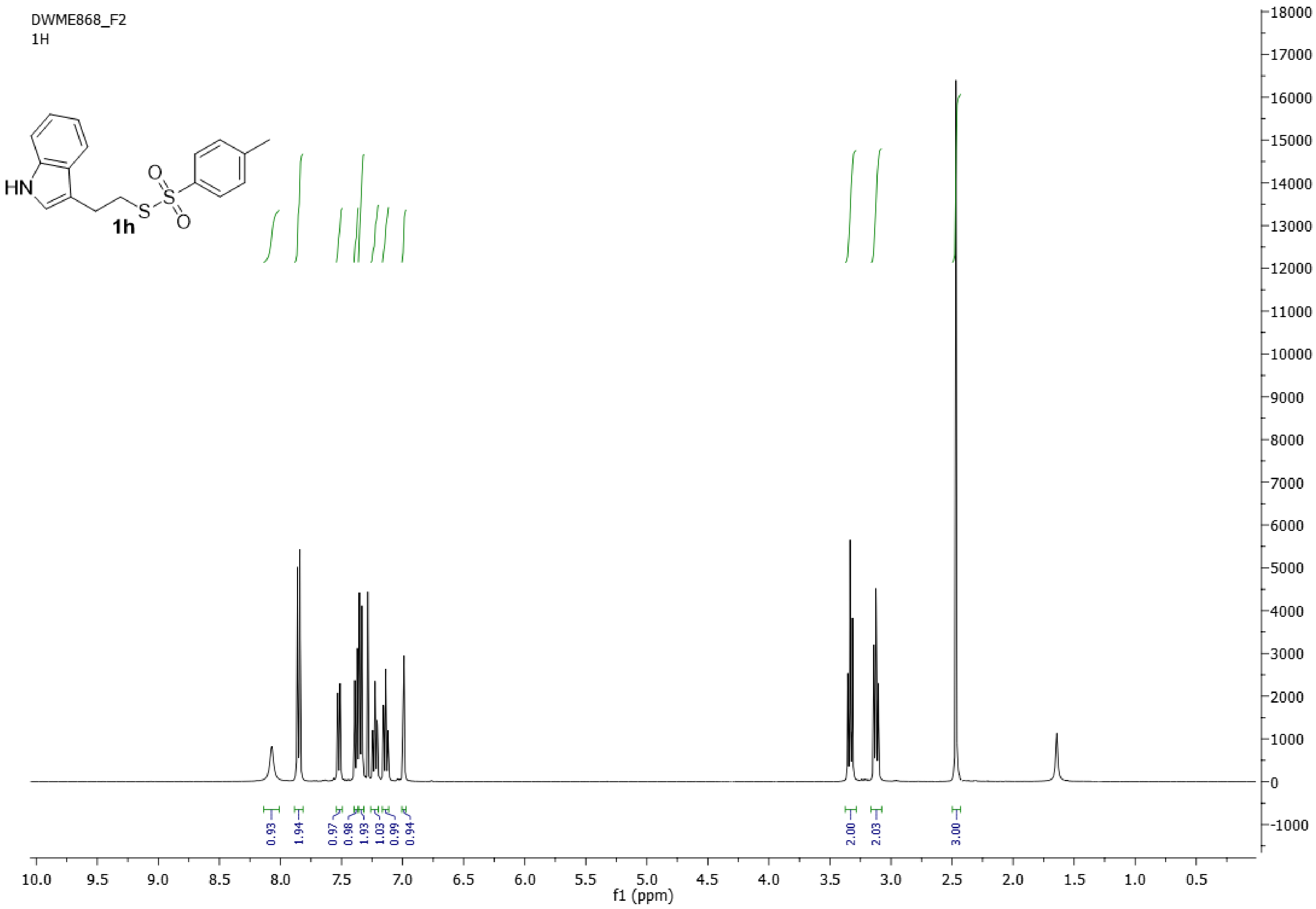
DWME868_F2
1H
HN
S
O
S
O
1h
0.93
1.94
0.97
0.98
1.93
1.03
0.99
0.94
2.00
2.03
3.00
f1 (ppm)

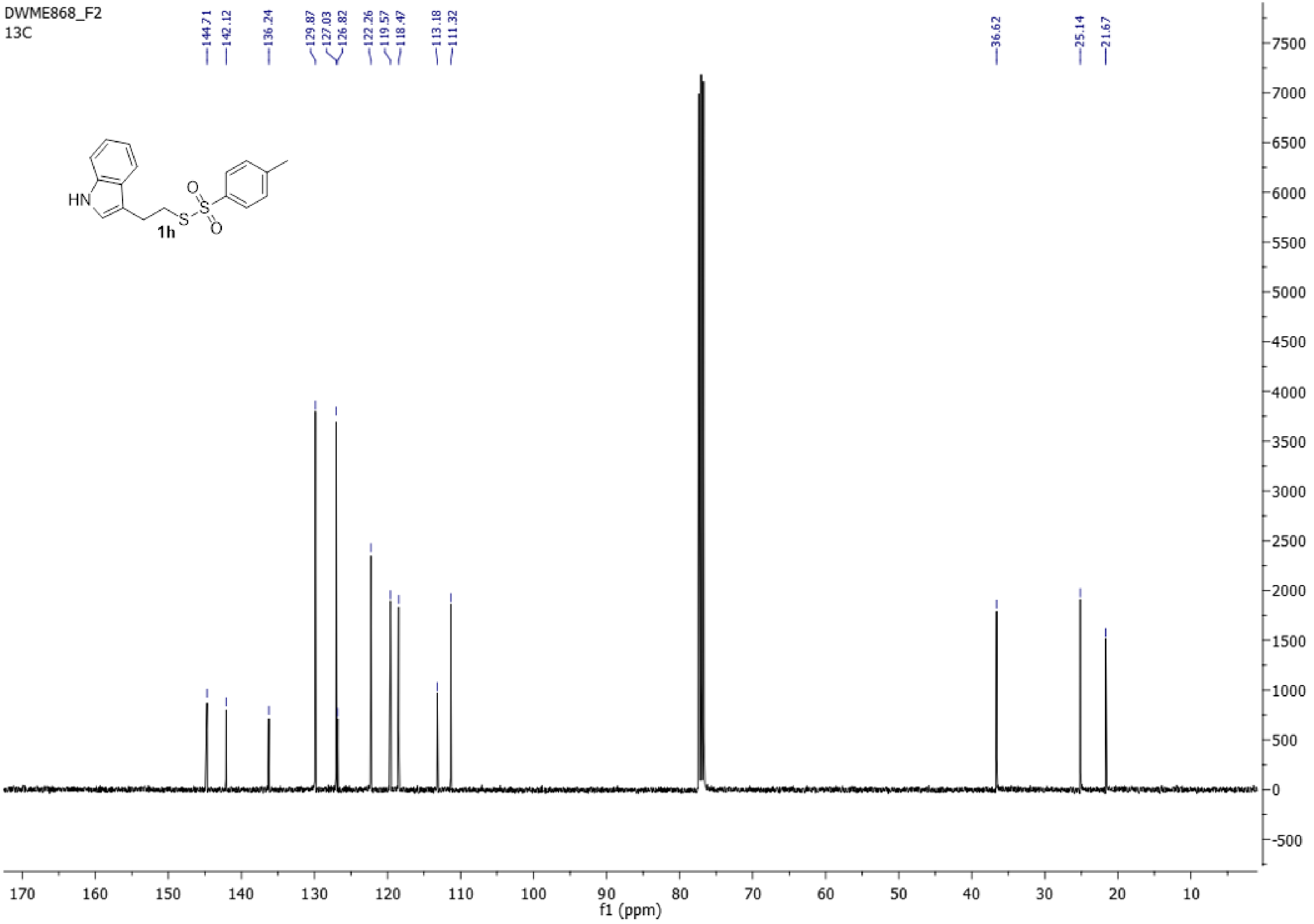
DWME868_F2
13C
144.71
142.12
136.24
129.87
127.03
126.82
122.26
119.57
118.47
113.18
111.32
36.62
25.14
21.67
HN
S
O
1h
f1 (ppm)

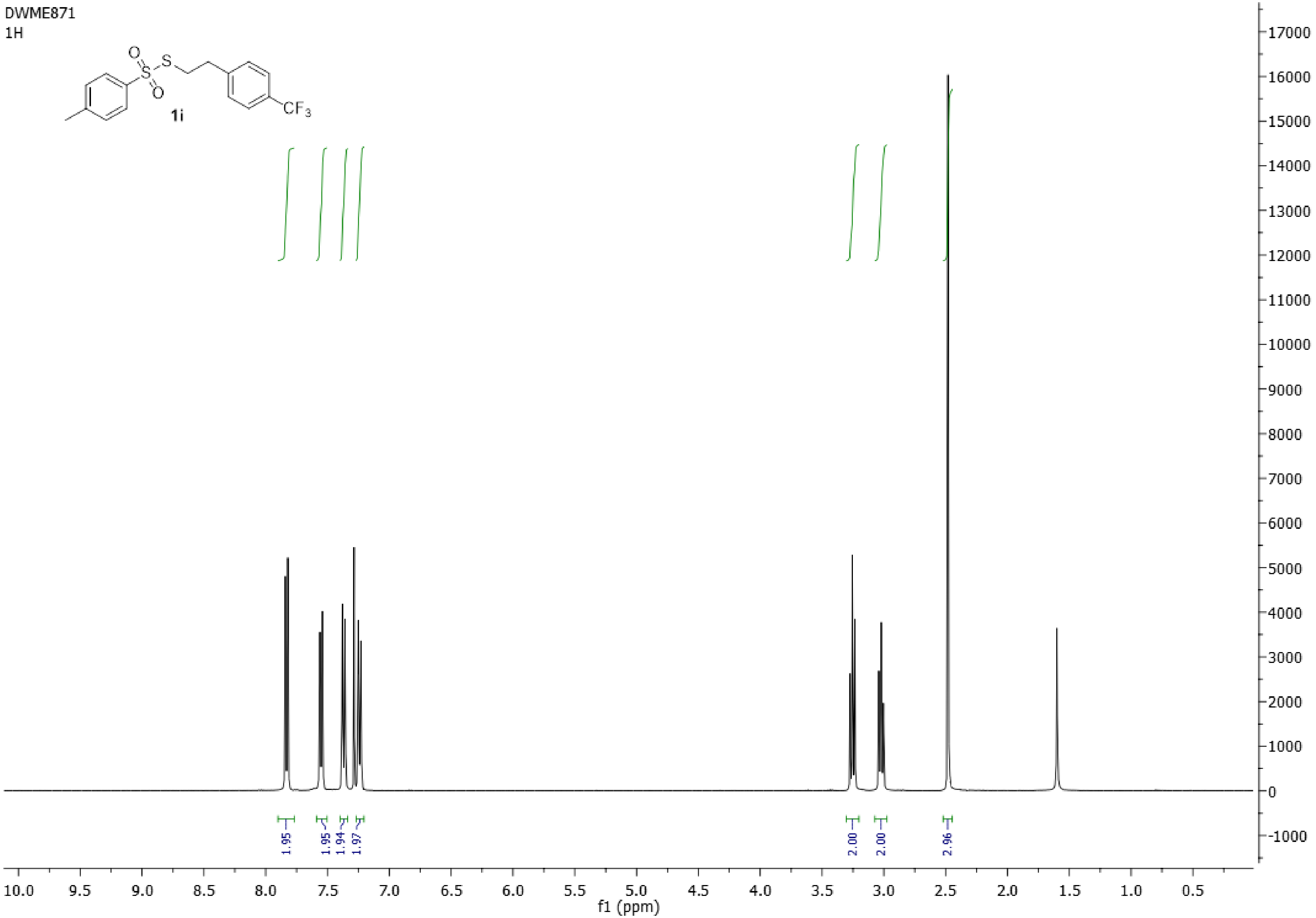
DWME871
1H
1i
CF3
1.95
1.95
1.94
1.97
2.00
2.00
2.96
f1 (ppm)

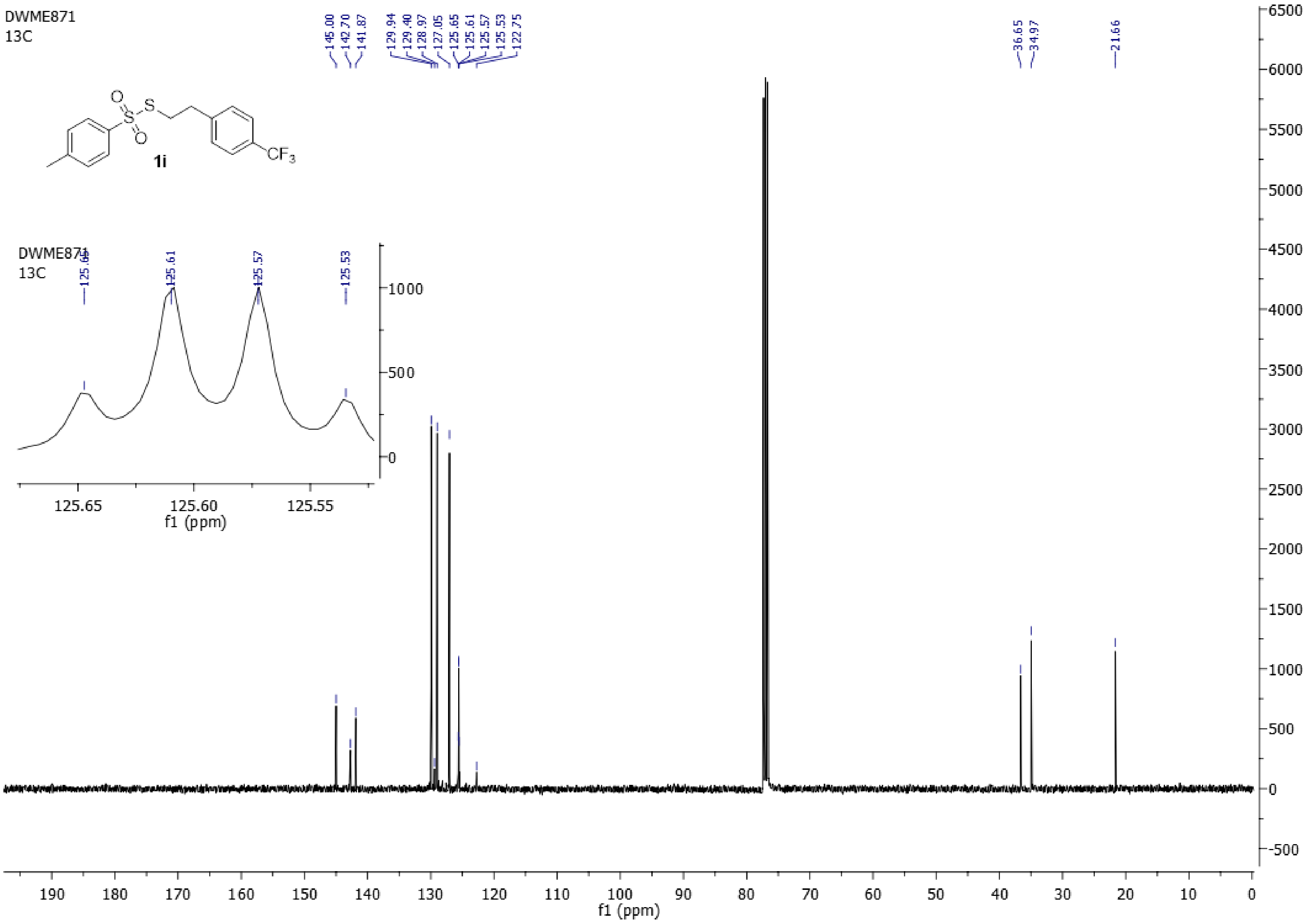

DWME871
13C
145.00
142.70
141.87
129.94
129.40
128.97
127.05
125.65
125.61
125.57
125.53
122.75
36.65
34.97
21.66
1i
CF3
DWME871
13C
125.65
125.60
125.55
f1 (ppm)
f1 (ppm)

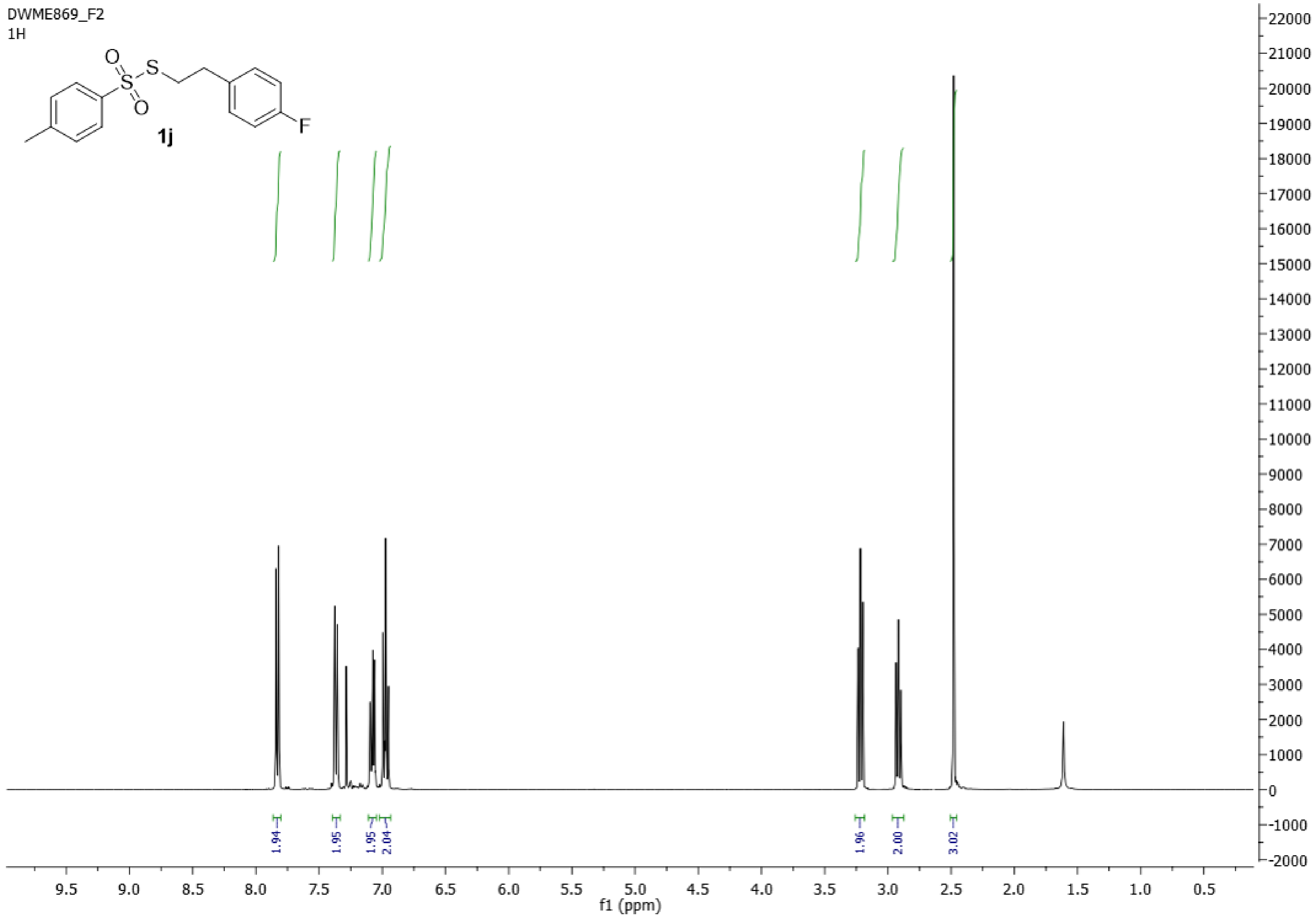
DWME869_F2
1H
1j
1.94
1.95
1.95
2.04
1.96
2.00
3.02
f1 (ppm)

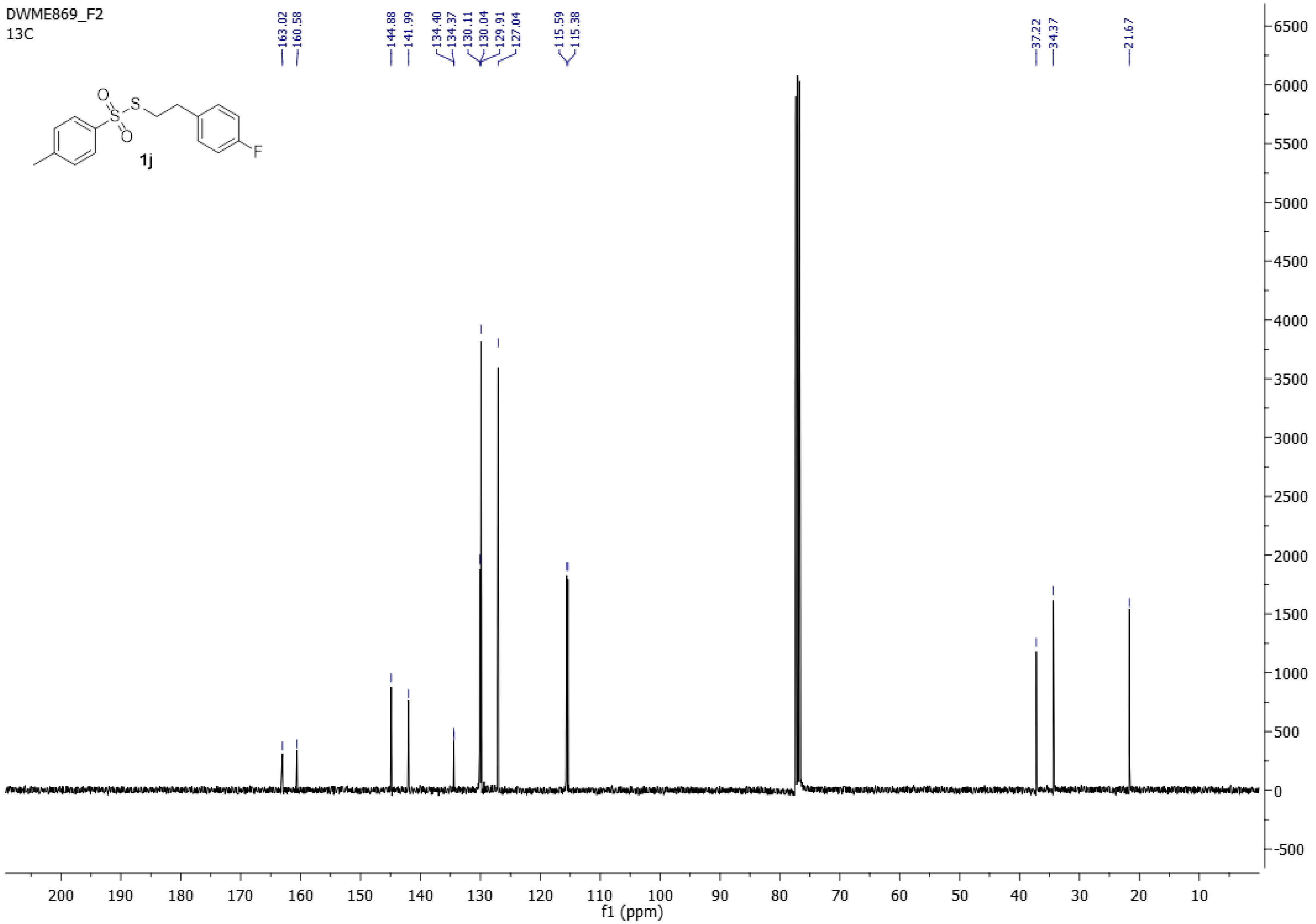
DWME869_F2
13C
1j
F
O
S
O
S
163.02
160.58
144.88
141.99
134.40
134.37
130.11
130.04
129.91
127.04
115.59
115.38
37.22
34.37
21.67
f1 (ppm)

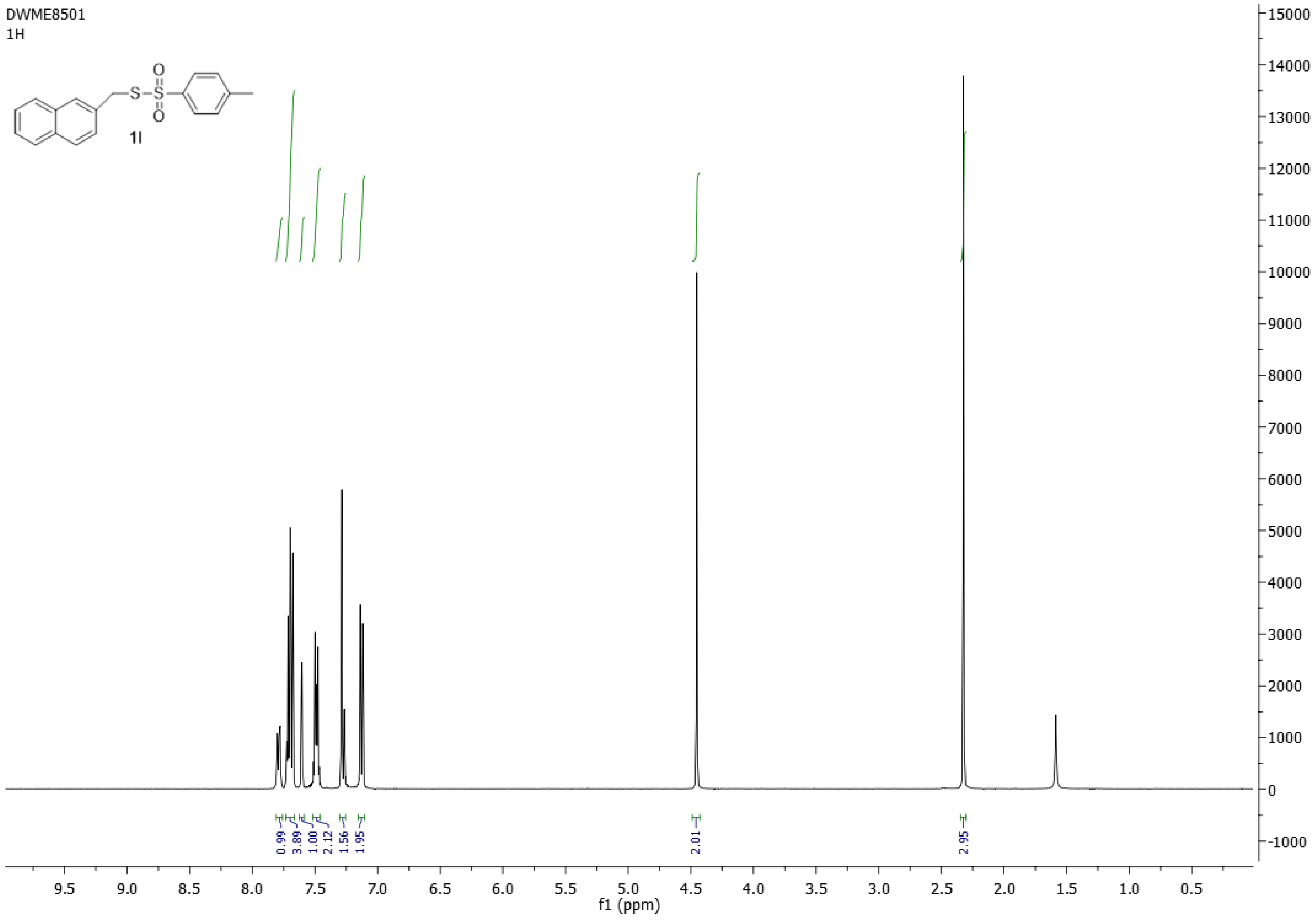
DWME8501
1H
1l
0.99
3.89
1.00
2.12
1.56
1.95
2.01
2.95
f1 (ppm)

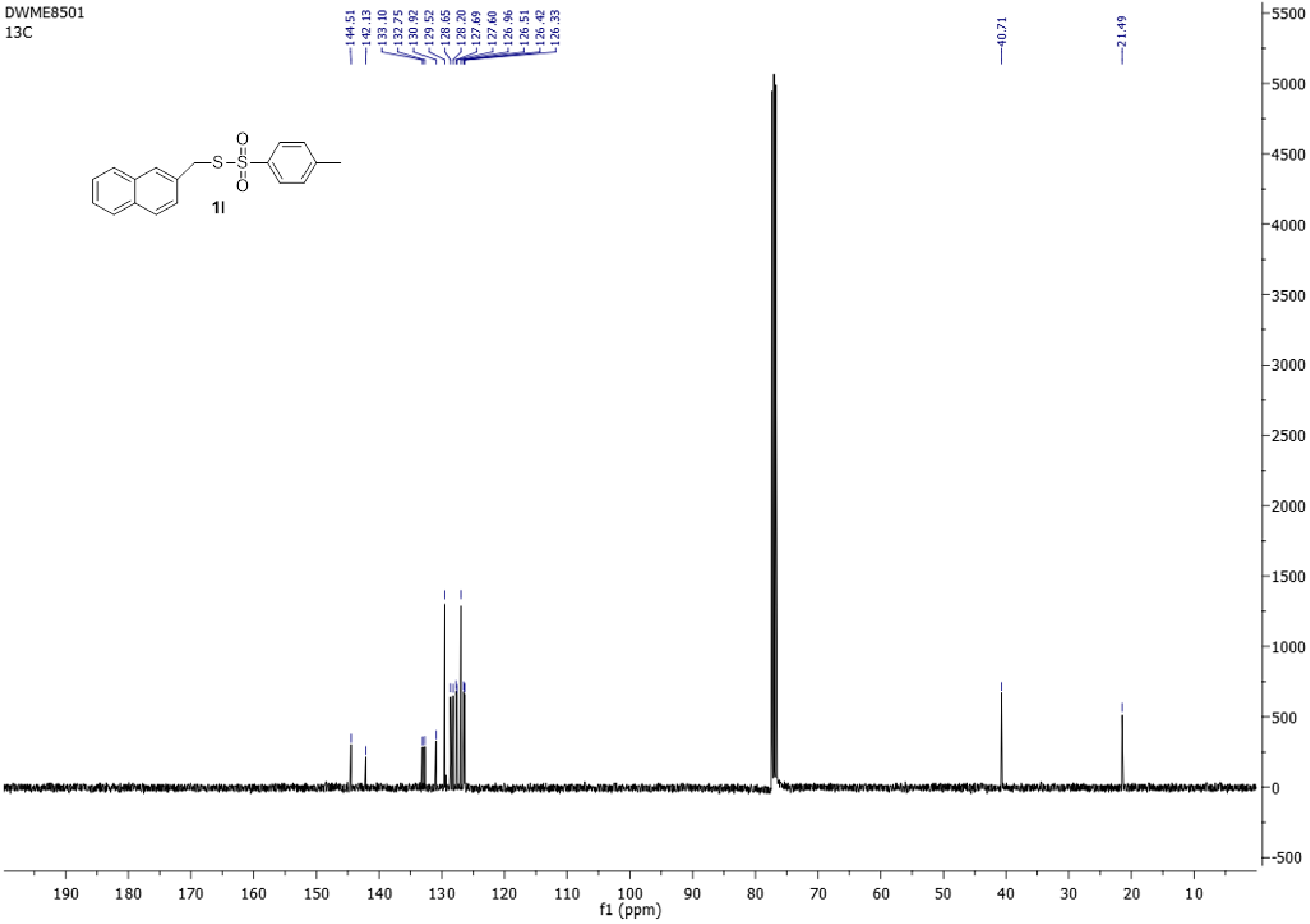
DWME8501
13C
144.51
142.13
133.10
132.75
130.92
129.52
128.65
128.20
127.69
127.60
126.96
126.51
126.42
126.33
40.71
21.49
1l
f1 (ppm)

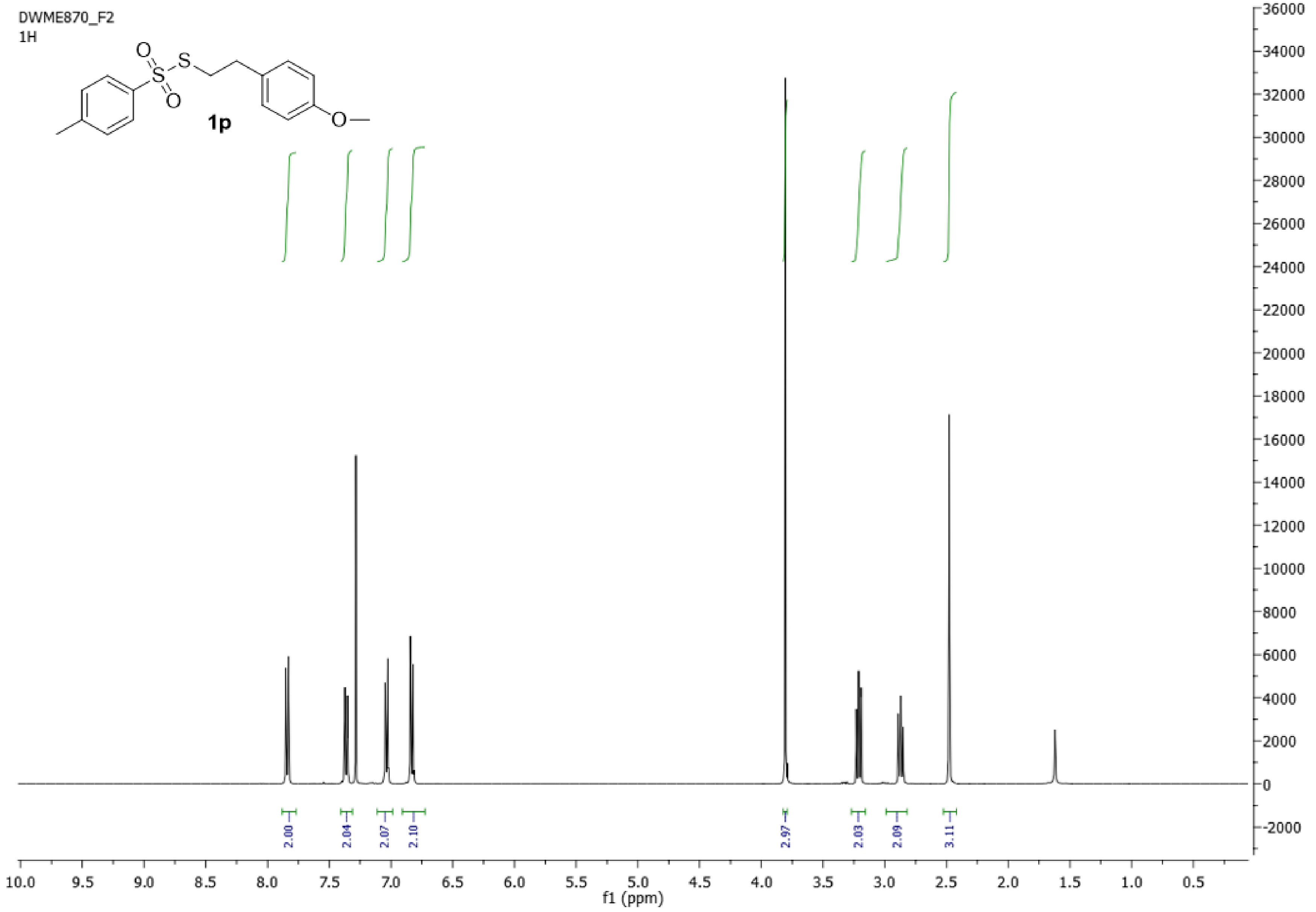
DWME870_F2
1H
1p
2.00
2.04
2.07
2.10
2.97
2.03
2.09
3.11
f1 (ppm)

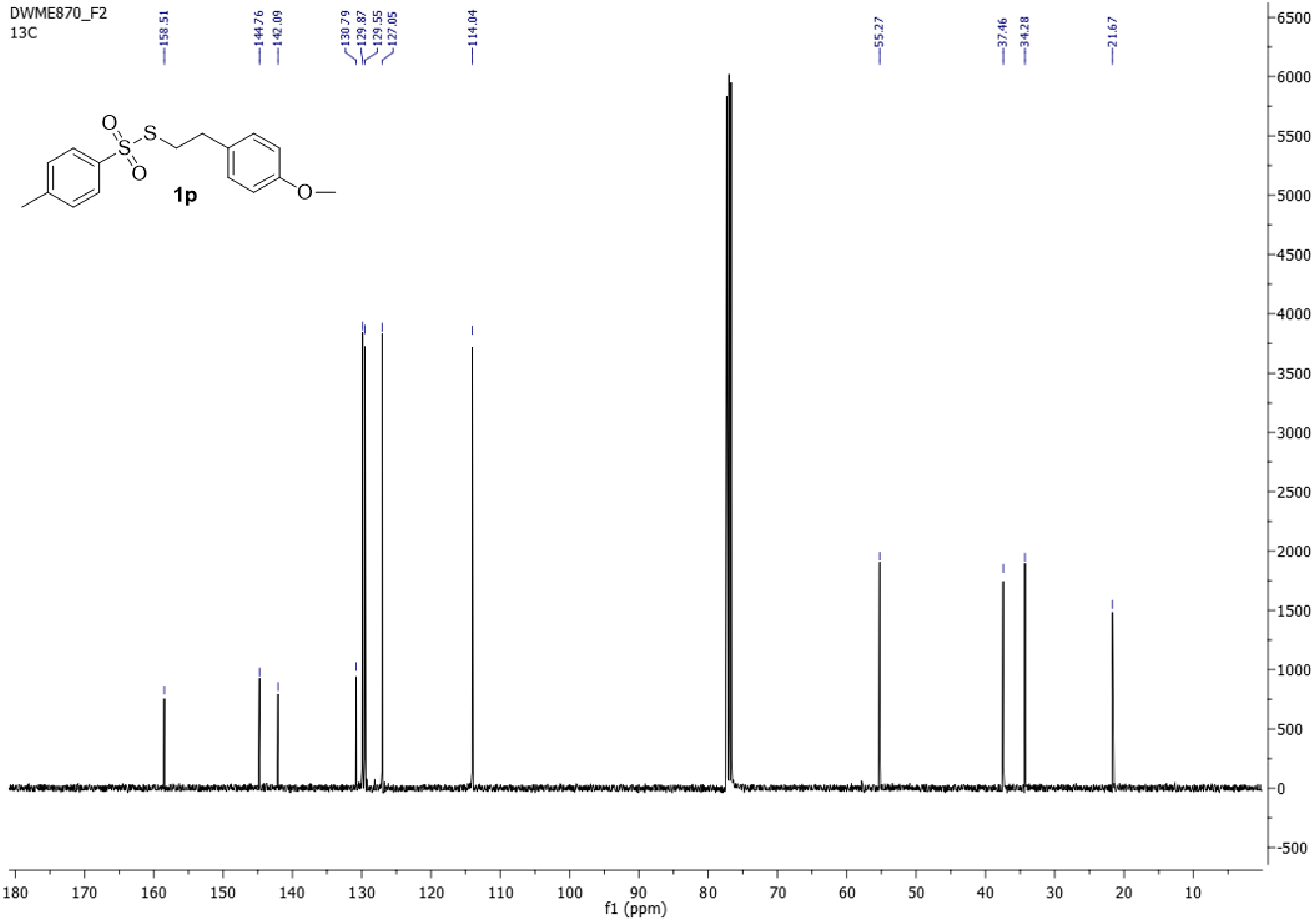
DWME870_F2
13C
158.51
144.76
142.09
130.79
129.87
129.55
127.05
114.04
55.27
37.46
34.28
21.67
1p
f1 (ppm)

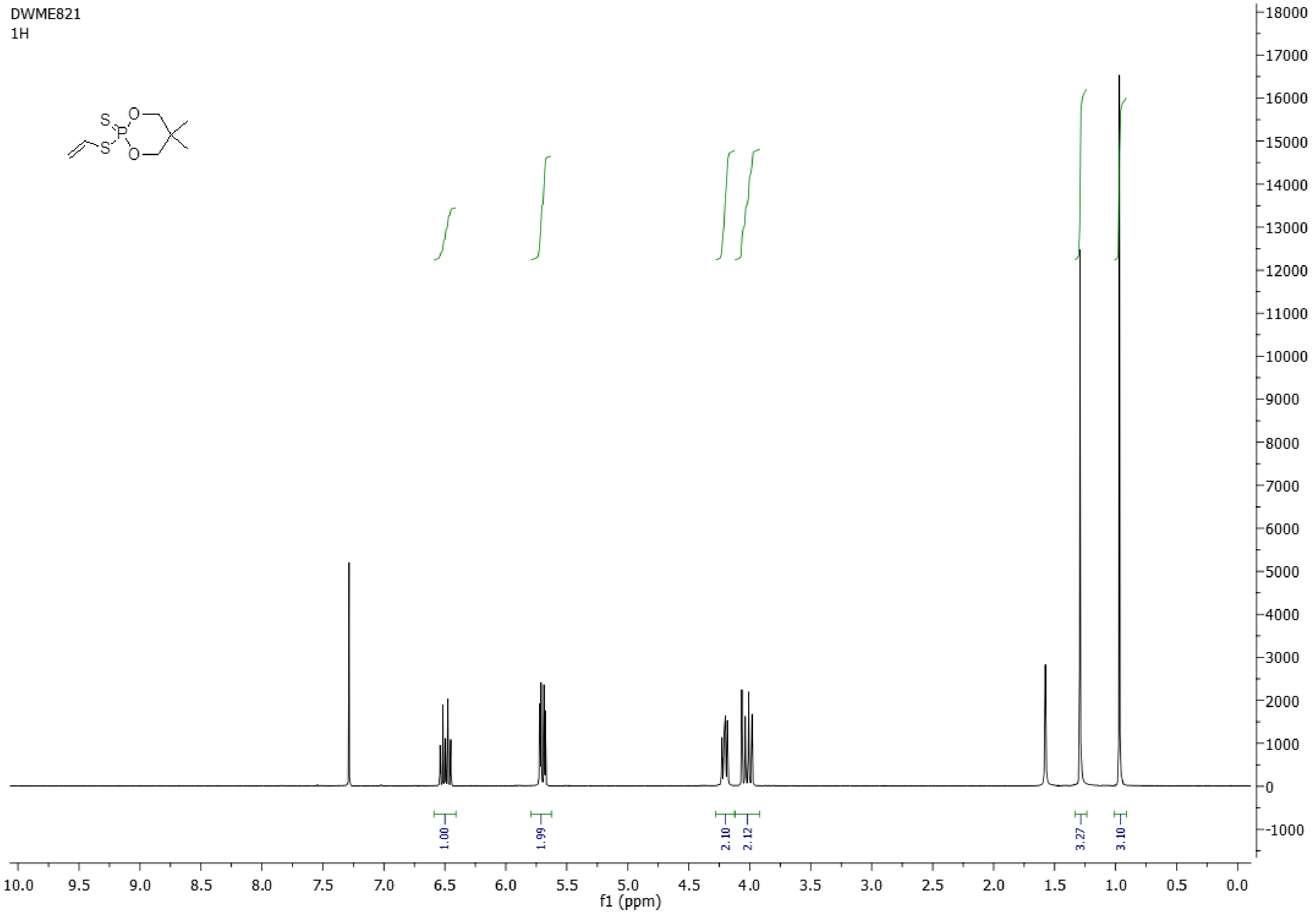
DWME821
1H
1.00
1.99
2.10
2.12
3.27
3.10
f1 (ppm)

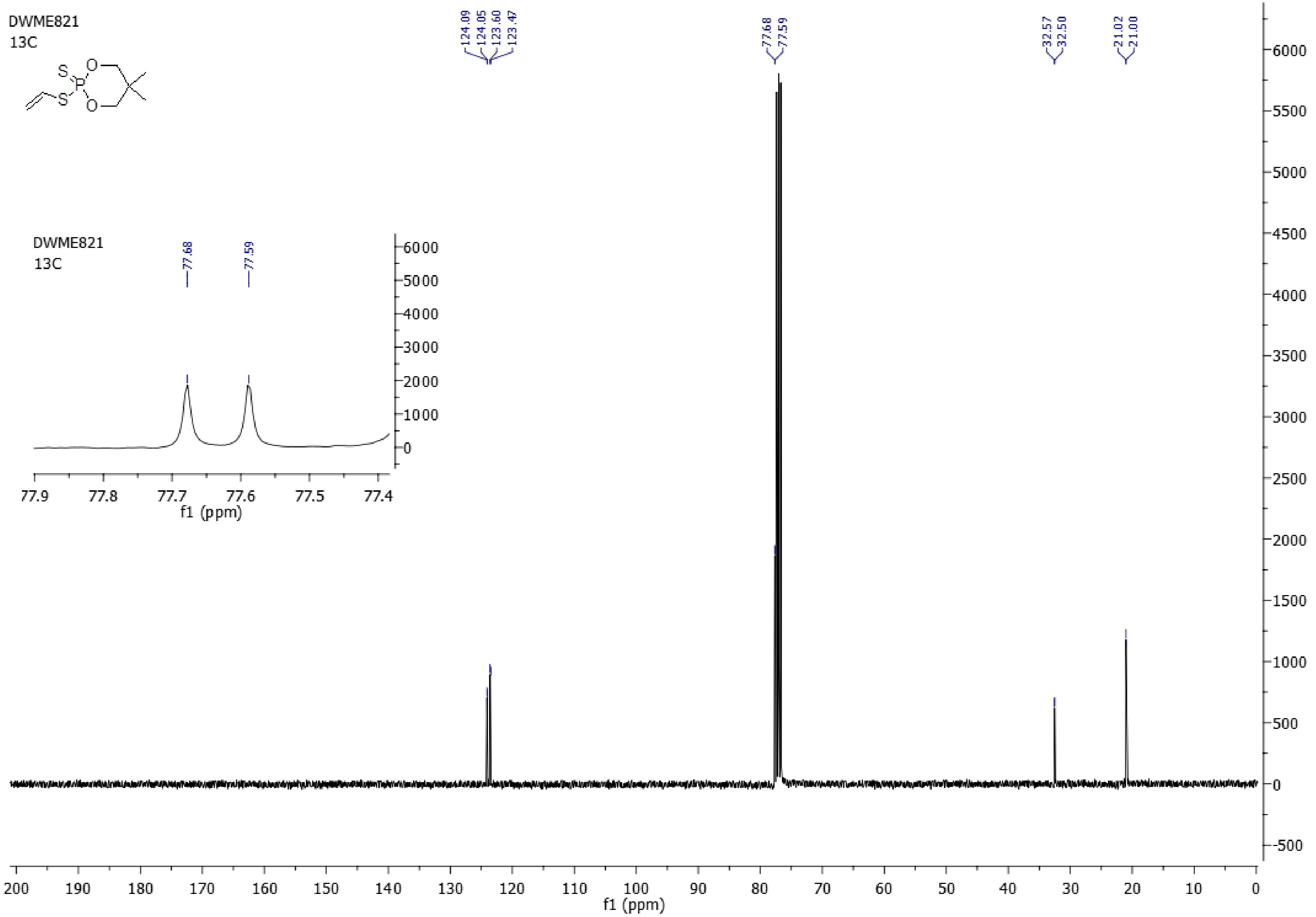

DWME821
13C
S
O
P
S
O
124.09
124.05
123.60
123.47
77.68
77.59
32.57
32.50
21.02
21.00
DWME821
13C
77.68
77.59
77.9
77.8
77.7
77.6
77.5
77.4
f1 (ppm)
6000
5000
4000
3000
2000
1000
0
6000
5500
5000
4500
4000
3500
3000
2500
2000
1500
1000
500
0
-500
200
190
180
170
160
150
140
130
120
110
100
90
80
70
60
50
40
30
20
10
0
f1 (ppm)

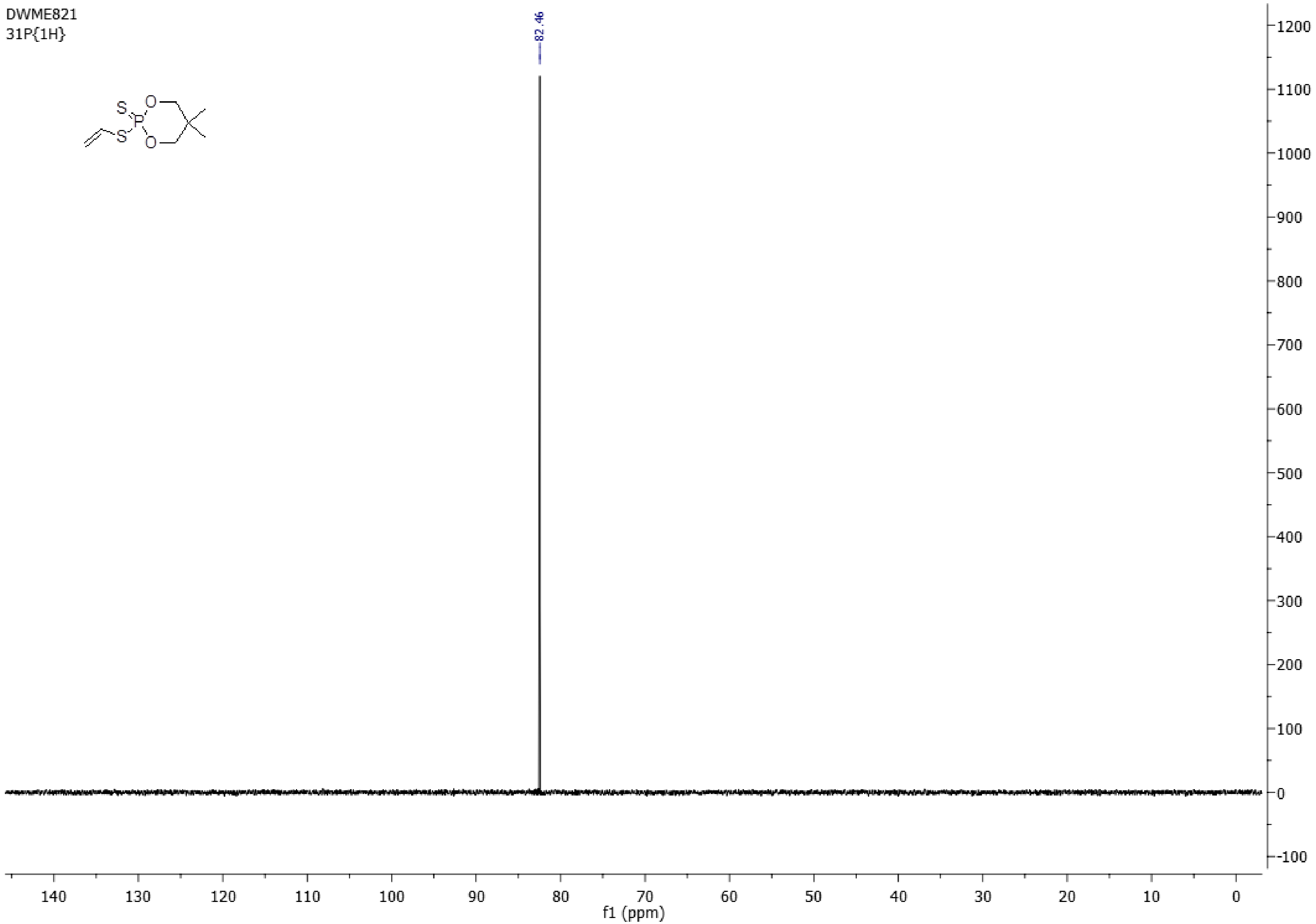

DWME821
31P{1H}
82.46
S
O
P
S
O
f1 (ppm)

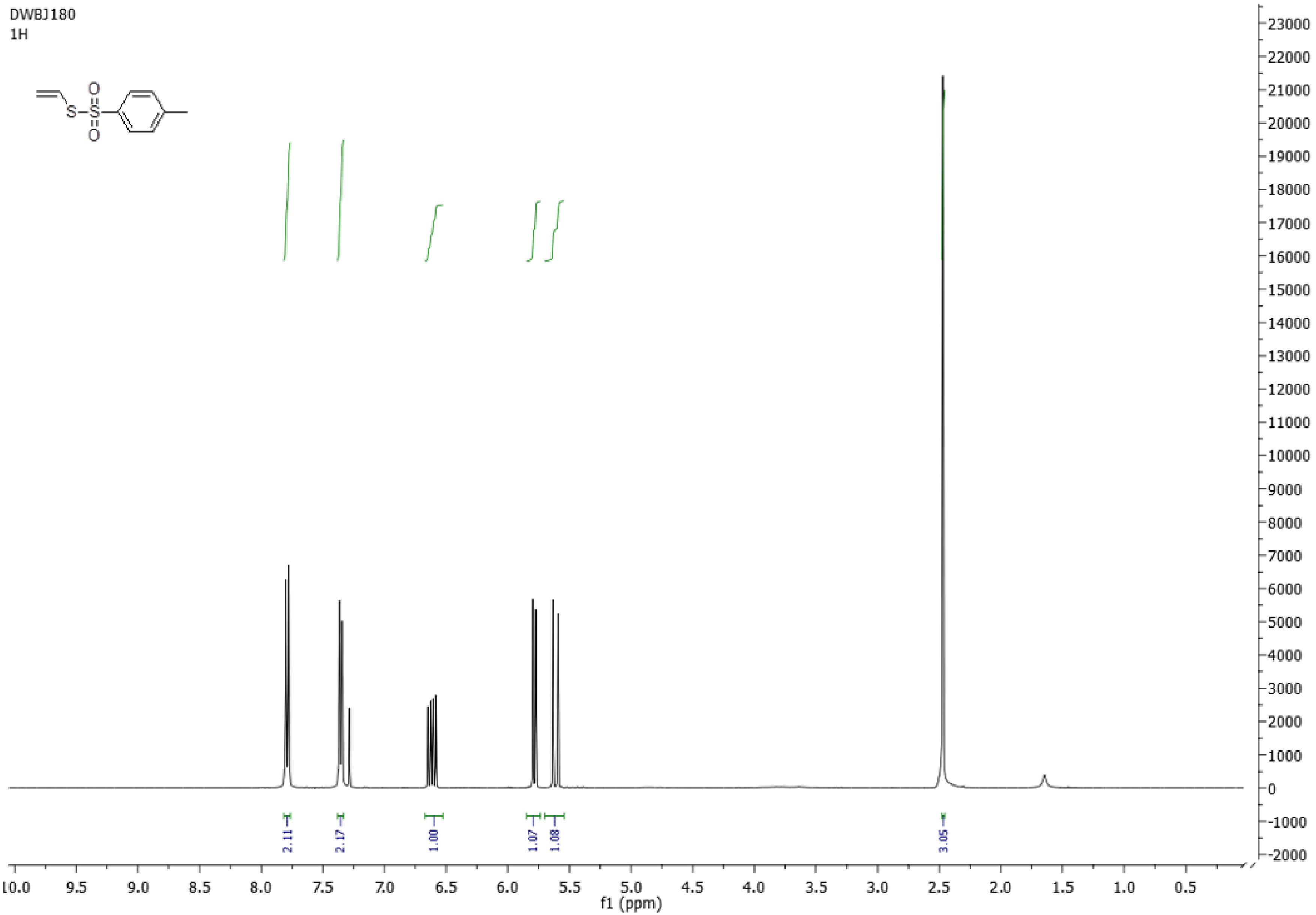
DWBJ180
1H
O
S
S
O
2.11
2.17
1.00
1.07
1.08
3.05
10.0 9.5 9.0 8.5 8.0 7.5 7.0 6.5 6.0 5.5 5.0 4.5 4.0 3.5 3.0 2.5 2.0 1.5 1.0 0.5
f1 (ppm)
23000
22000
21000
20000
19000
18000
17000
16000
15000
14000
13000
12000
11000
10000
9000
8000
7000
6000
5000
4000
3000
2000
1000
0
-1000
-2000

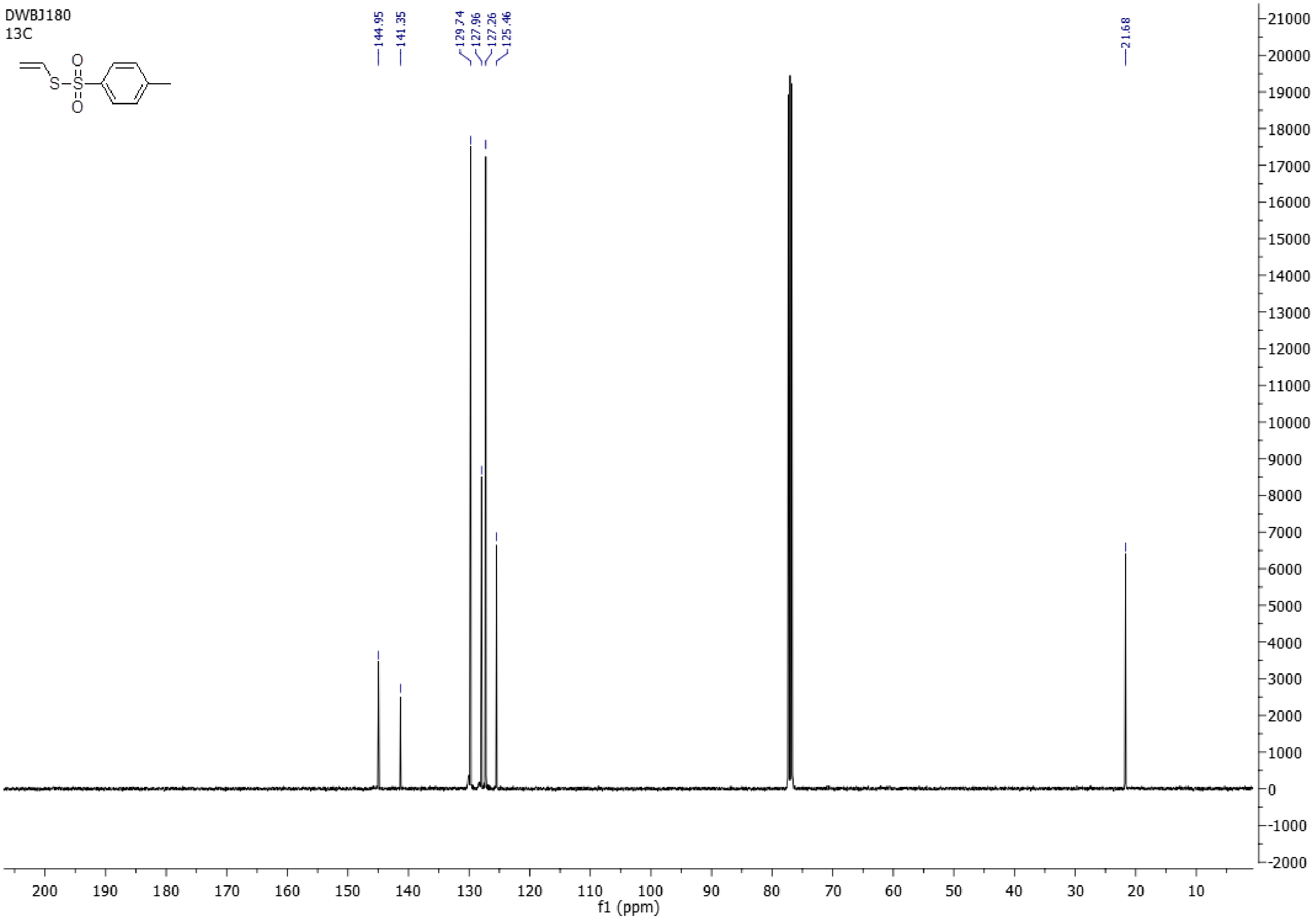
DWBJ180
13C
144.95
141.35
129.74
127.96
127.26
125.46
21.68
f1 (ppm)

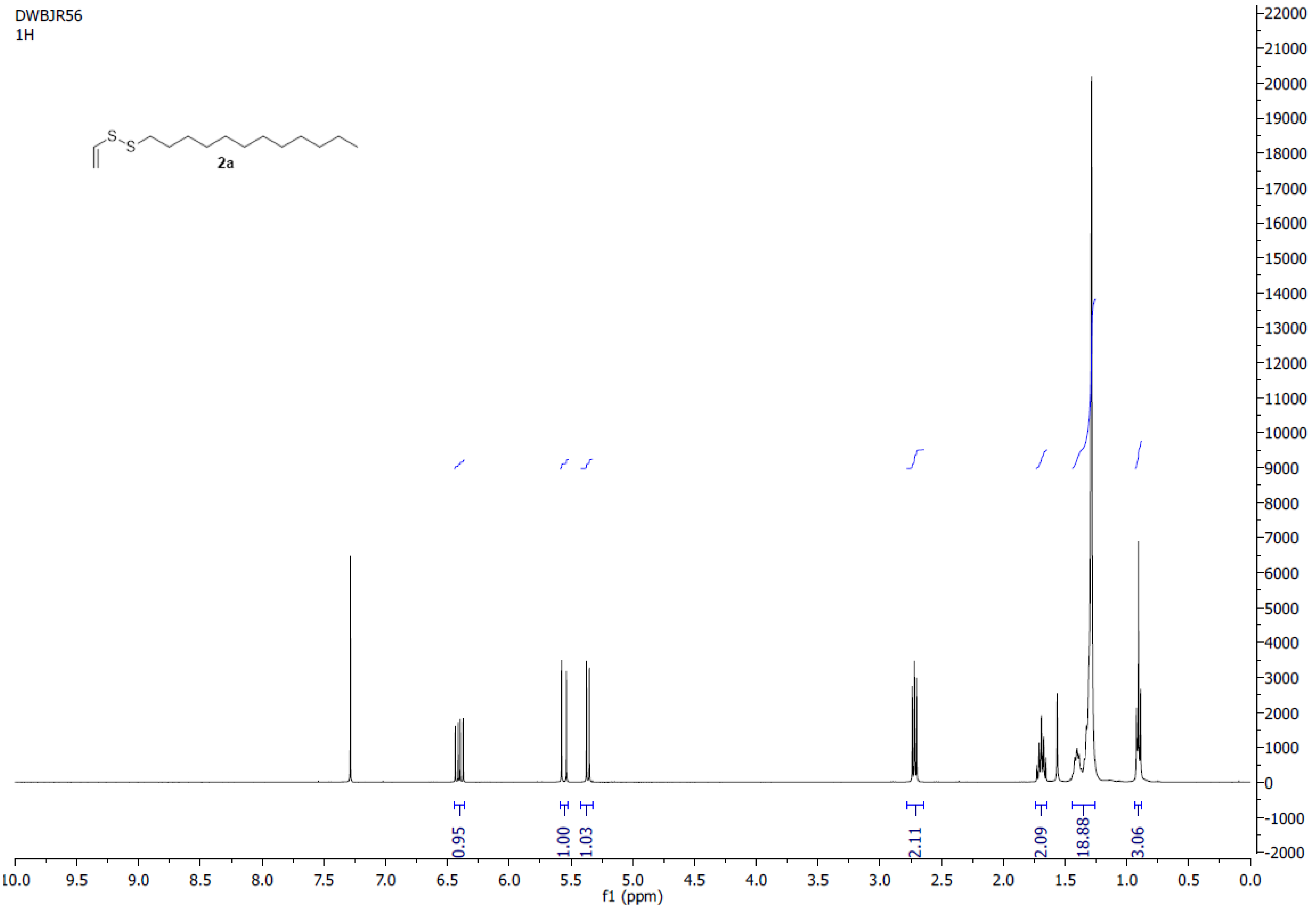

DWBJR56
1H
S
S
2a
0.95
1.00
1.03
2.11
2.09
18.88
3.06
10.0
9.5
9.0
8.5
8.0
7.5
7.0
6.5
6.0
5.5
5.0
4.5
4.0
3.5
3.0
2.5
2.0
1.5
1.0
0.5
0.0
f1 (ppm)
22000
21000
20000
19000
18000
17000
16000
15000
14000
13000
12000
11000
10000
9000
8000
7000
6000
5000
4000
3000
2000
1000
0
-1000
-2000

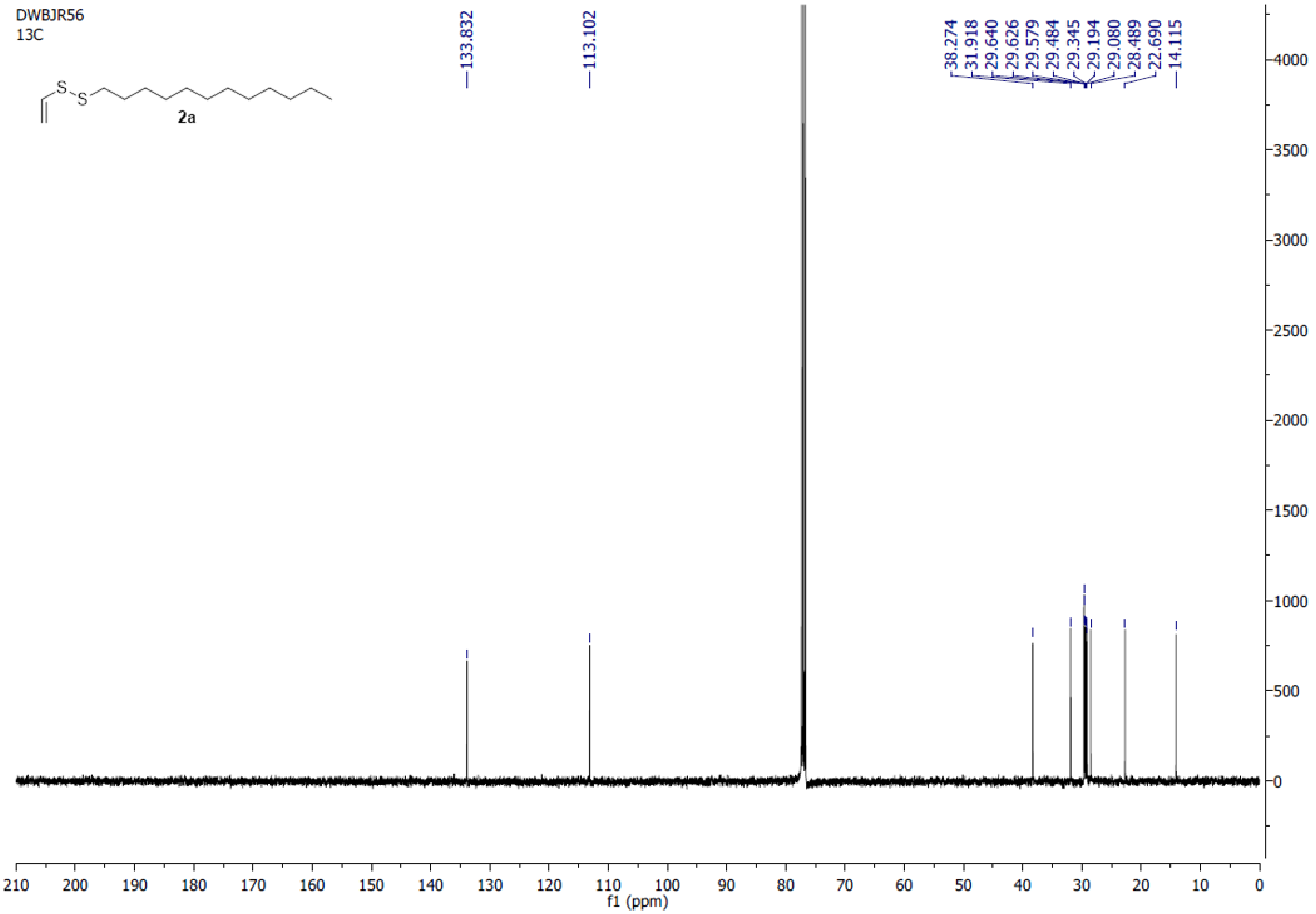
DWBJR56
13C
S
S
2a
133.832
113.102
38.274
31.918
29.640
29.626
29.579
29.484
29.345
29.194
29.080
28.489
22.690
14.115
4000
3500
3000
2500
2000
1500
1000
500
0
210 200 190 180 170 160 150 140 130 120 110 100 90 80 70 60 50 40 30 20 10 0
f1 (ppm)

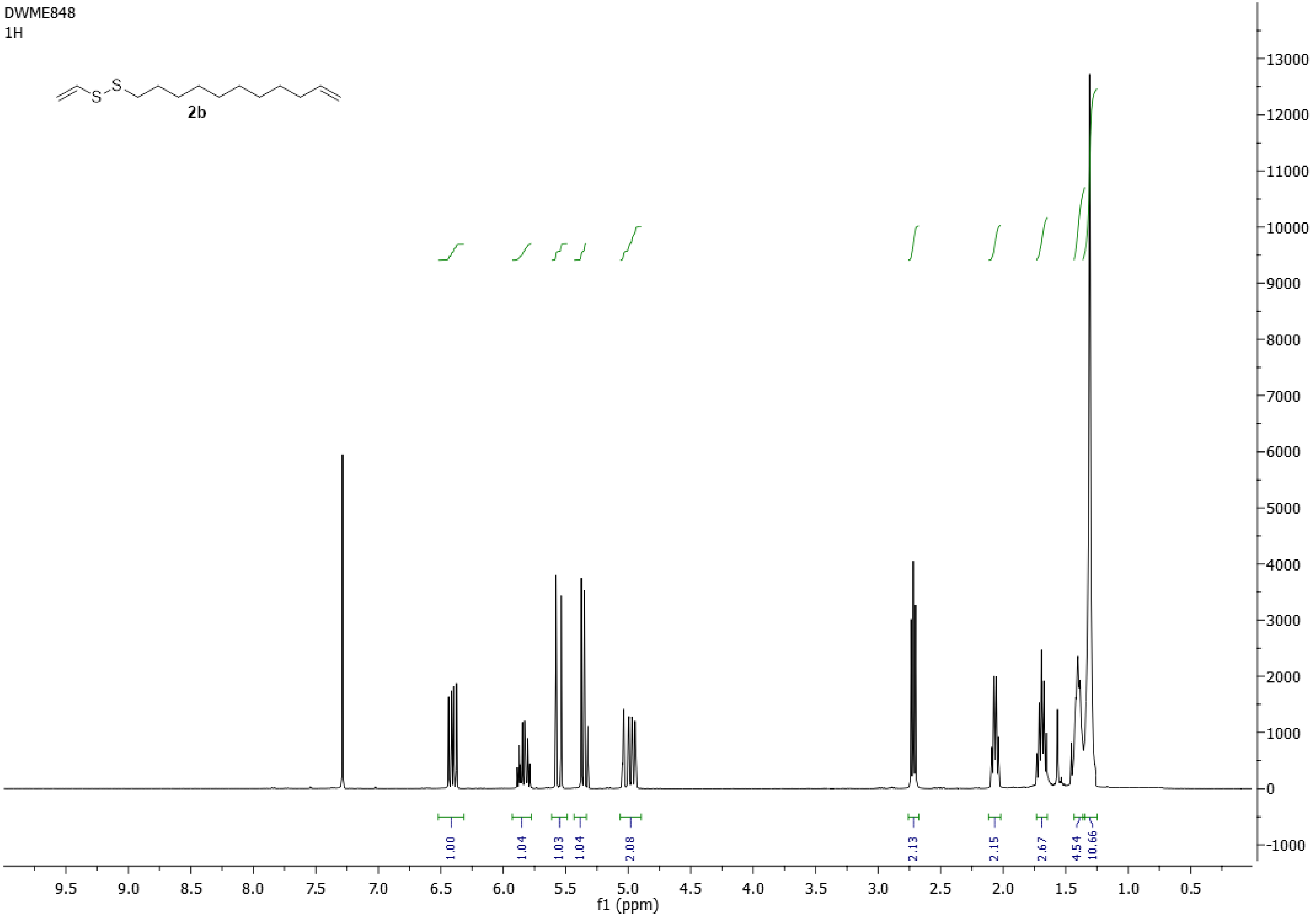

DWME848
1H
2b
1.00
1.04
1.03
1.04
2.08
2.13
2.15
2.67
4.54
10.66
f1 (ppm)

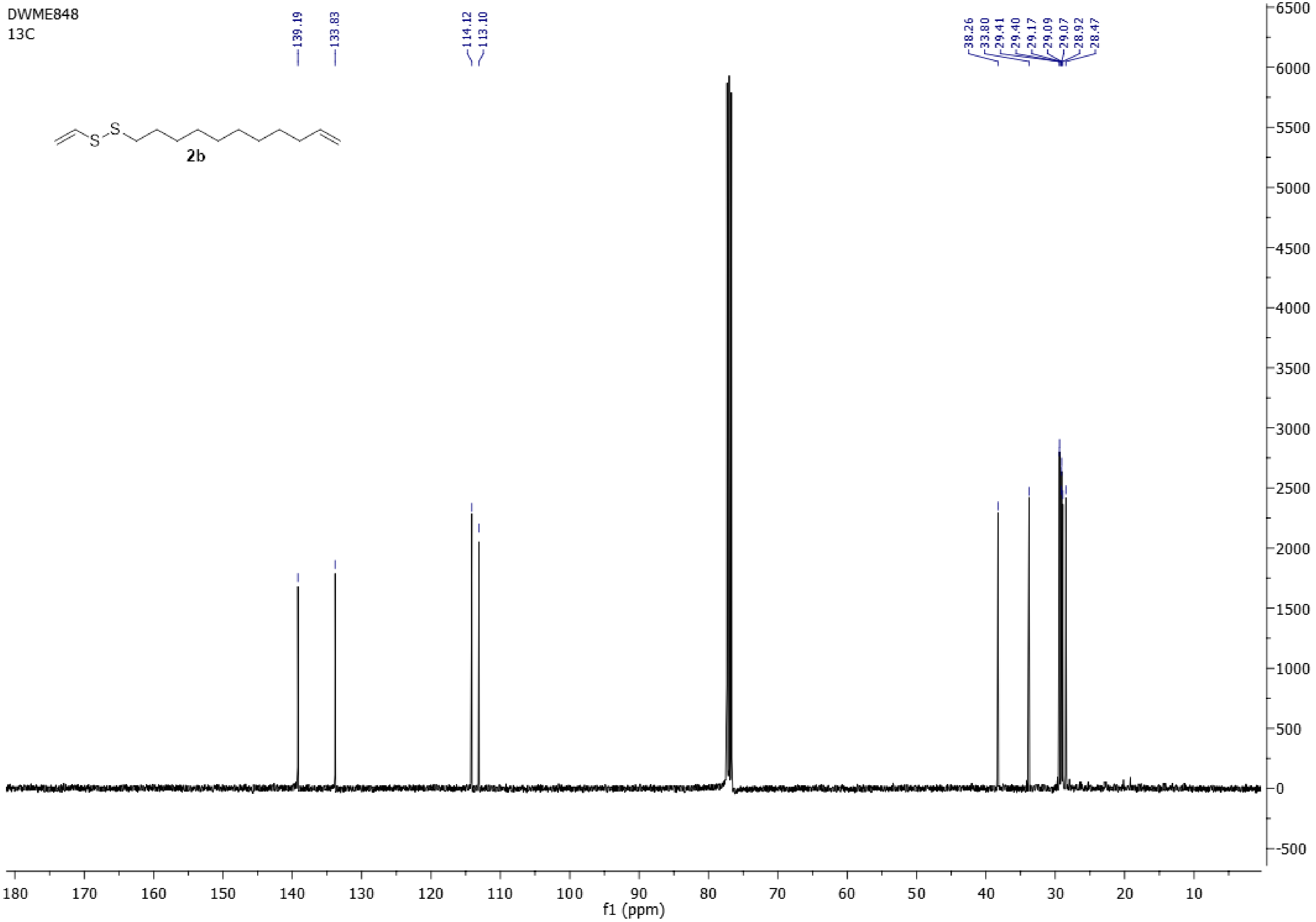
DWME848
13C
139.19
133.83
114.12
113.10
38.26
33.80
29.41
29.40
29.17
29.09
29.07
28.92
28.47
2b
6500
6000
5500
5000
4500
4000
3500
3000
2500
2000
1500
1000
500
0
-500
180 170 160 150 140 130 120 110 100 90 80 70 60 50 40 30 20 10
f1 (ppm)

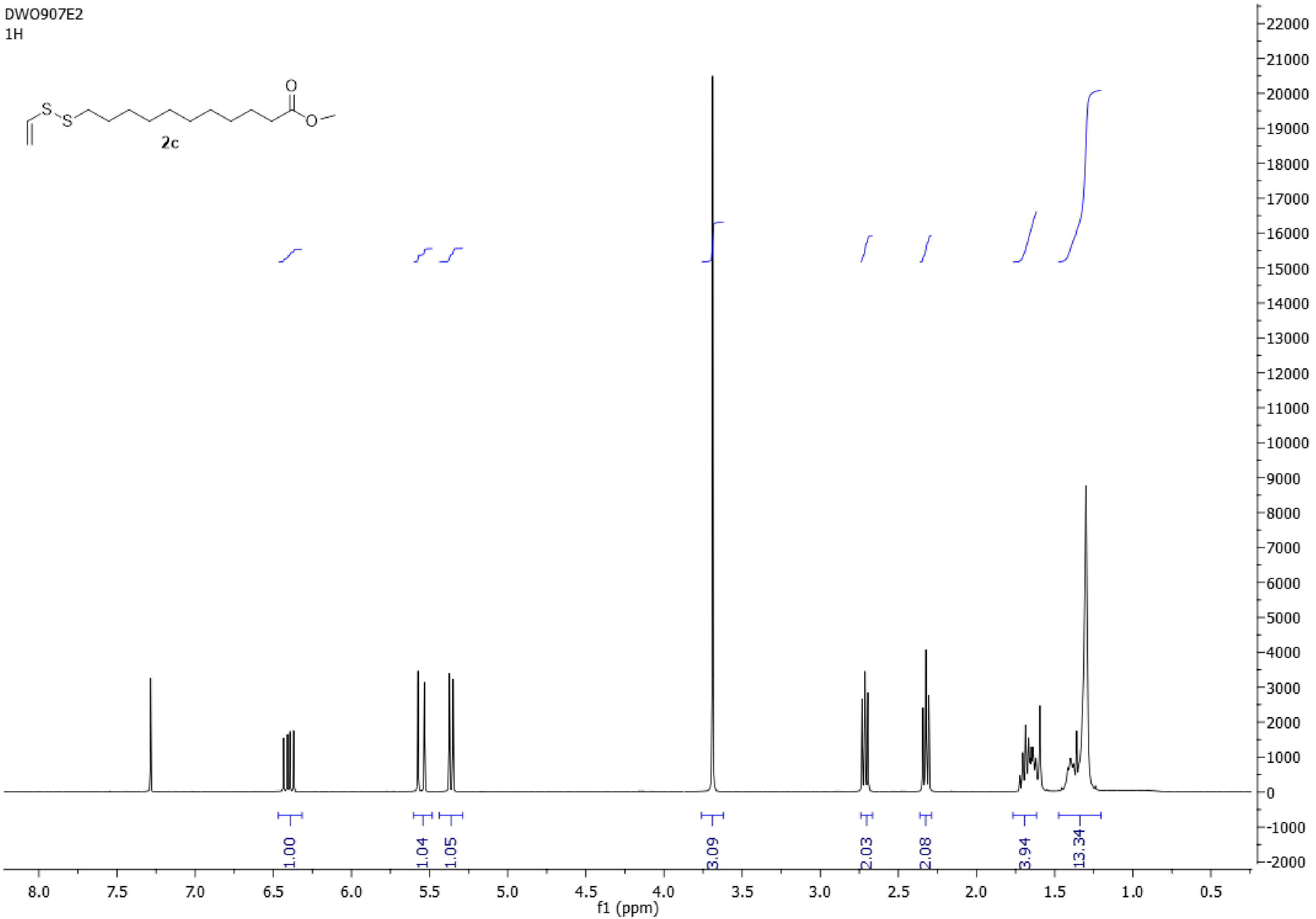
DWO907E2
1H
2c
1.00
1.04
1.05
3.09
2.03
2.08
3.94
13.34
f1 (ppm)

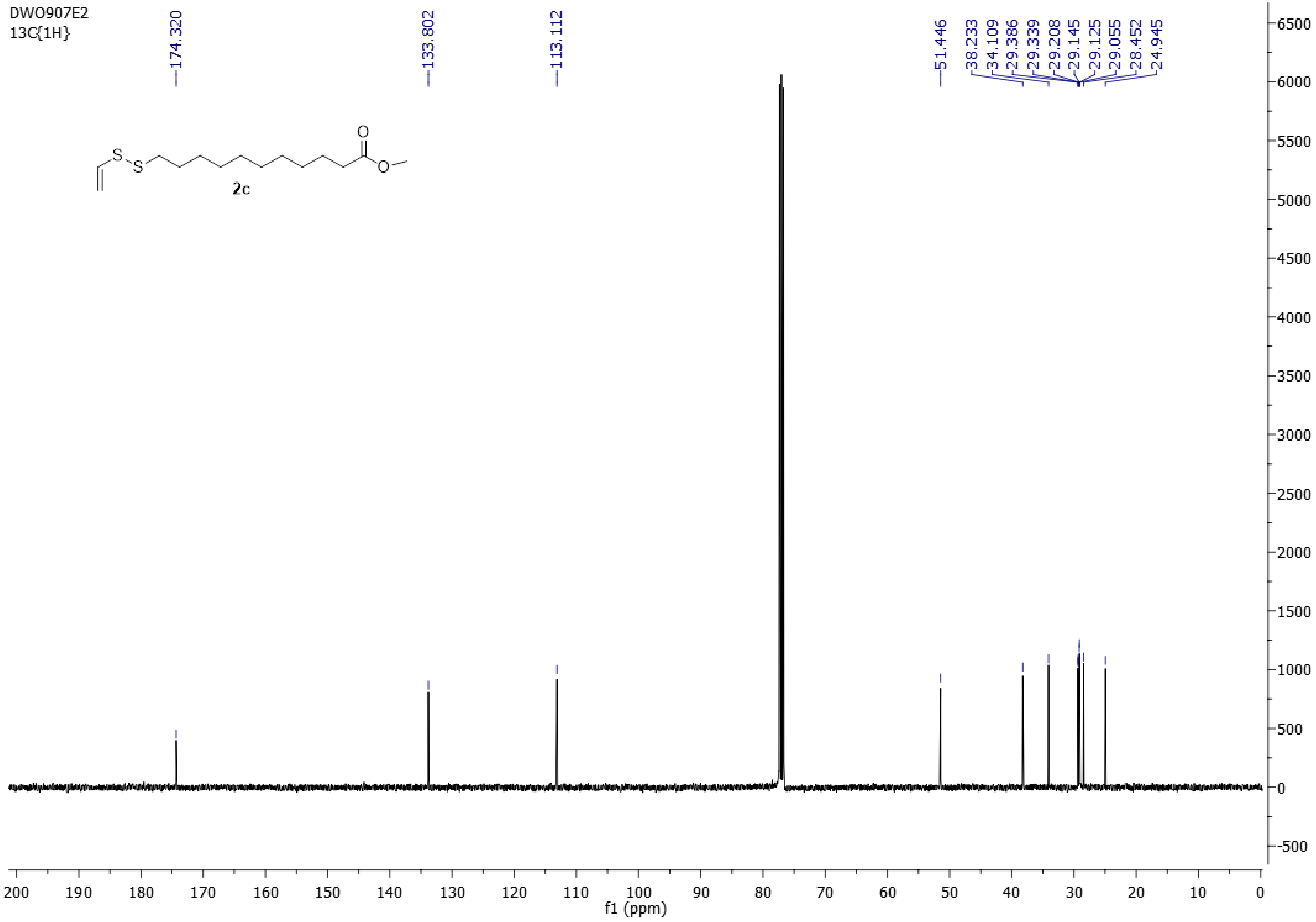
DWO907E2
13C{1H}
174.320
133.802
113.112
51.446
38.233
34.109
29.386
29.339
29.208
29.145
29.125
29.055
28.452
24.945
2c
f1 (ppm)

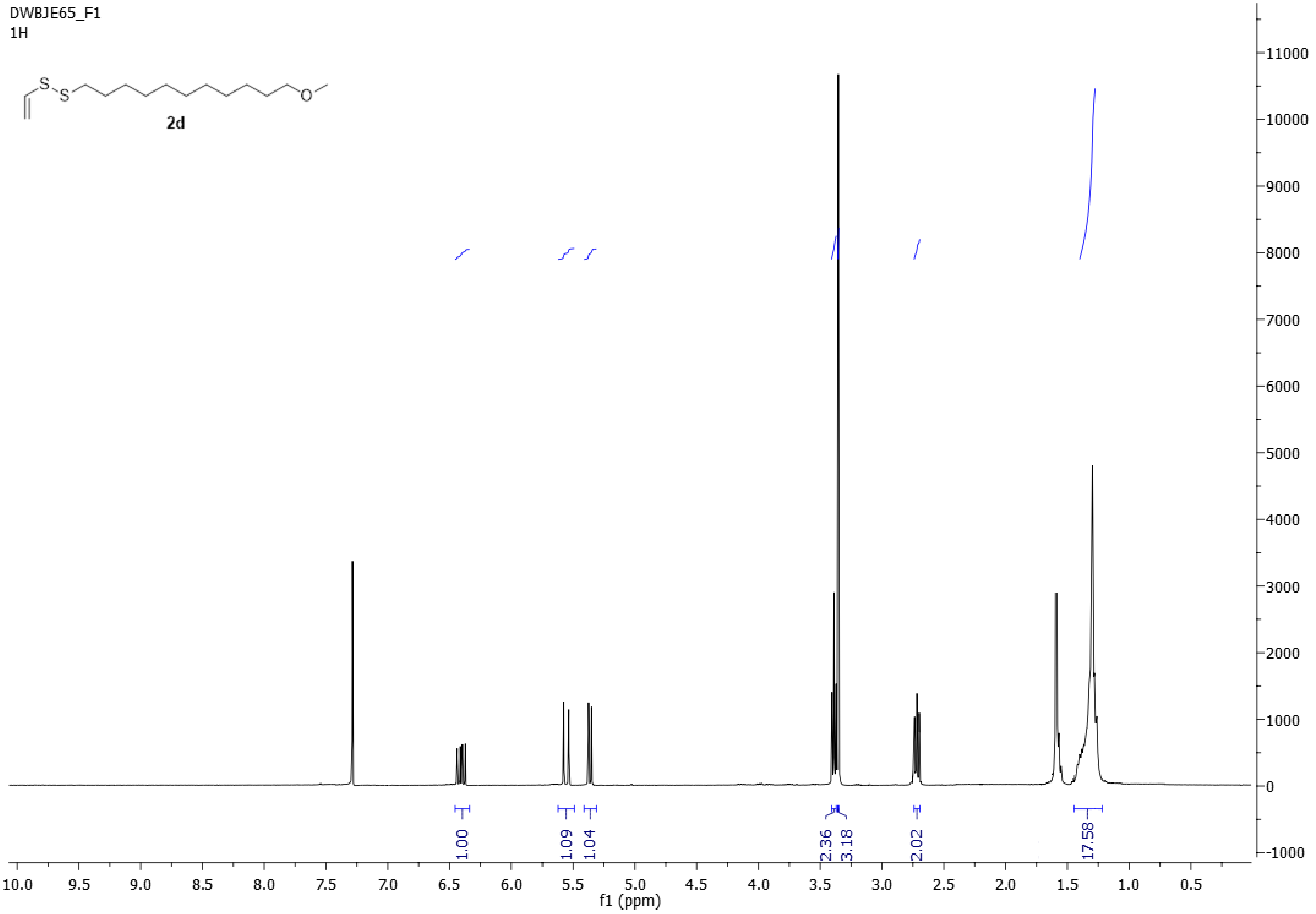
DWBJE65_F1
1H
2d
1.00
1.09
1.04
2.36
3.18
2.02
17.58
f1 (ppm)

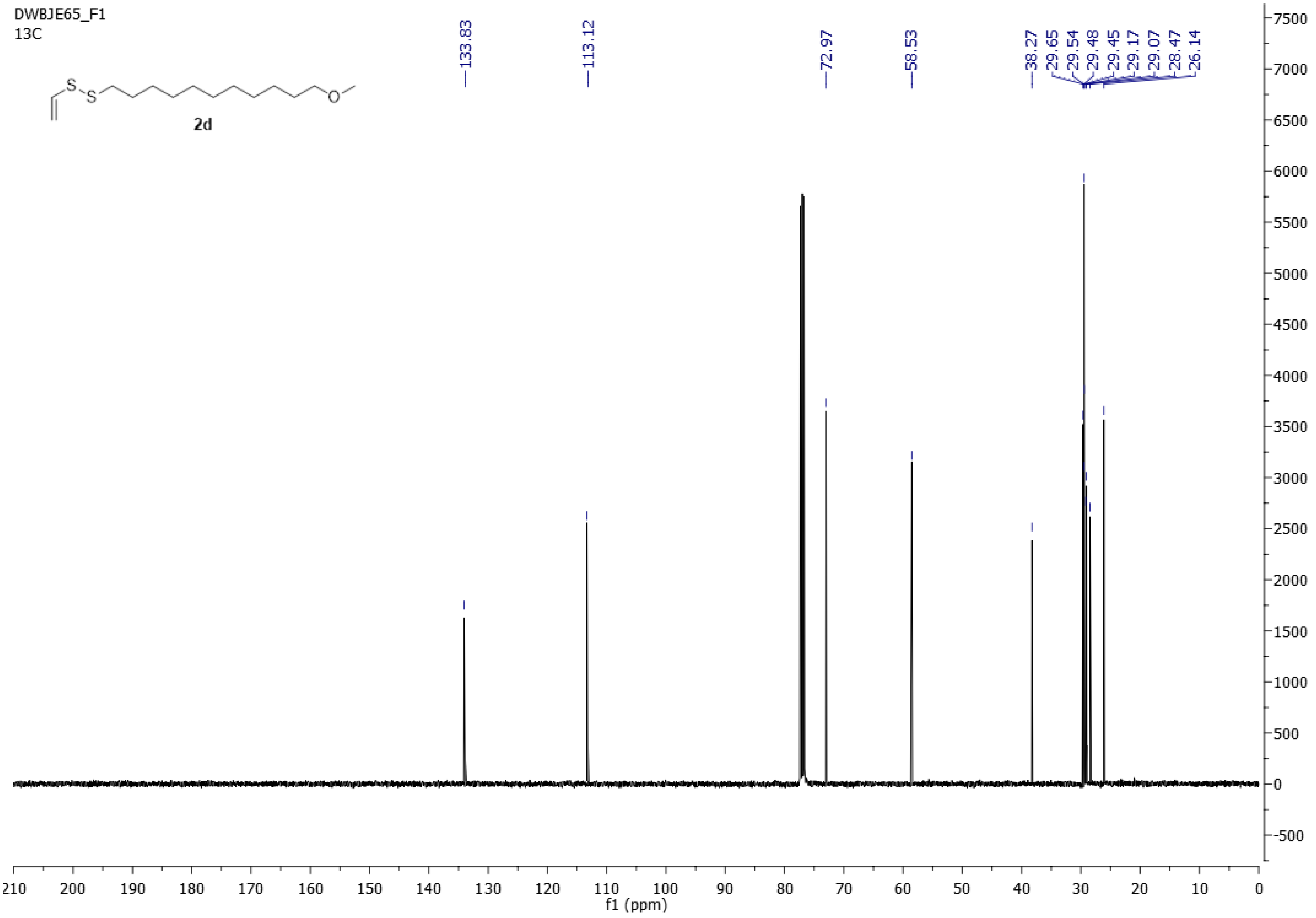
DWBJE65_F1
13C
2d
133.83
113.12
72.97
58.53
38.27
29.65
29.54
29.48
29.45
29.17
29.07
28.47
26.14
f1 (ppm)

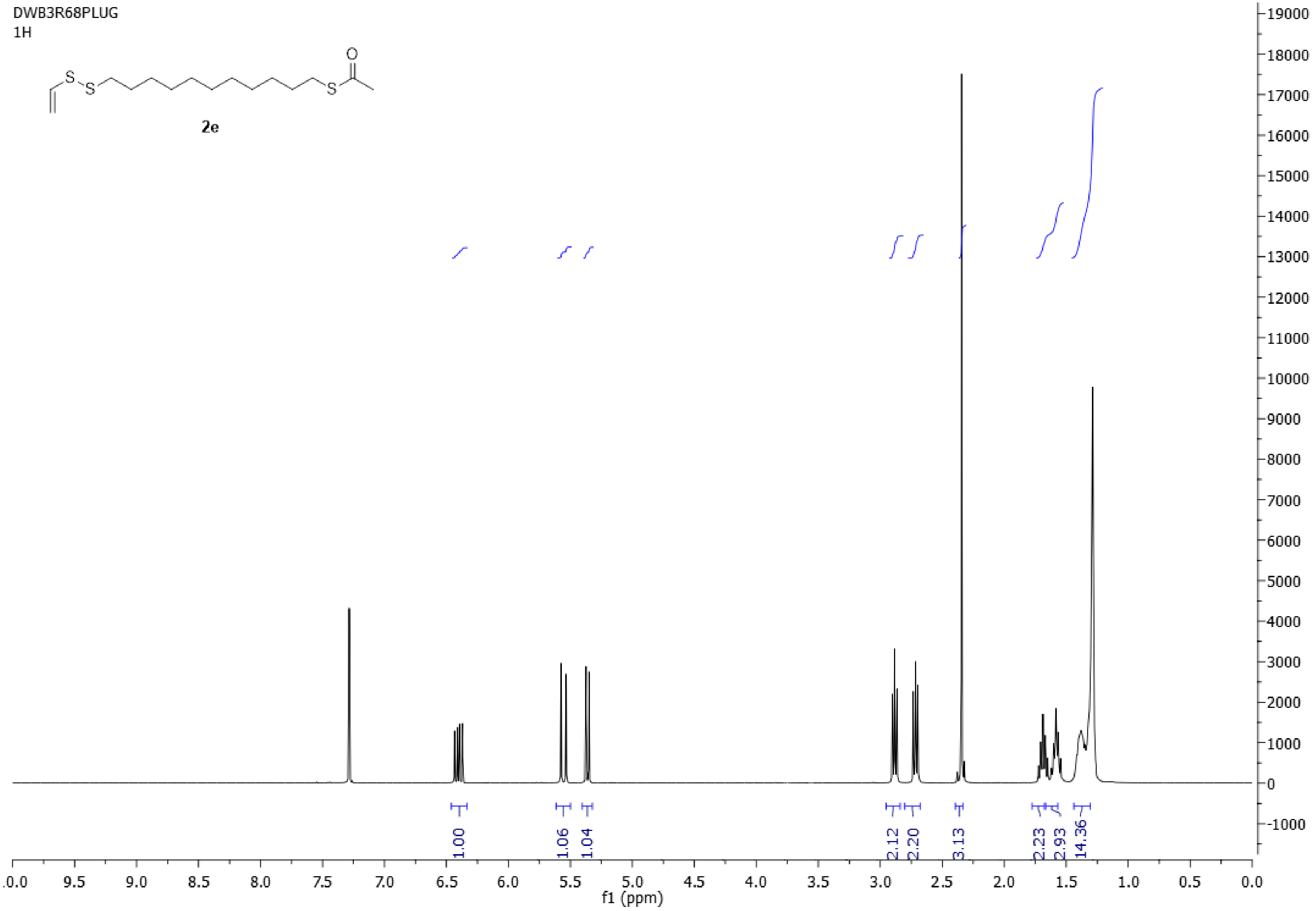
DWB3R68PLUG
1H
2e
1.00
1.06
1.04
2.12
2.20
3.13
2.23
2.93
14.36
f1 (ppm)

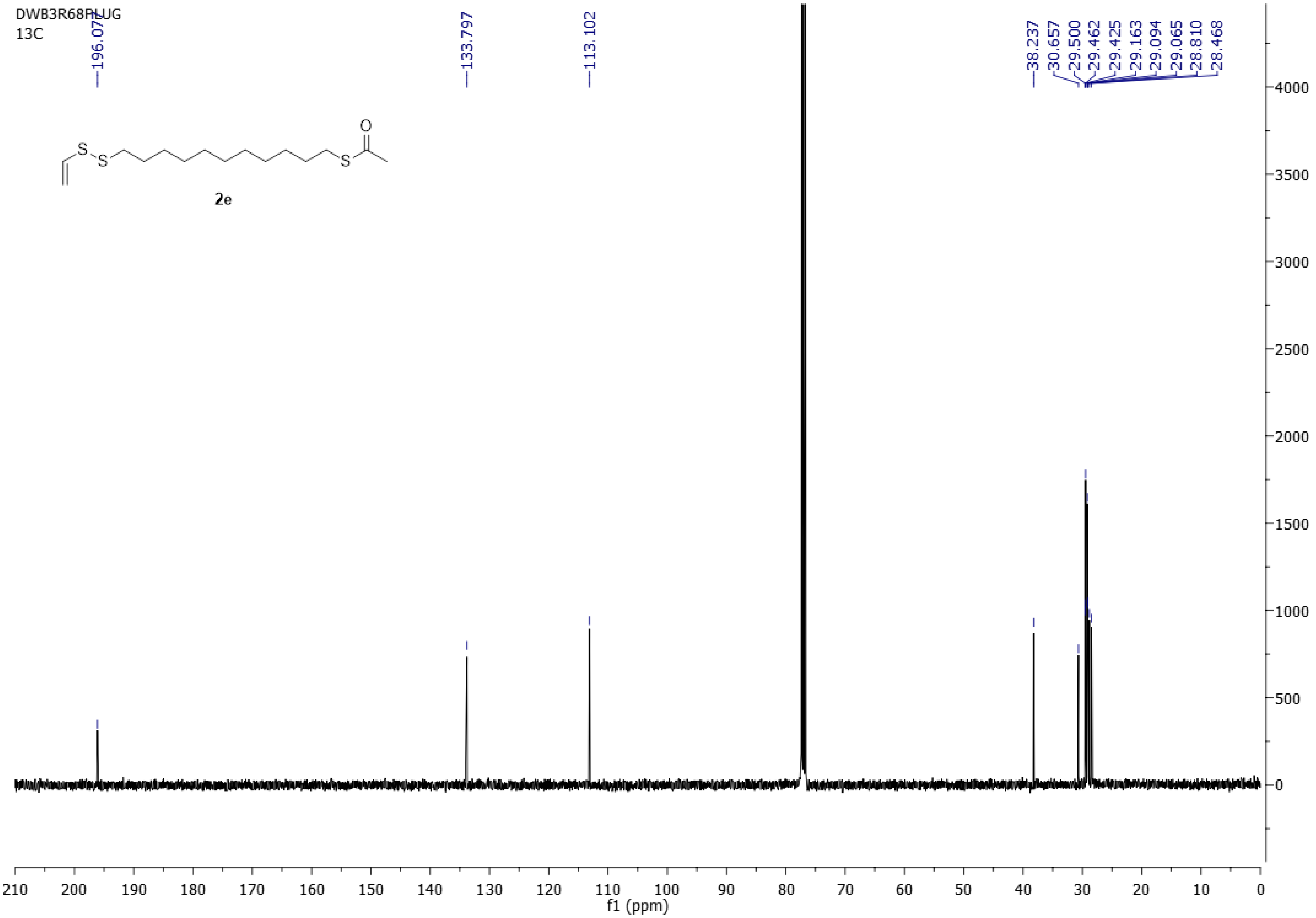
DWB3R68PLUG
13C
196.077
133.797
113.102
38.237
30.657
29.500
29.462
29.425
29.163
29.094
29.065
28.810
28.468
2e
f1 (ppm)

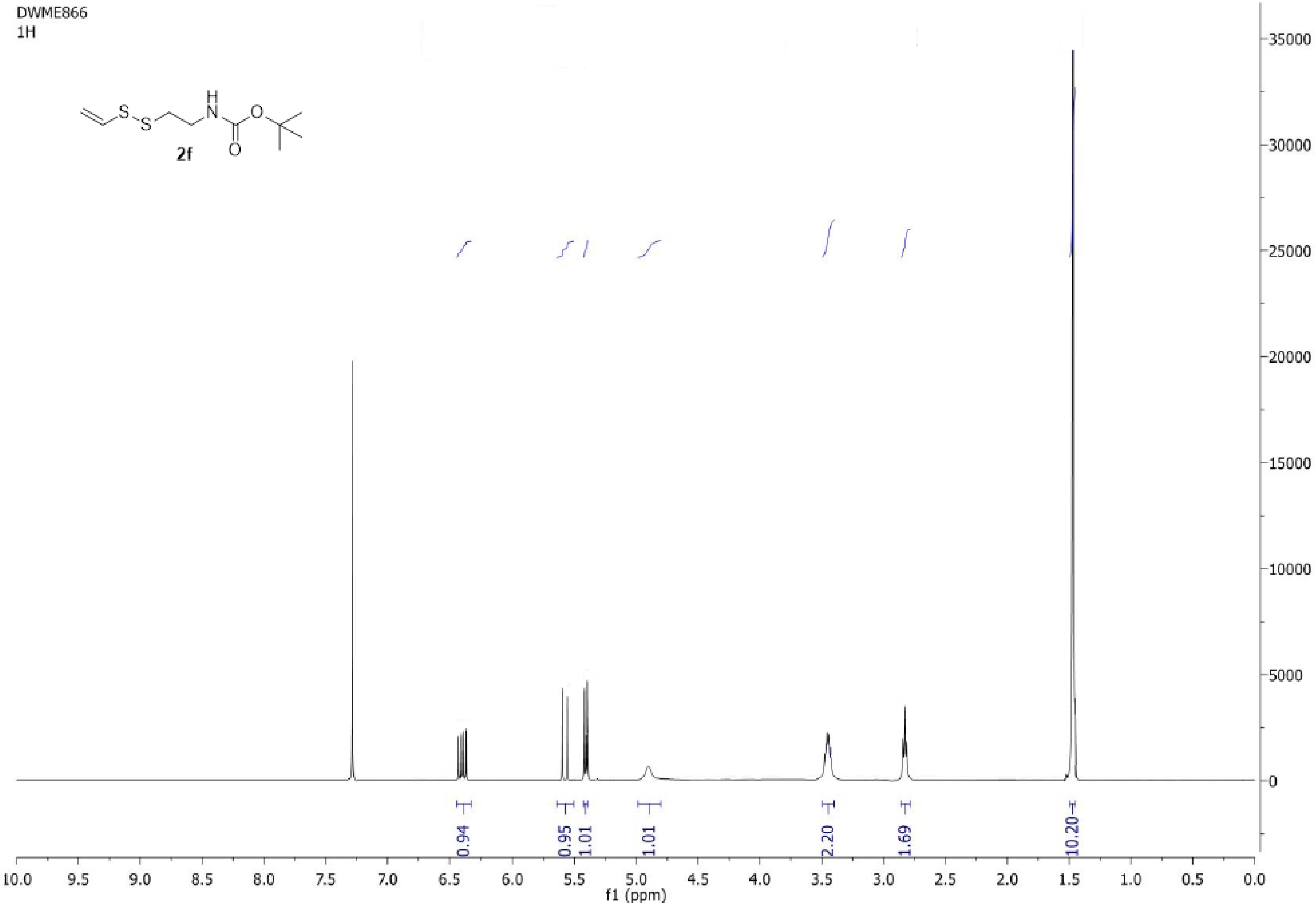
DWME866
1H
2f
0.94
0.95
1.01
1.01
2.20
1.69
10.20
f1 (ppm)

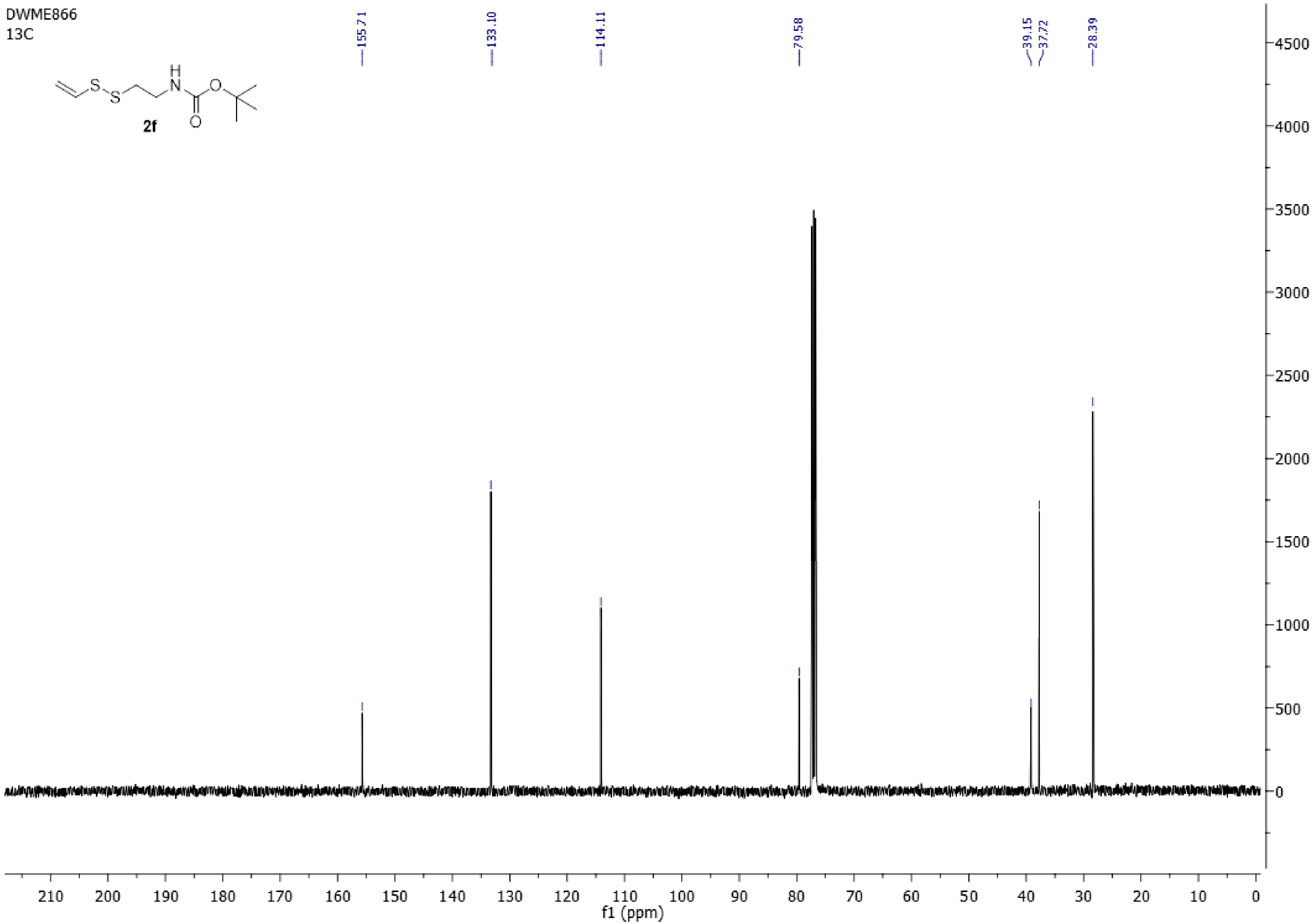

DWME866
13C
2f
155.71
133.10
114.11
79.58
39.15
37.72
28.39
f1 (ppm)

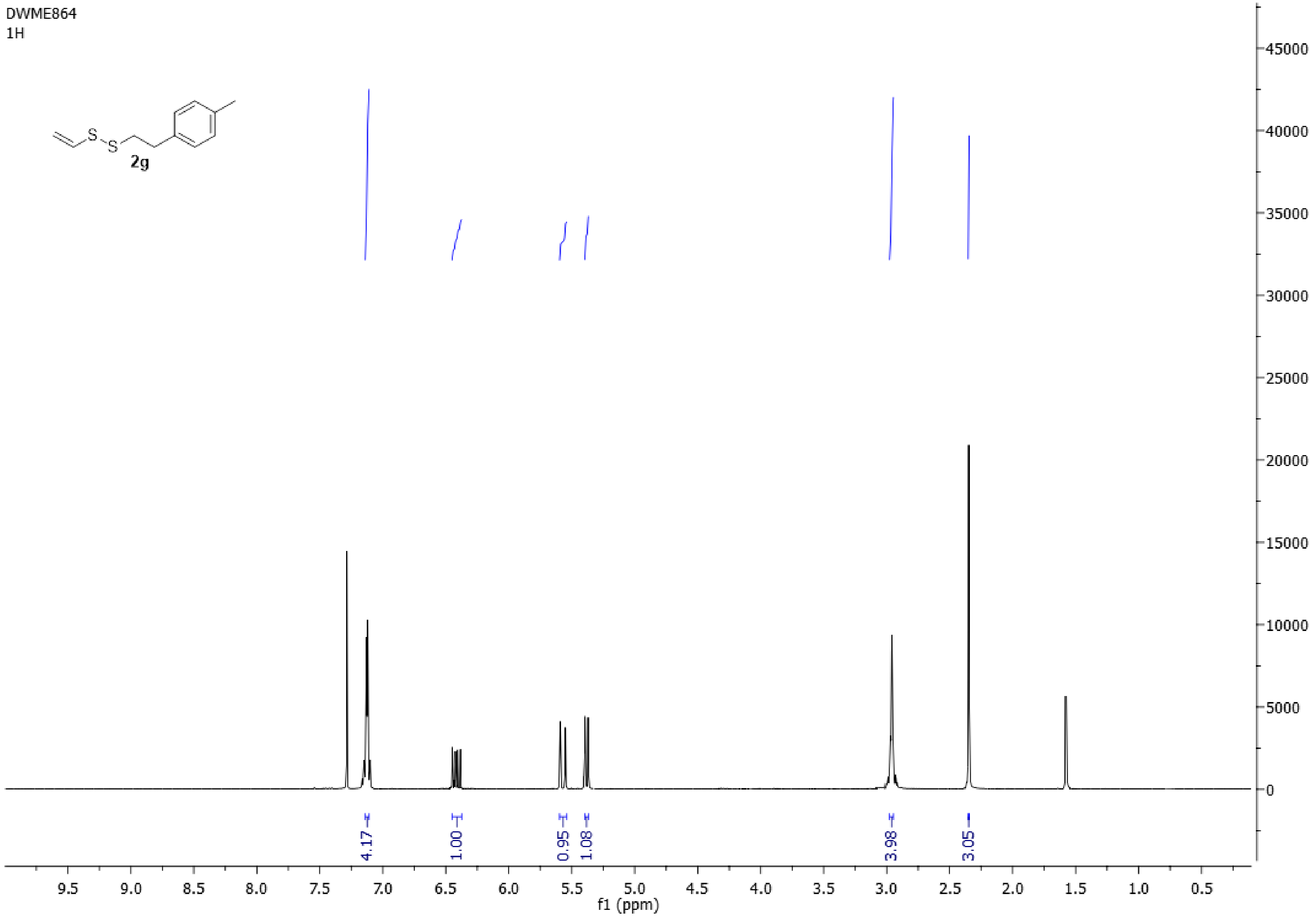

DWME864
1H
2g
4.17
1.00
0.95
1.08
3.98
3.05
45000
40000
35000
30000
25000
20000
15000
10000
5000
0
9.5
9.0
8.5
8.0
7.5
7.0
6.5
6.0
5.5
5.0
4.5
4.0
3.5
3.0
2.5
2.0
1.5
1.0
0.5
f1 (ppm)

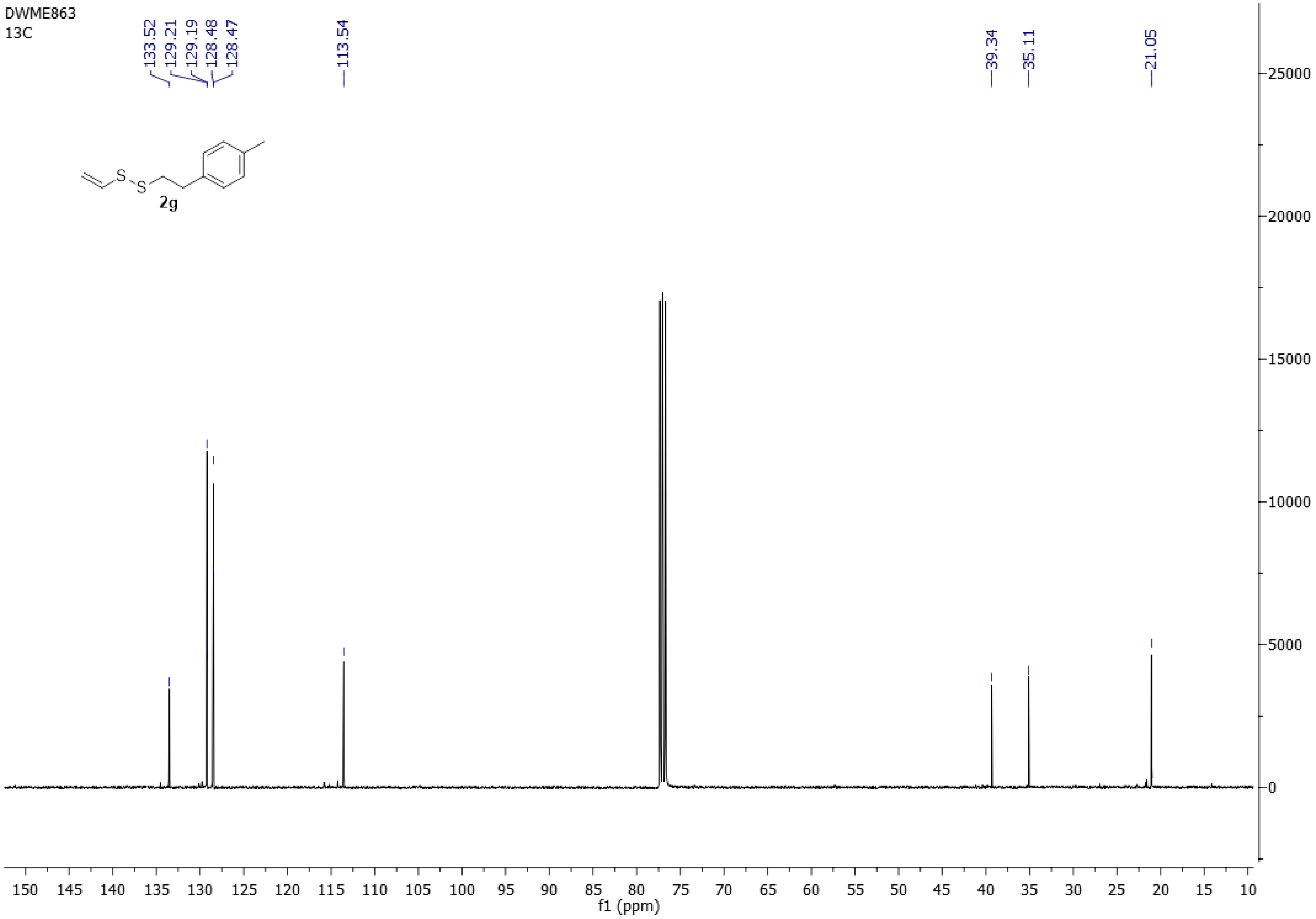
DWME863
13C
133.52
129.21
129.19
128.48
128.47
113.54
39.34
35.11
21.05
2g
25000
20000
15000
10000
5000
0
150 145 140 135 130 125 120 115 110 105 100 95 90 85 80 75 70 65 60 55 50 45 40 35 30 25 20 15 10
f1 (ppm)

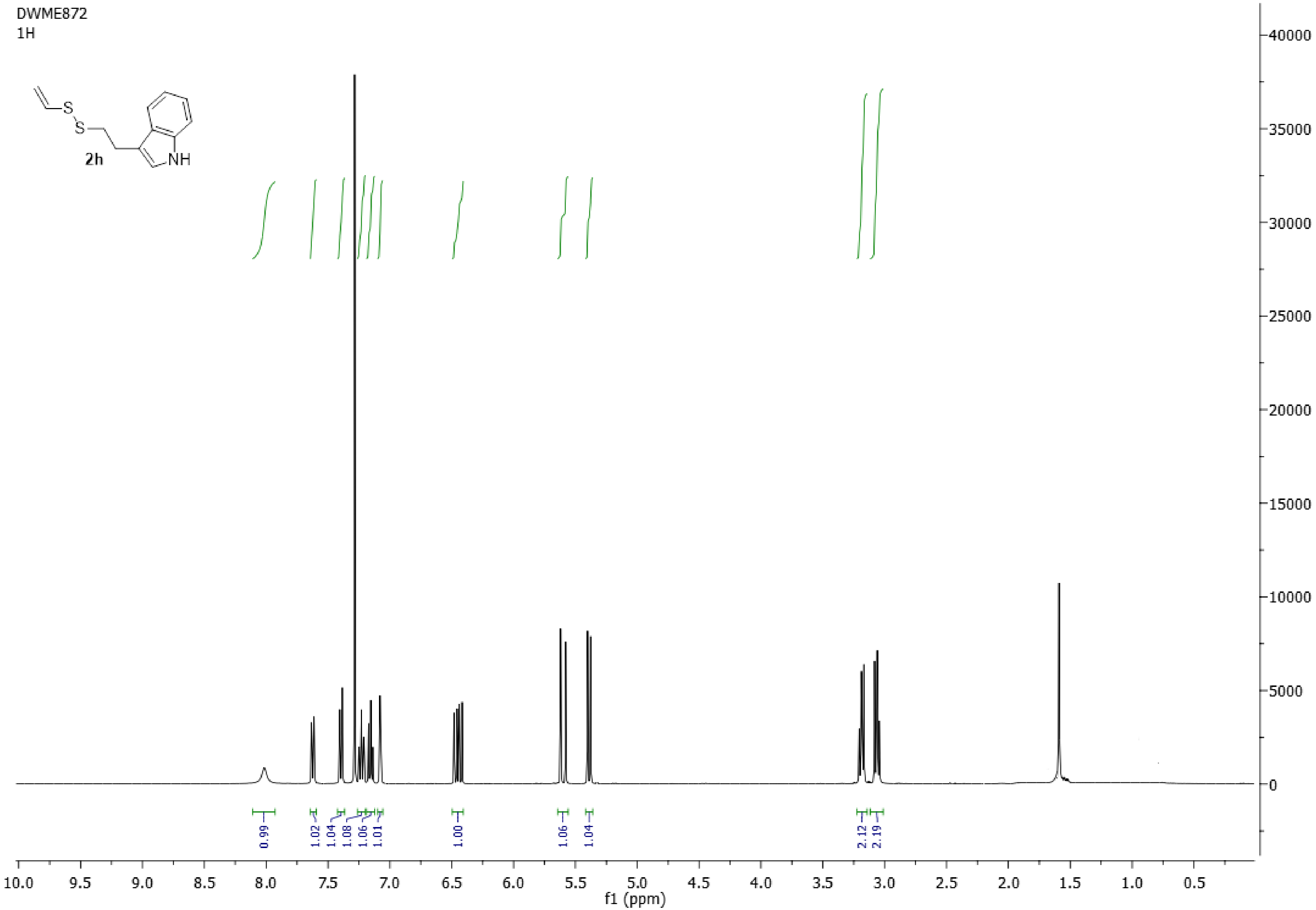
DWME872
1H
2h
NH
S
S
0.99
1.02
1.04
1.08
1.06
1.01
1.00
1.06
1.04
2.12
2.19
10.0 9.5 9.0 8.5 8.0 7.5 7.0 6.5 6.0 5.5 5.0 4.5 4.0 3.5 3.0 2.5 2.0 1.5 1.0 0.5
f1 (ppm)
40000
35000
30000
25000
20000
15000
10000
5000
0

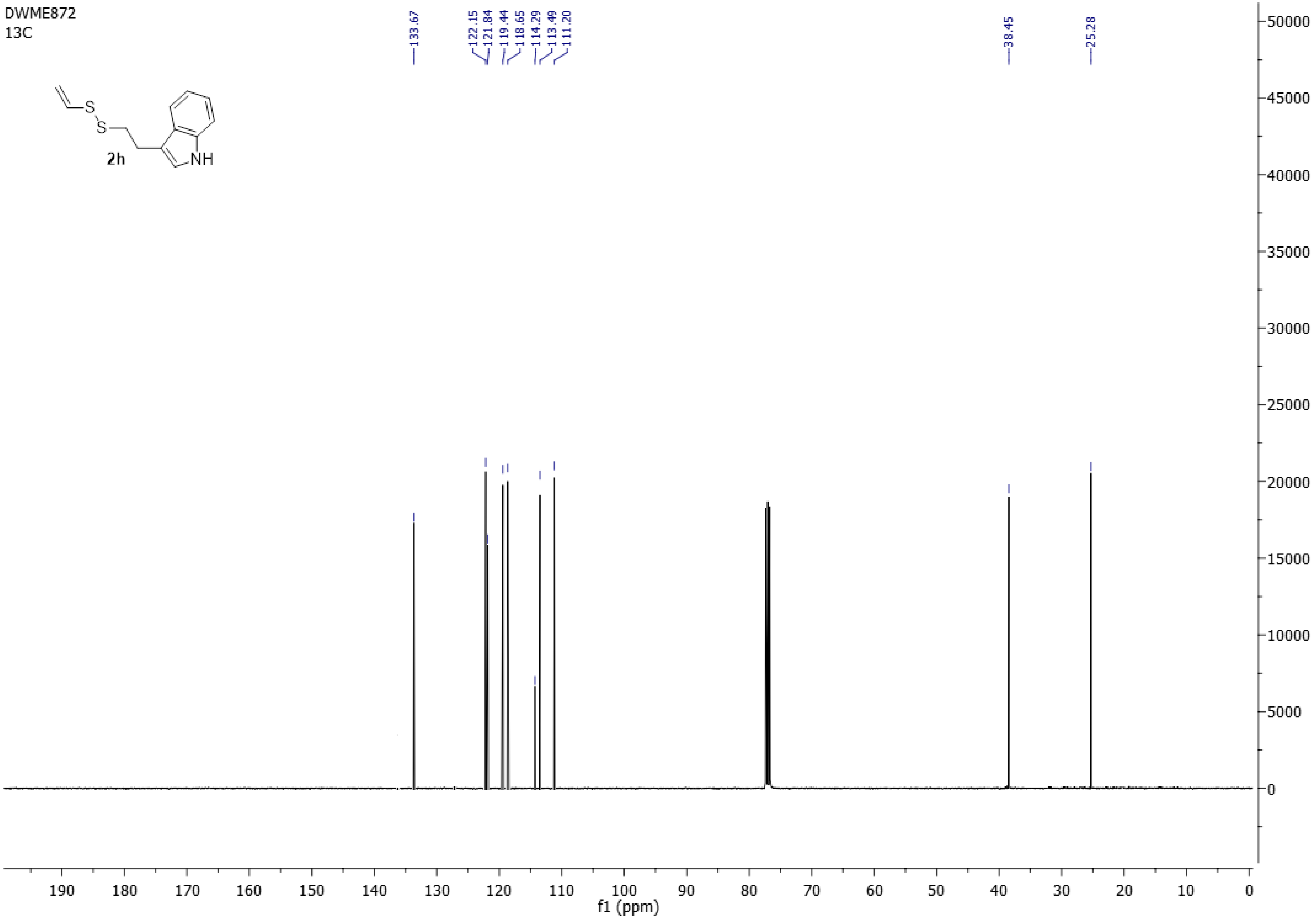
DWME872
13C
133.67
122.15
121.84
119.44
118.65
114.29
113.49
111.20
38.45
25.28
S
S
NH
2h
50000
45000
40000
35000
30000
25000
20000
15000
10000
5000
0
190 180 170 160 150 140 130 120 110 100 90 80 70 60 50 40 30 20 10 0
f1 (ppm)

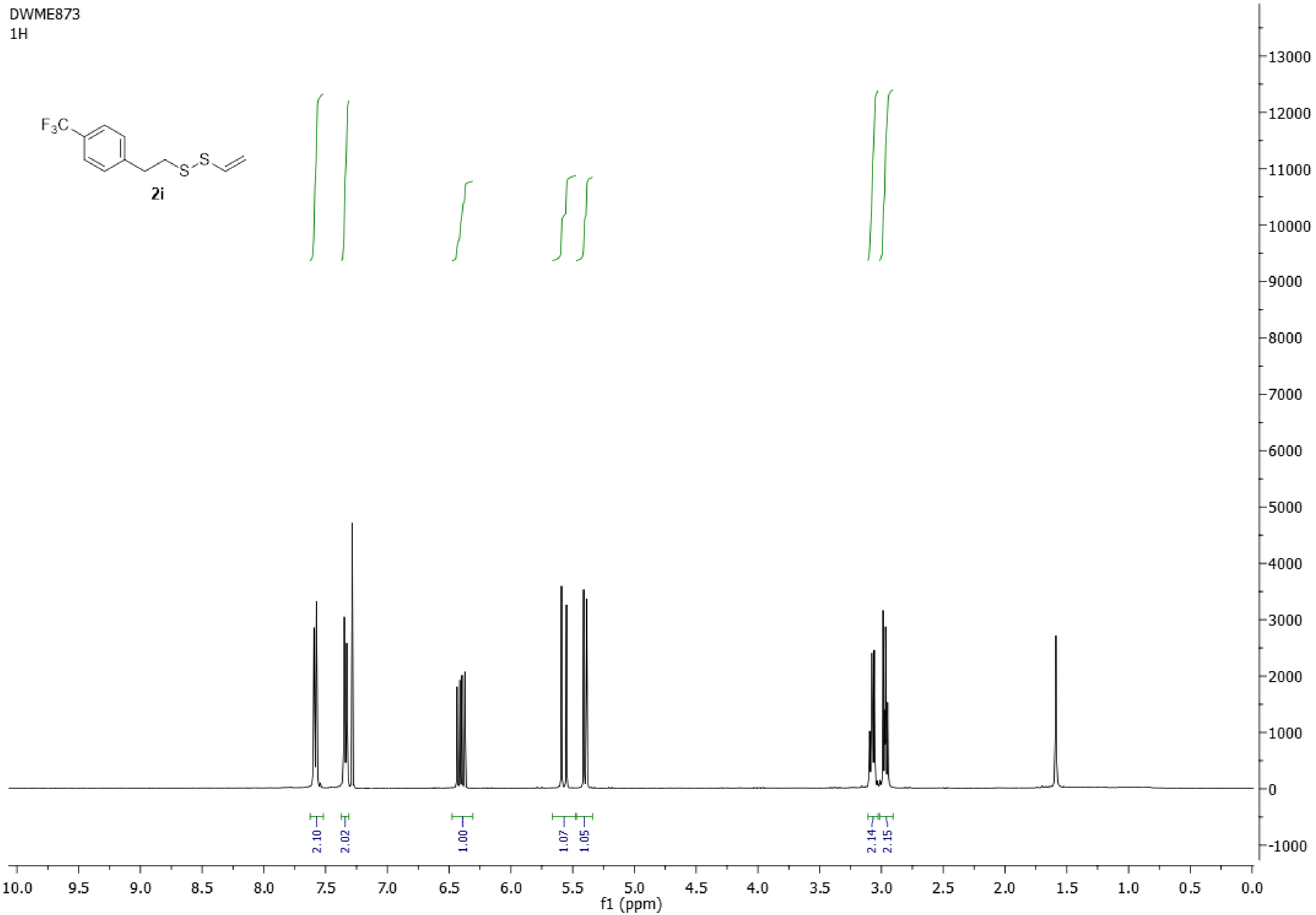

DWME873
1H
F3C
S
S
2i
13000
12000
11000
10000
9000
8000
7000
6000
5000
4000
3000
2000
1000
0
-1000
2.10
2.02
1.00
1.07
1.05
2.14
2.15
10.0
9.5
9.0
8.5
8.0
7.5
7.0
6.5
6.0
5.5
5.0
4.5
4.0
3.5
3.0
2.5
2.0
1.5
1.0
0.5
0.0
f1 (ppm)

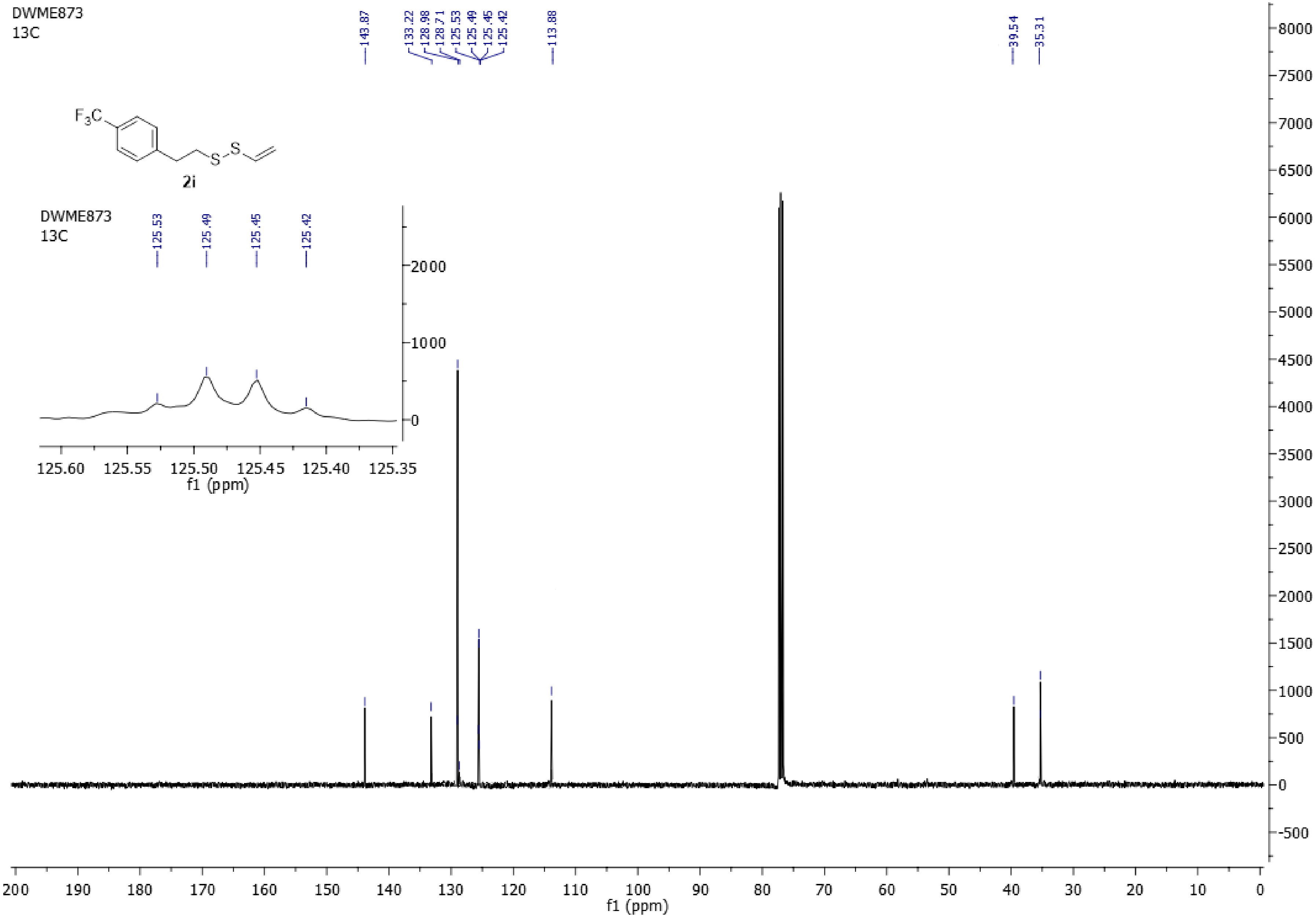
DWME873
13C
143.87
133.22
128.98
128.71
125.53
125.49
125.45
125.42
113.88
39.54
35.31
F3C
S
S
2i
DWME873
13C
125.53
125.49
125.45
125.42
2000
1000
0
125.60
125.55
125.50
125.45
125.40
125.35
f1 (ppm)
8000
7500
7000
6500
6000
5500
5000
4500
4000
3500
3000
2500
2000
1500
1000
500
0
-500
200
190
180
170
160
150
140
130
120
110
100
90
80
70
60
50
40
30
20
10
0
f1 (ppm)

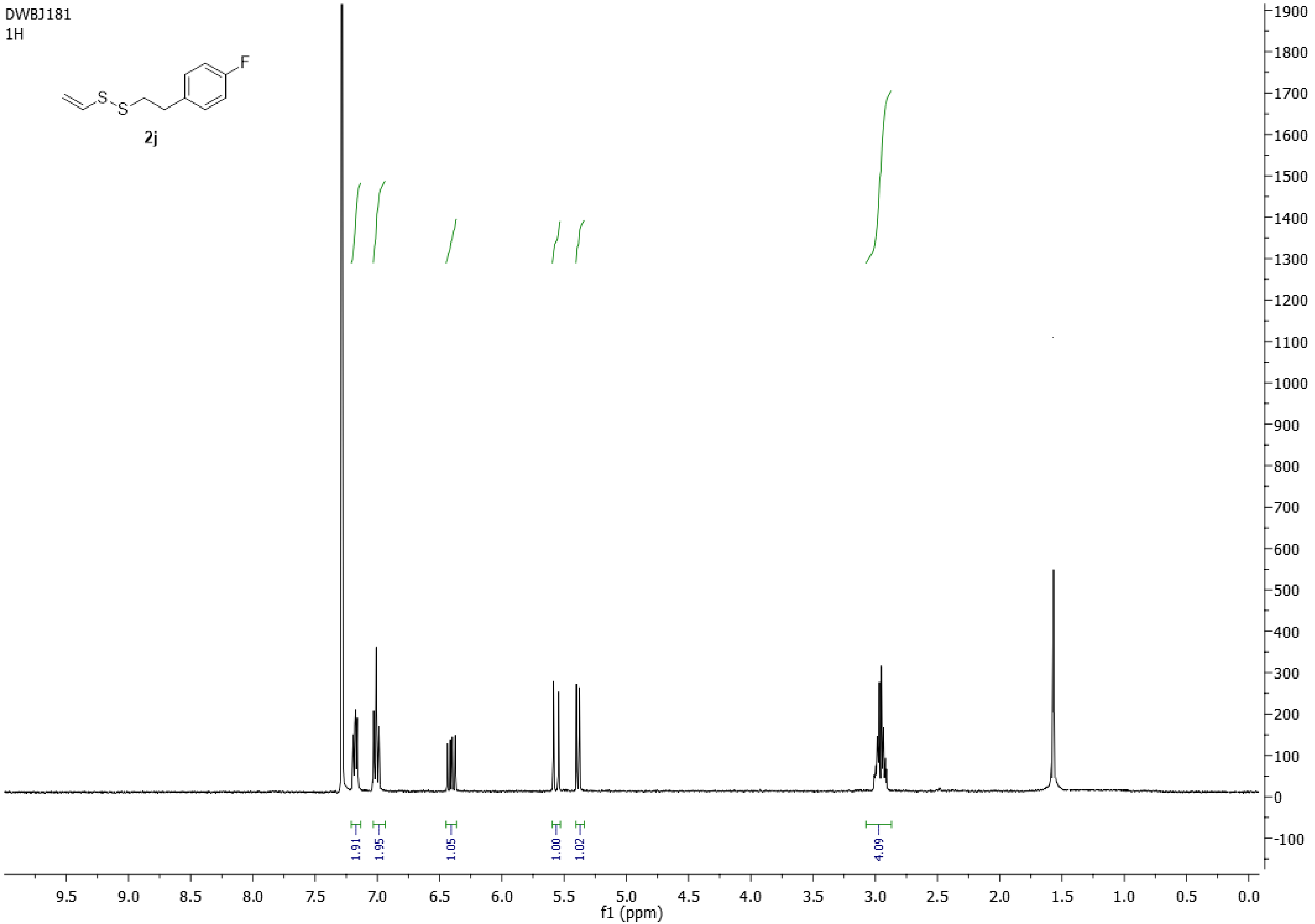
DWBJ181
1H
F
S
S
2j
1.91
1.95
1.05
1.00
1.02
4.09
f1 (ppm)

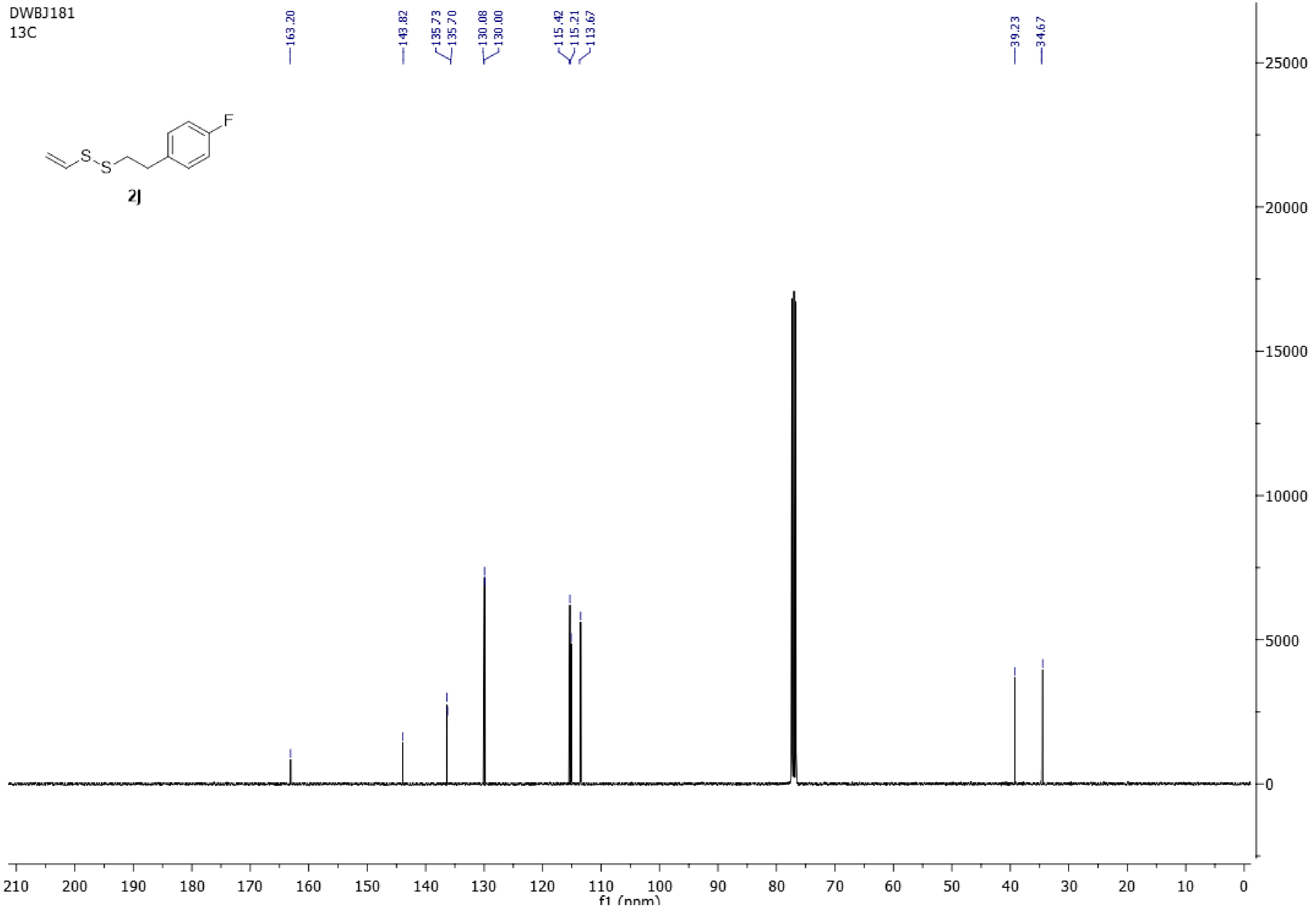
DWBJ181
13C
163.20
143.82
135.73
135.70
130.08
130.00
115.42
115.21
113.67
39.23
34.67
25000
20000
15000
10000
5000
0
210 200 190 180 170 160 150 140 130 120 110 100 90 80 70 60 50 40 30 20 10 0
f1 (ppm)
S
S
F
2j

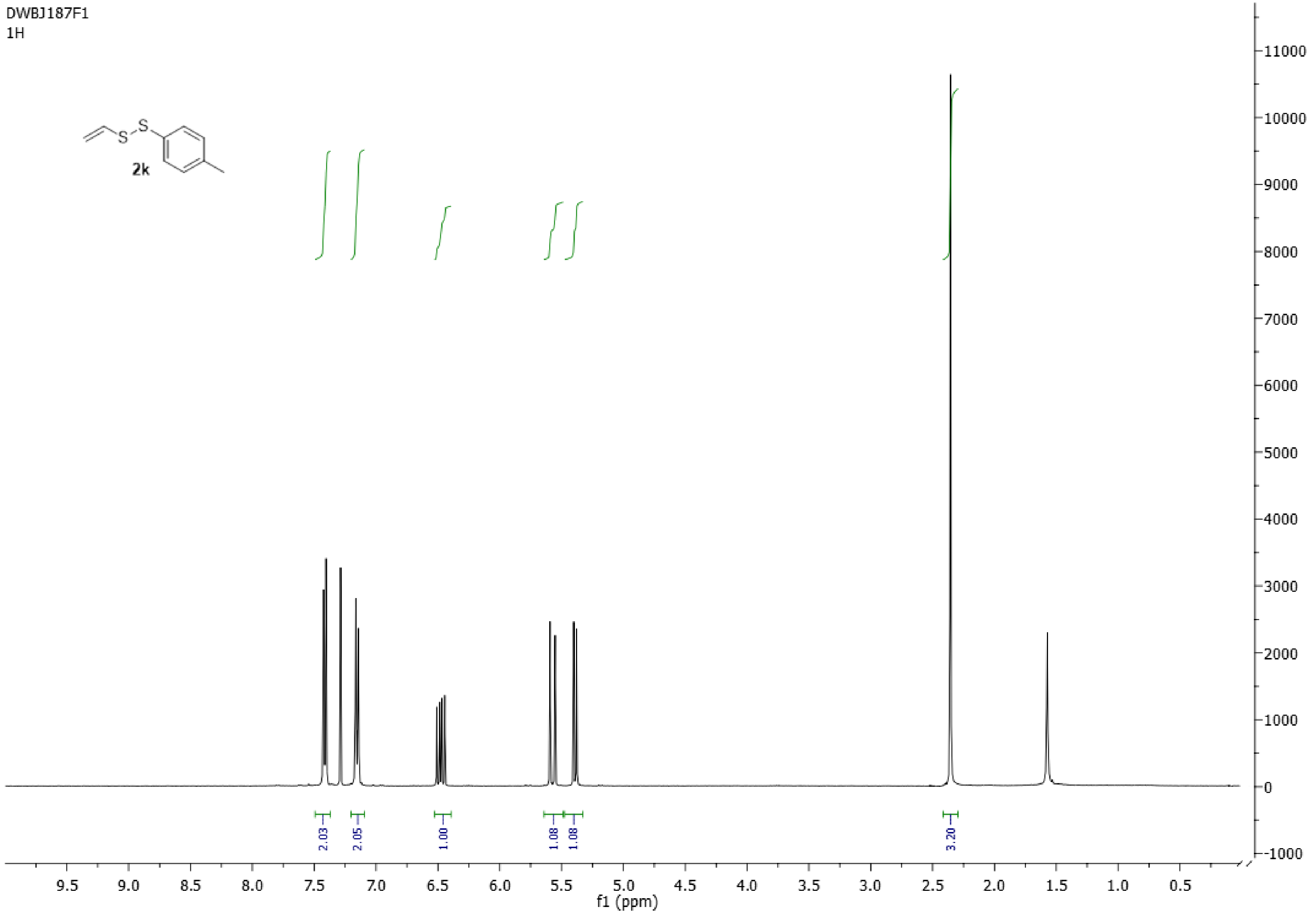
DWBJ187F1
1H
2k
2.03
2.05
1.00
1.08
1.08
3.20
f1 (ppm)

DWBJ187F1
13C{1H}

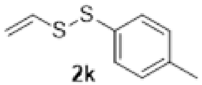
2k

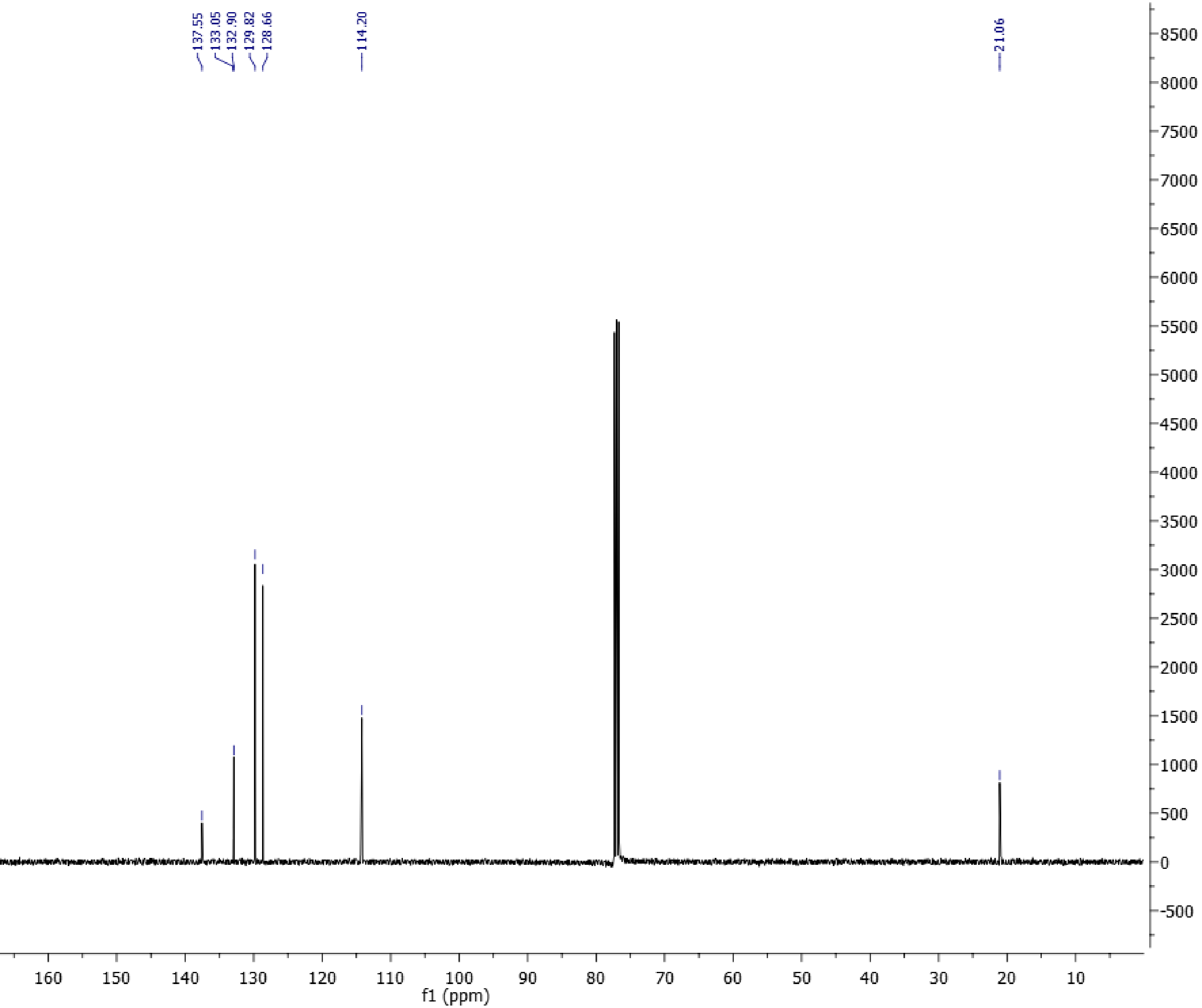

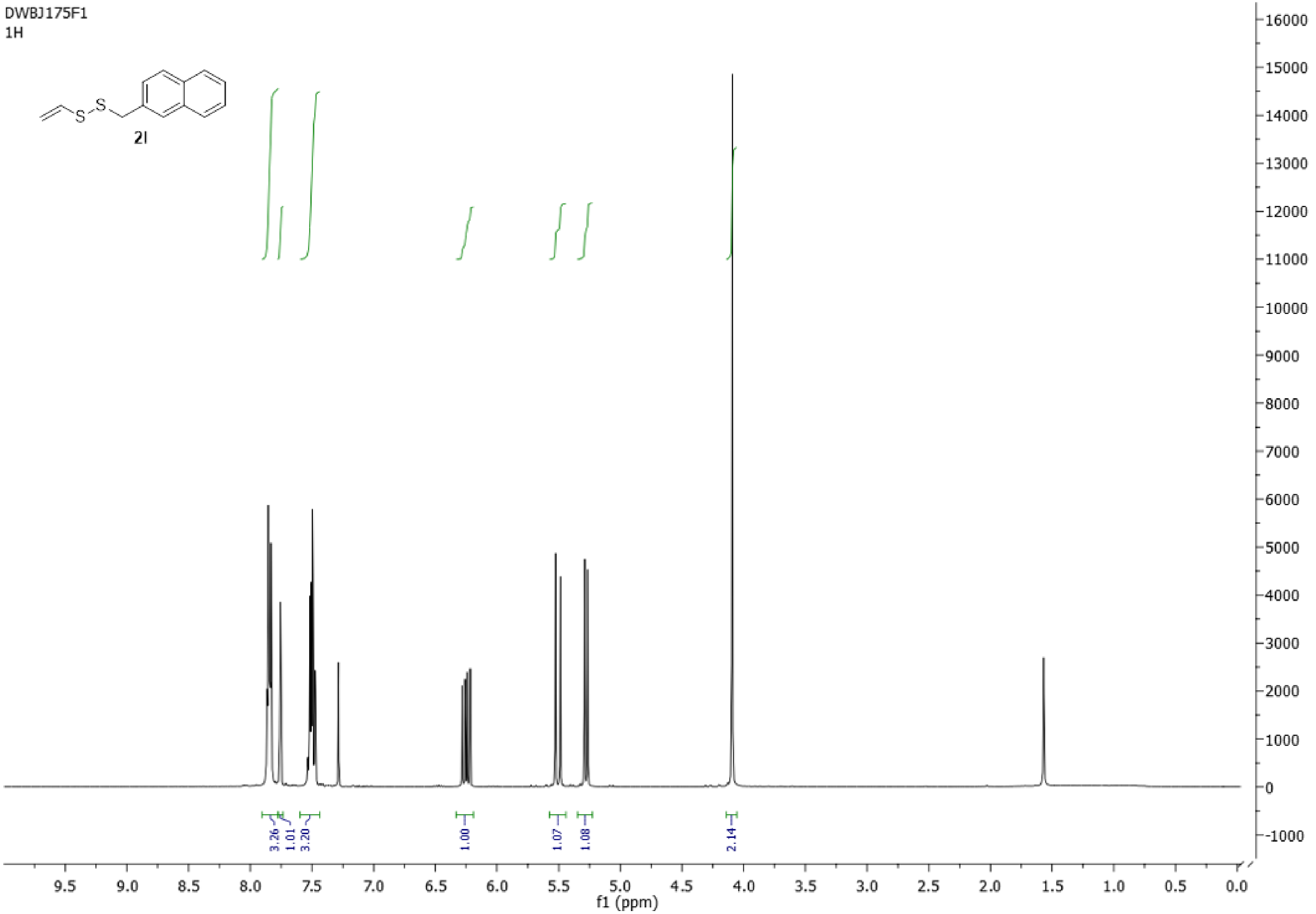
DWBJ175F1
1H
2l
3.26
1.01
3.20
1.00
1.07
1.08
2.14
f1 (ppm)

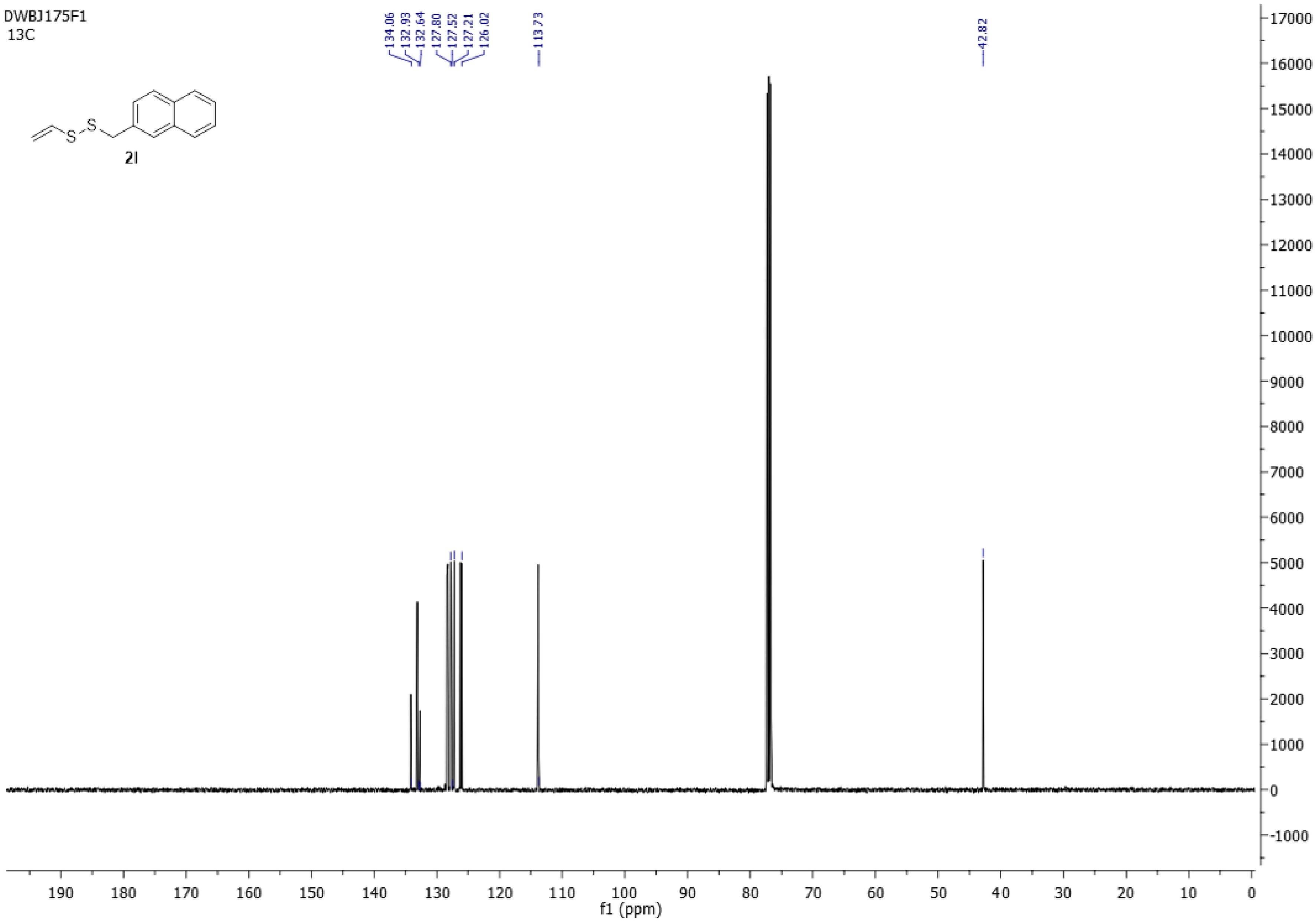
DWBJ175F1
13C
134.06
132.93
132.64
127.80
127.52
127.21
126.02
113.73
42.82
S
S
2l
f1 (ppm)

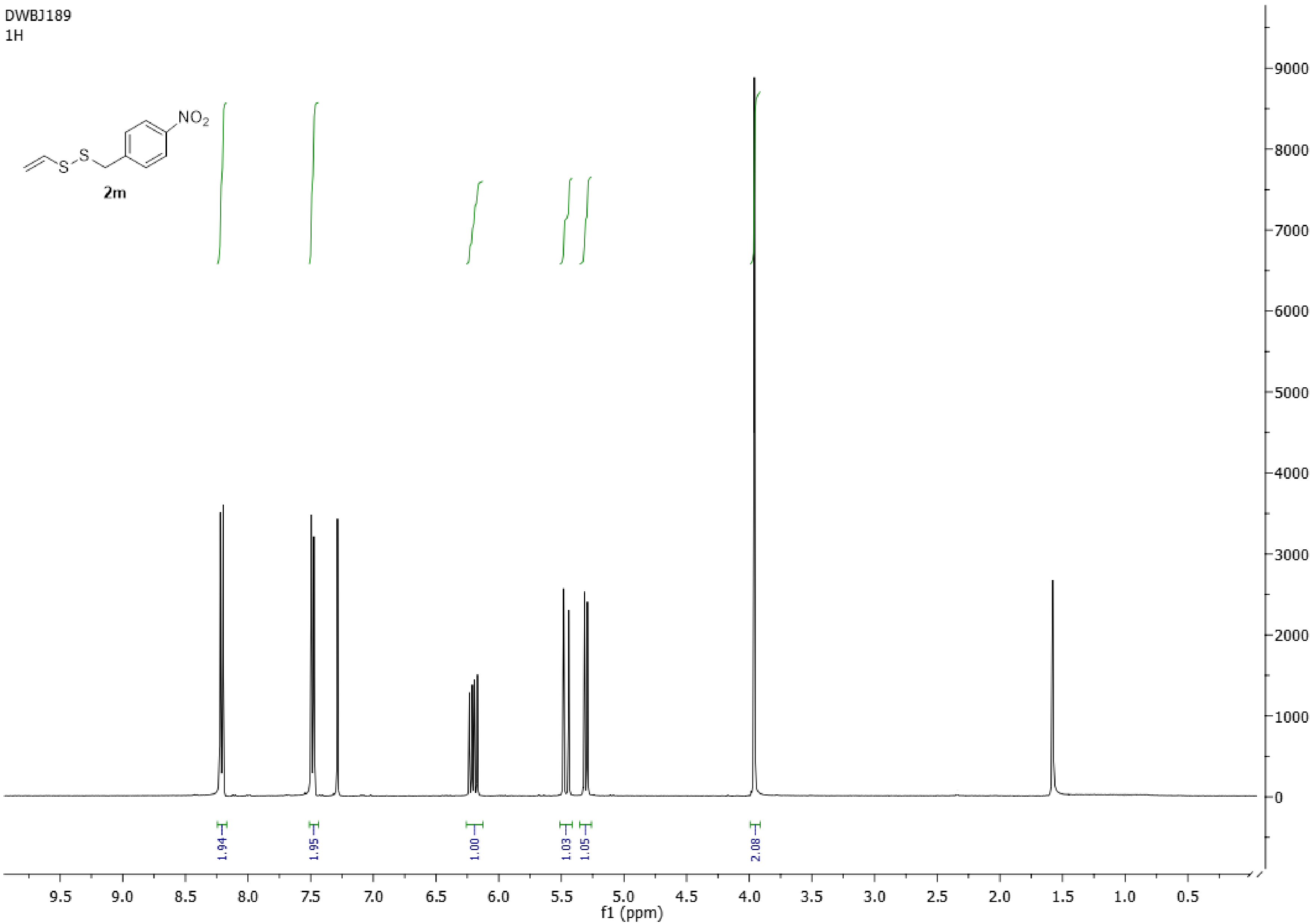
DWBJ189
1H
NO_2
S
S
2m
1.94
1.95
1.00
1.03
1.05
2.08
9000
8000
7000
6000
5000
4000
3000
2000
1000
0
9.5
9.0
8.5
8.0
7.5
7.0
6.5
6.0
5.5
5.0
4.5
4.0
3.5
3.0
2.5
2.0
1.5
1.0
0.5
f1 (ppm)

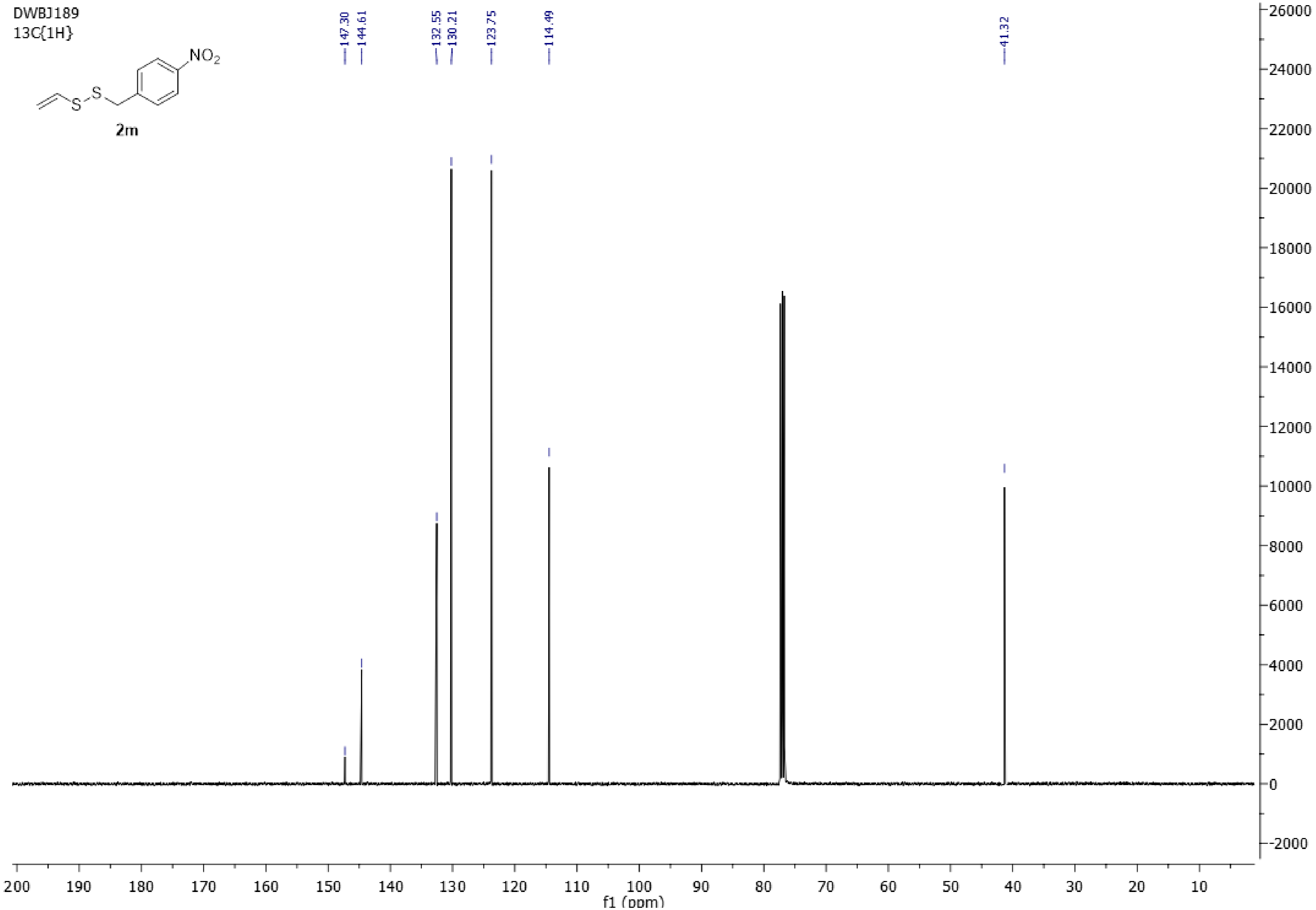
DWBJ189
13C{1H}
NO2
S
S
2m
147.30
144.61
132.55
130.21
123.75
114.49
41.32
26000
24000
22000
20000
18000
16000
14000
12000
10000
8000
6000
4000
2000
0
-2000
200 190 180 170 160 150 140 130 120 110 100 90 80 70 60 50 40 30 20 10
f1 (ppm)

DWBJ193
1H

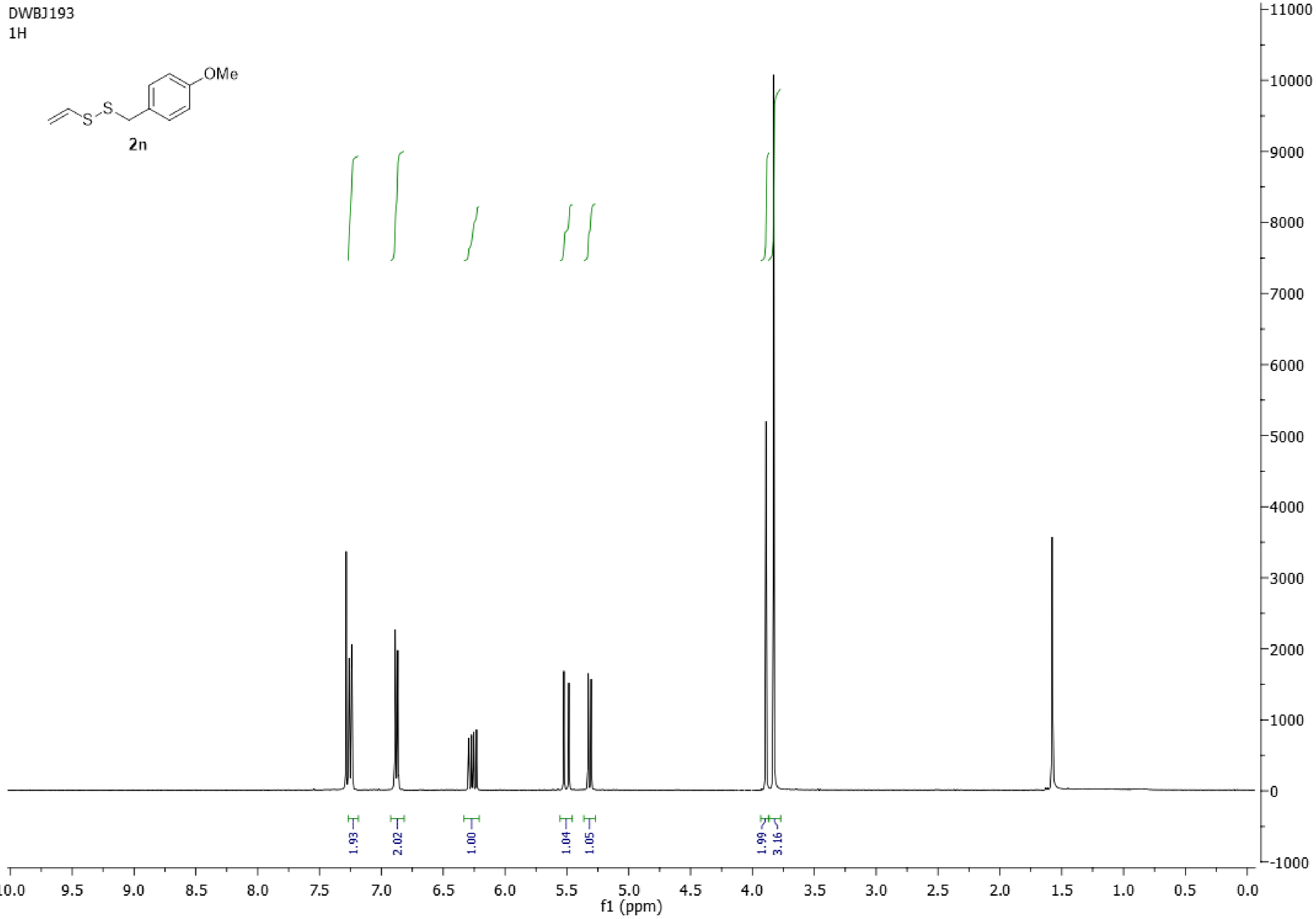
OMe
S
S
2n
11000
10000
9000
8000
7000
6000
5000
4000
3000
2000
1000
0
-1000
1.93
2.02
1.00
1.04
1.05
1.99
3.16
10.0
9.5
9.0
8.5
8.0
7.5
7.0
6.5
6.0
5.5
5.0
4.5
4.0
3.5
3.0
2.5
2.0
1.5
1.0
0.5
0.0
f1 (ppm)

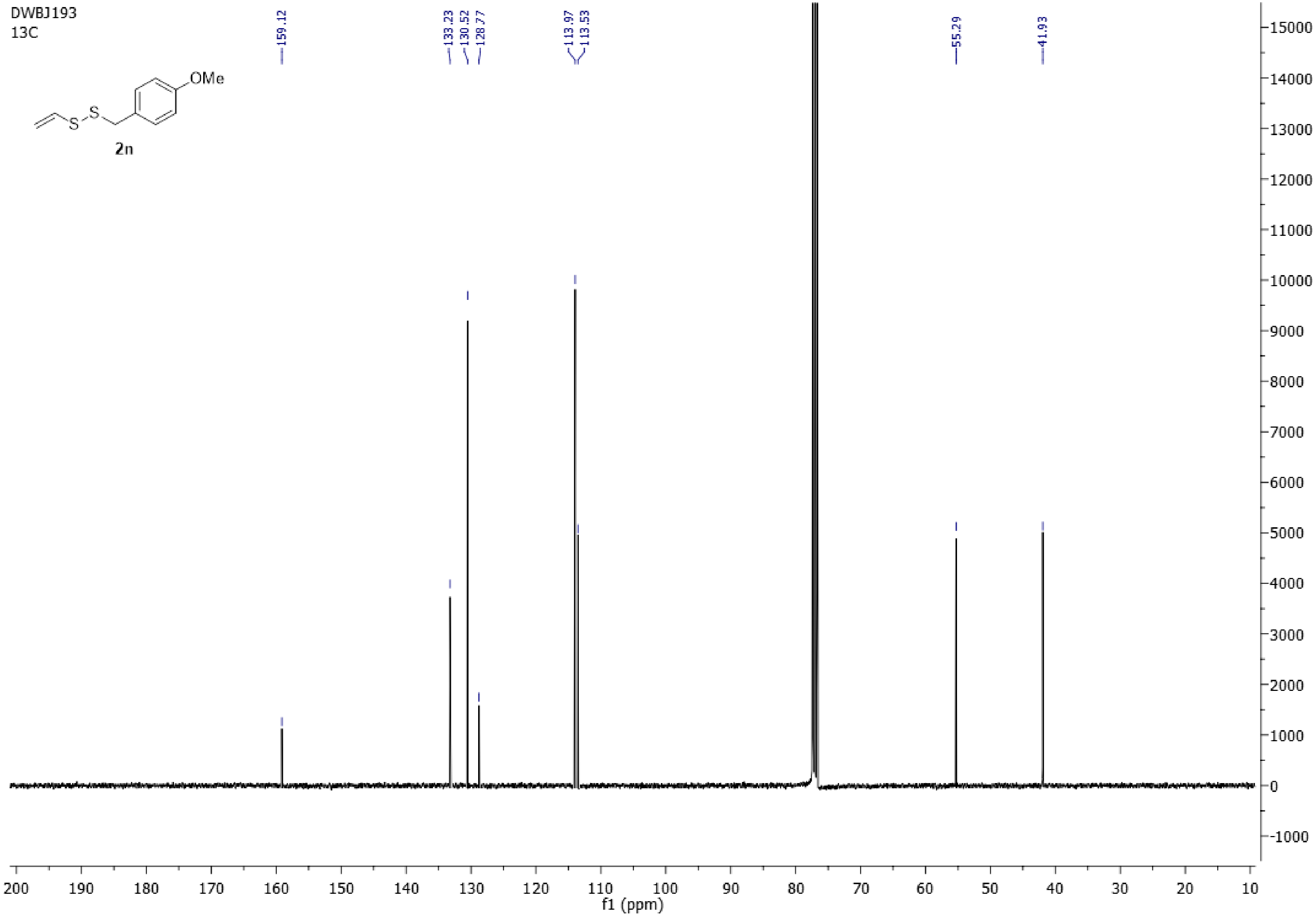

DWBJ193
13C
OMe
S
S
2n
159.12
133.23
130.52
128.77
113.97
113.53
55.29
41.93
f1 (ppm)

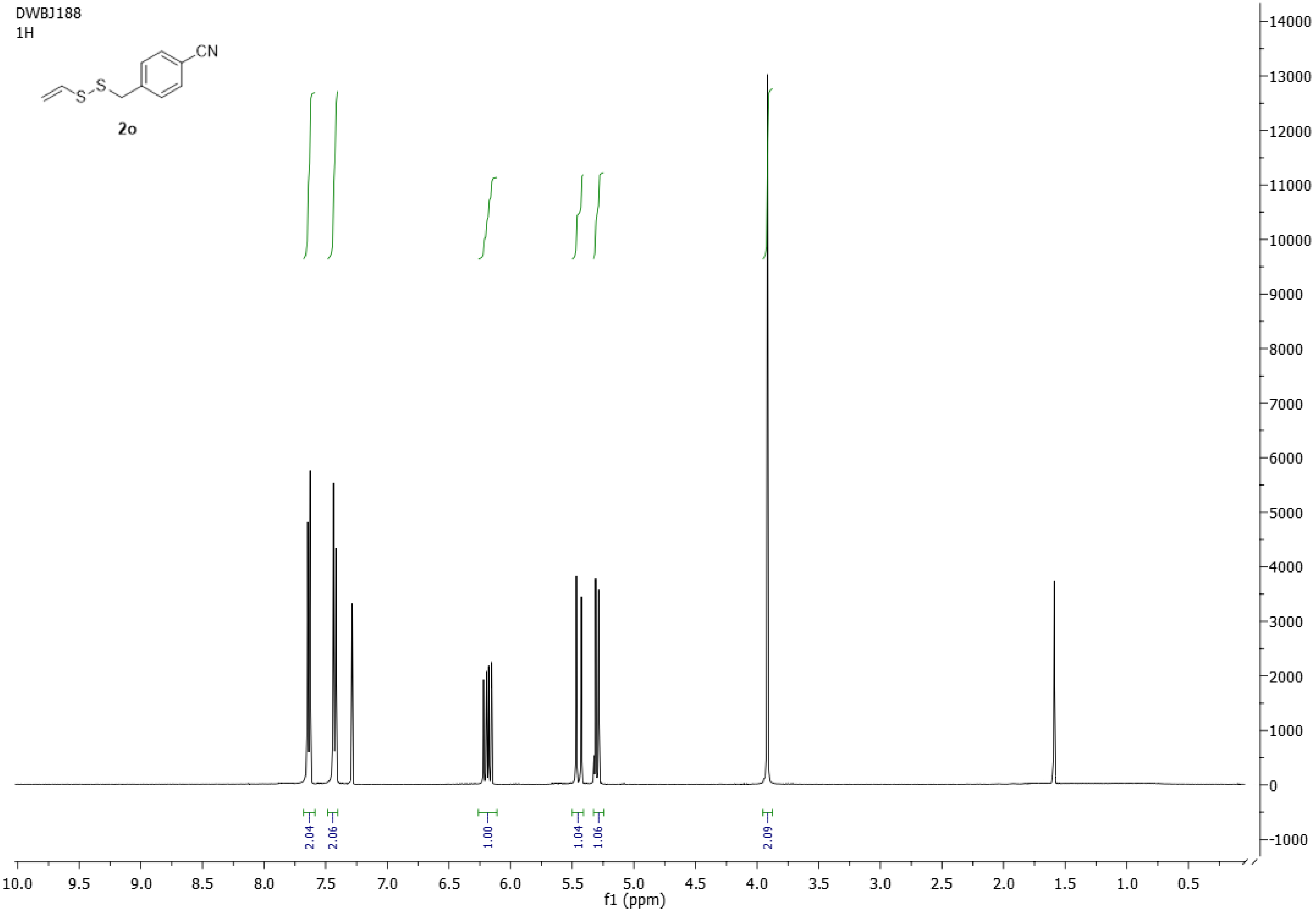

DWBJ188
1H
CN
S
S
2o
2.04
2.06
1.00
1.04
1.06
2.09
f1 (ppm)

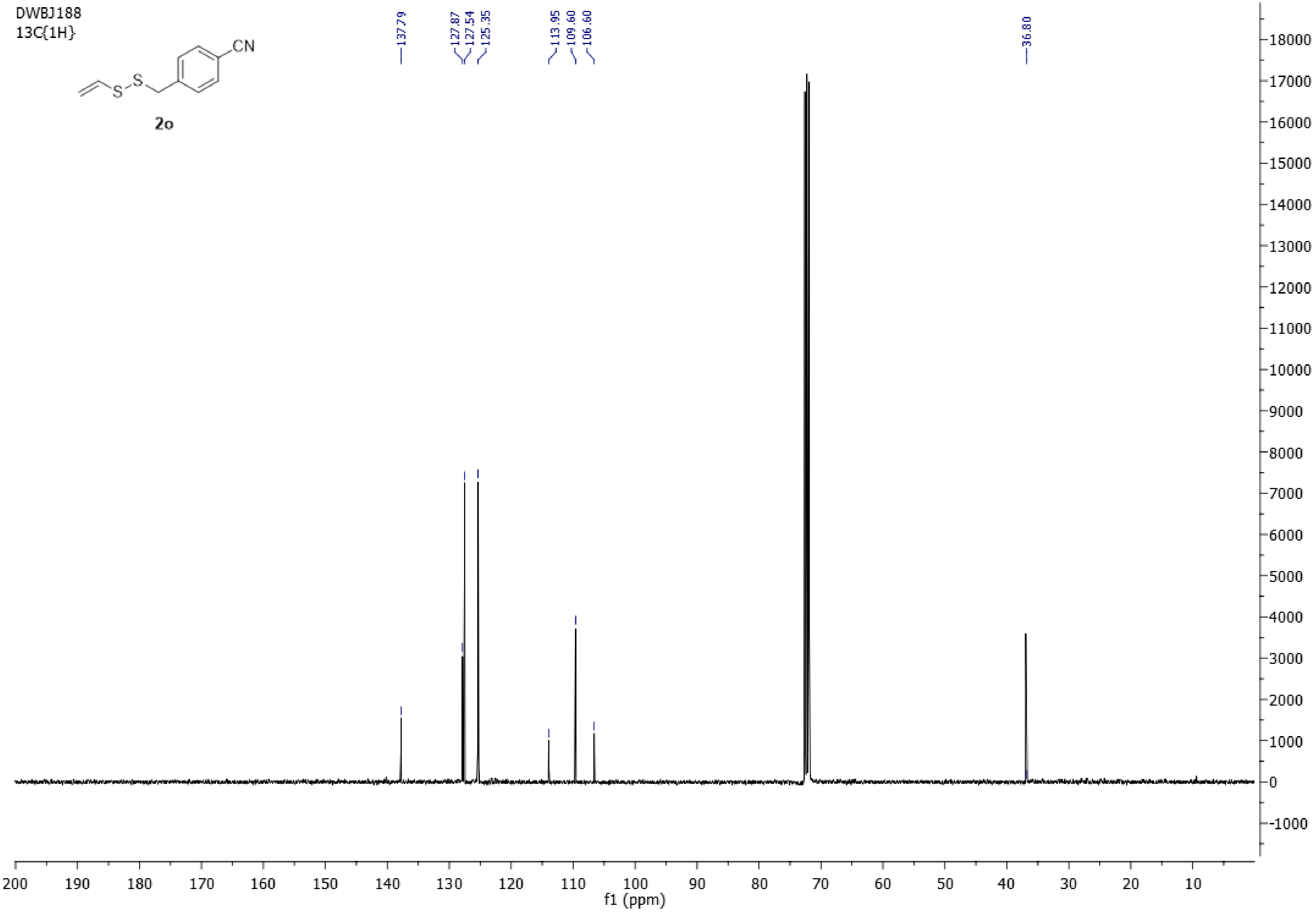
DWBJ188
13C{1H}
CN
S
S
2o
137.79
127.87
127.54
125.35
113.95
109.60
106.60
36.80
f1 (ppm)

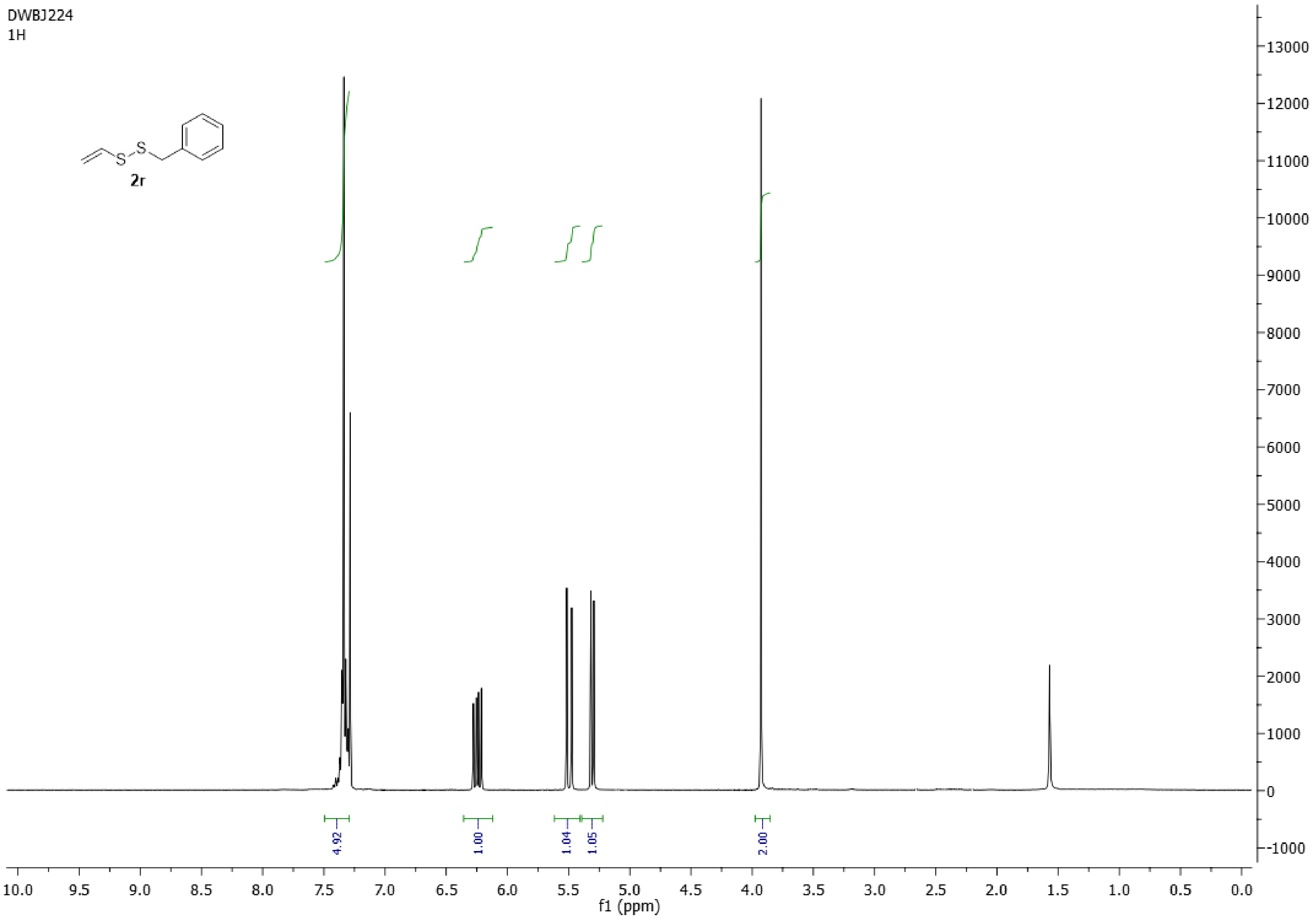

DWBJ224
1H
2r
4.92
1.00
1.04
1.05
2.00
f1 (ppm)

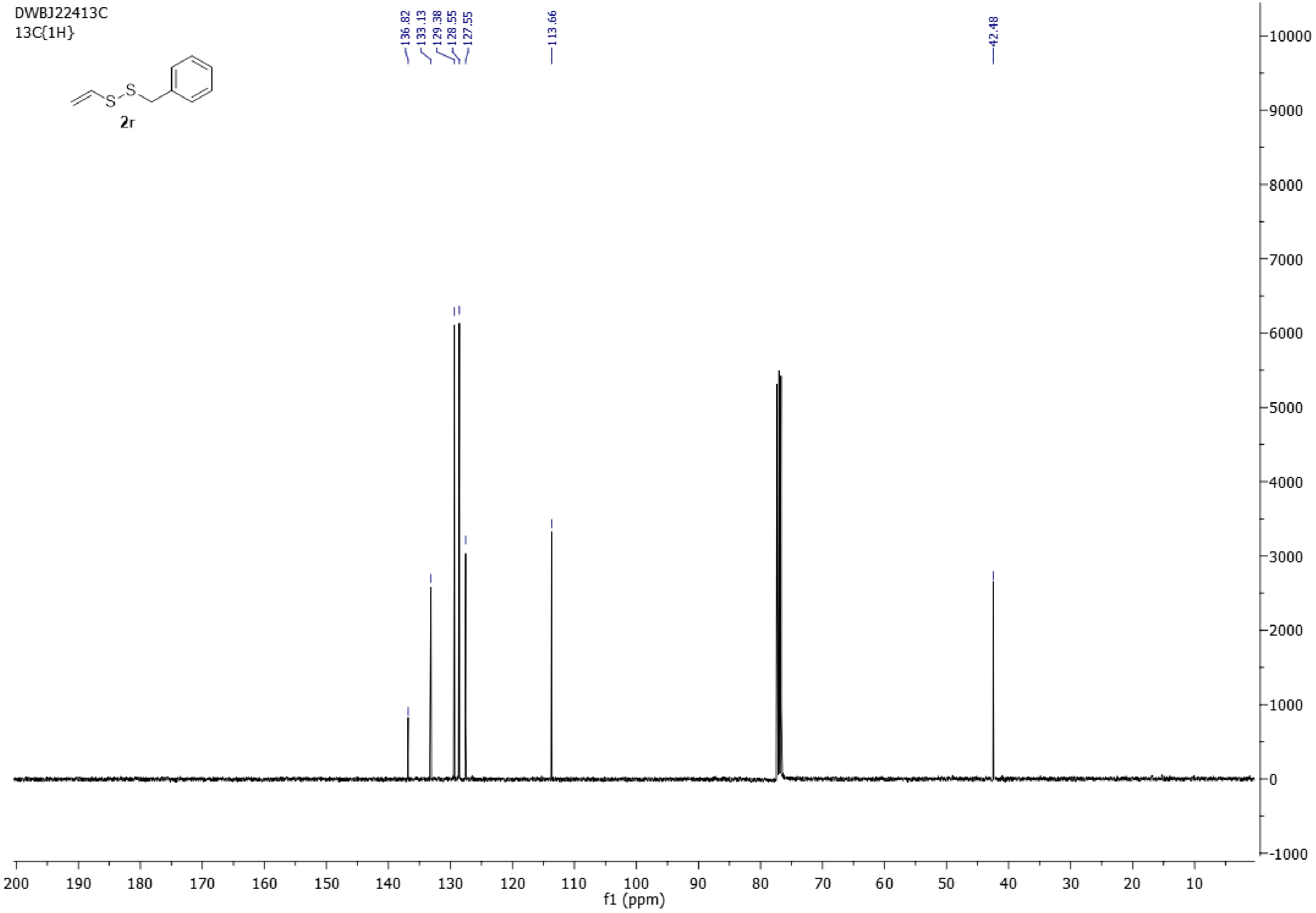

DWBJ22413C
13C{1H}
2r
136.82
133.13
129.38
128.55
127.55
113.66
42.48
10000
9000
8000
7000
6000
5000
4000
3000
2000
1000
0
-1000
200
190
180
170
160
150
140
130
120
110
100
90
80
70
60
50
40
30
20
10
f1 (ppm)

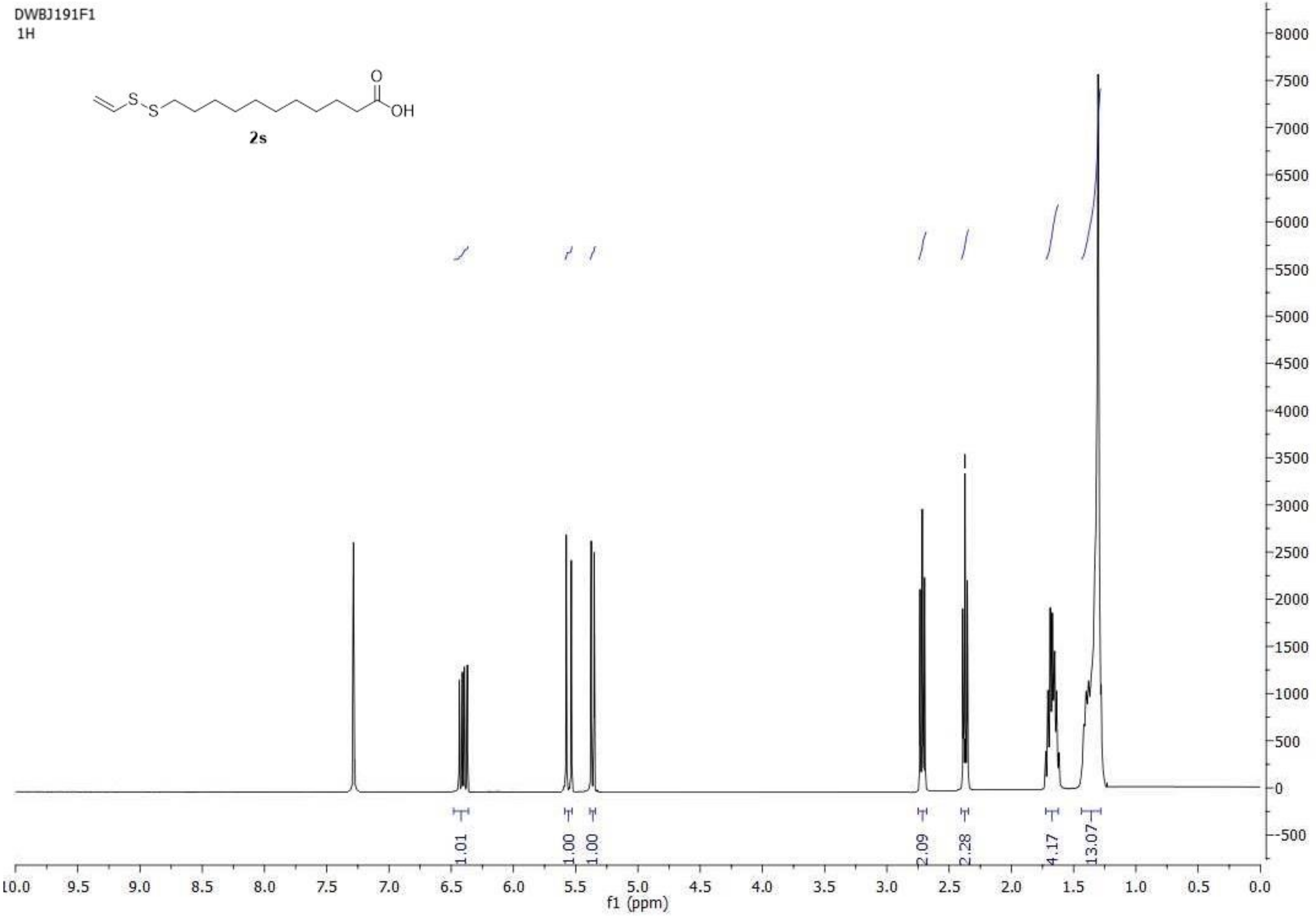
DWBJ191F1
1H
S
S
O
OH
2s
1.01
1.00
1.00
2.09
2.28
4.17
13.07
f1 (ppm)

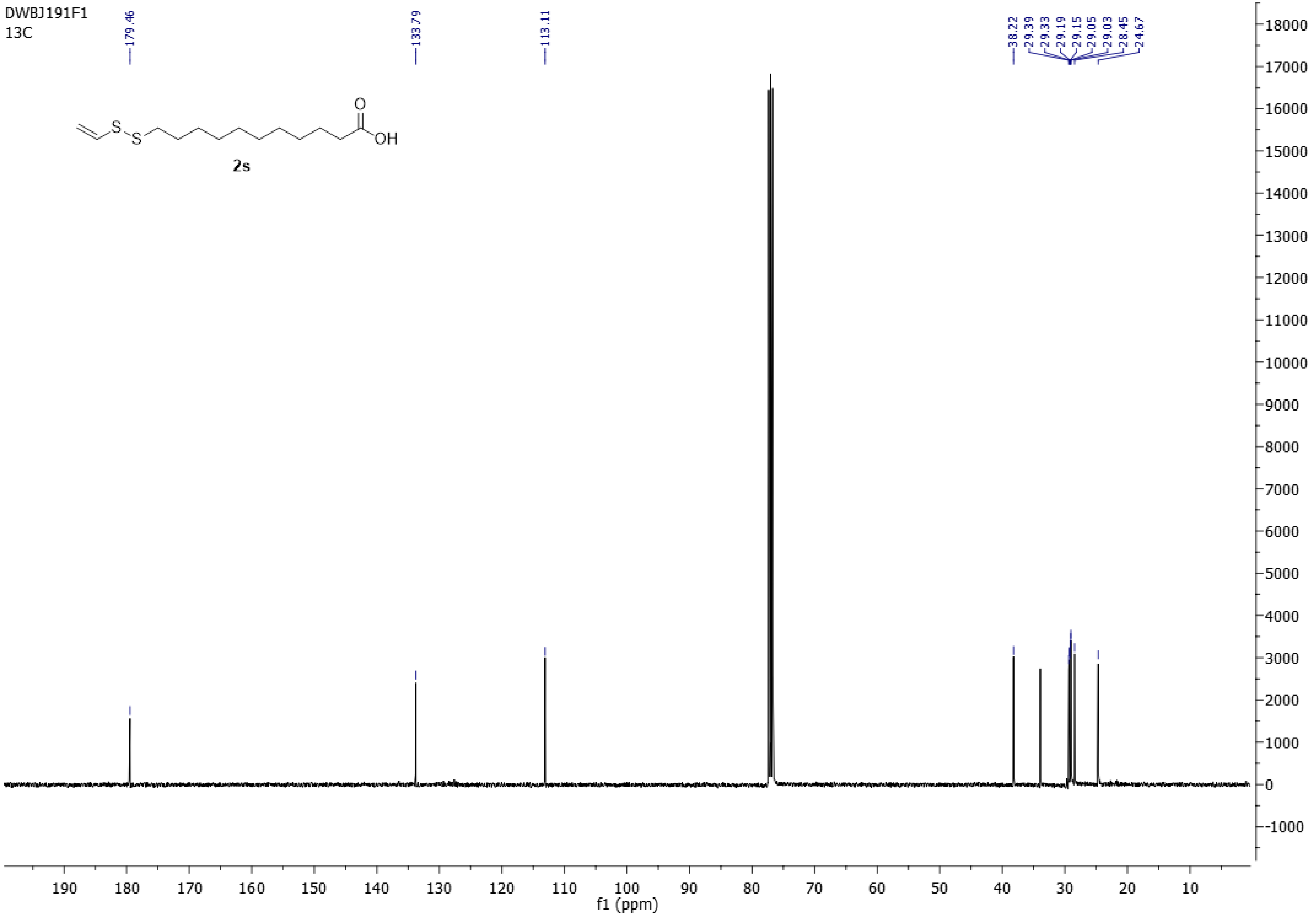
DWBJ191F1
13C
179.46
133.79
113.11
38.22
29.39
29.33
29.19
29.15
29.05
29.03
28.45
24.67
S
S
O
OH
2s
f1 (ppm)

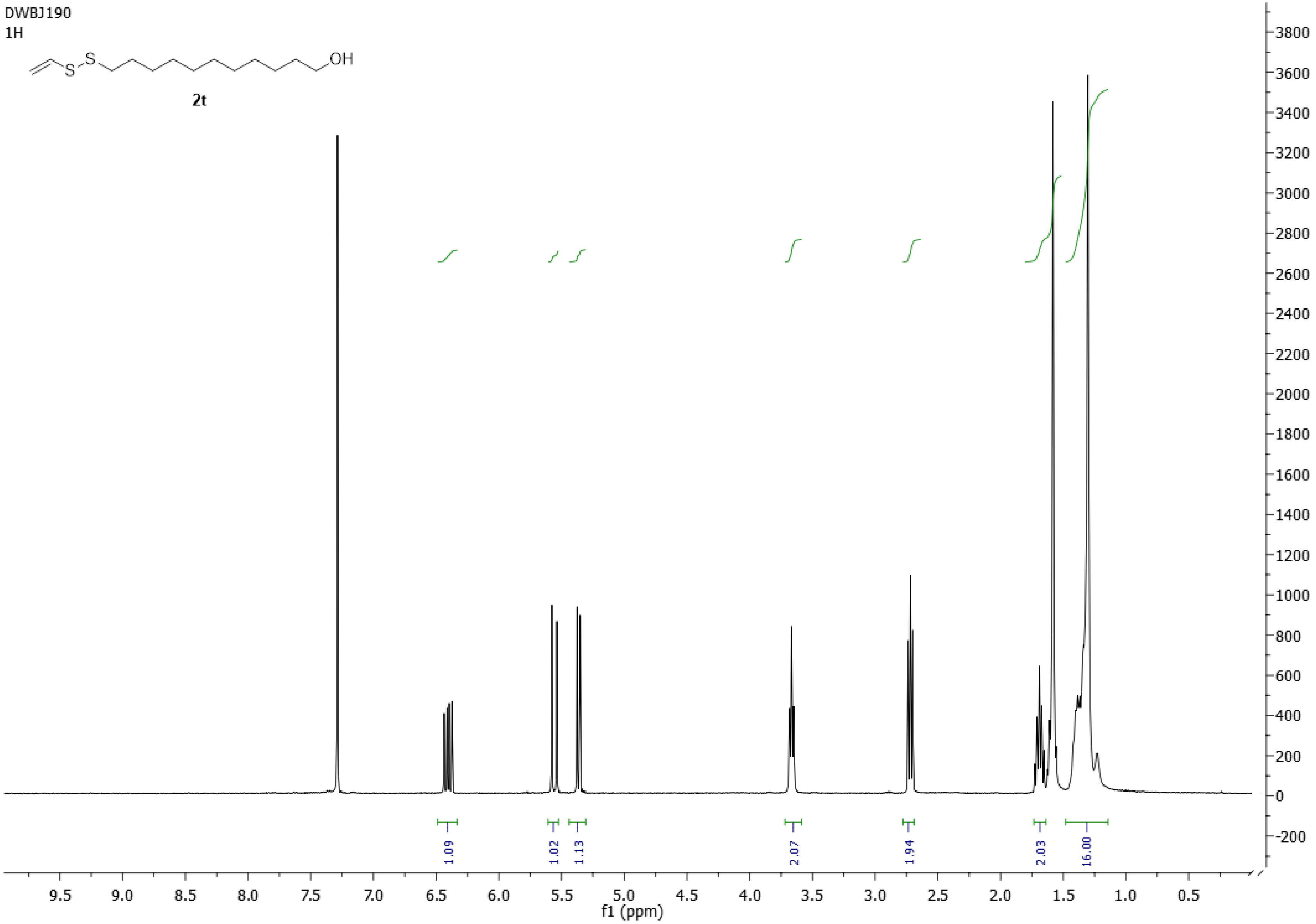
DWBJ190
1H
S
S
OH
2t
1.09
1.02
1.13
2.07
1.94
2.03
16.00
9.5 9.0 8.5 8.0 7.5 7.0 6.5 6.0 5.5 5.0 4.5 4.0 3.5 3.0 2.5 2.0 1.5 1.0 0.5
f1 (ppm)
3800 3600 3400 3200 3000 2800 2600 2400 2200 2000 1800 1600 1400 1200 1000 800 600 400 200 0 -200

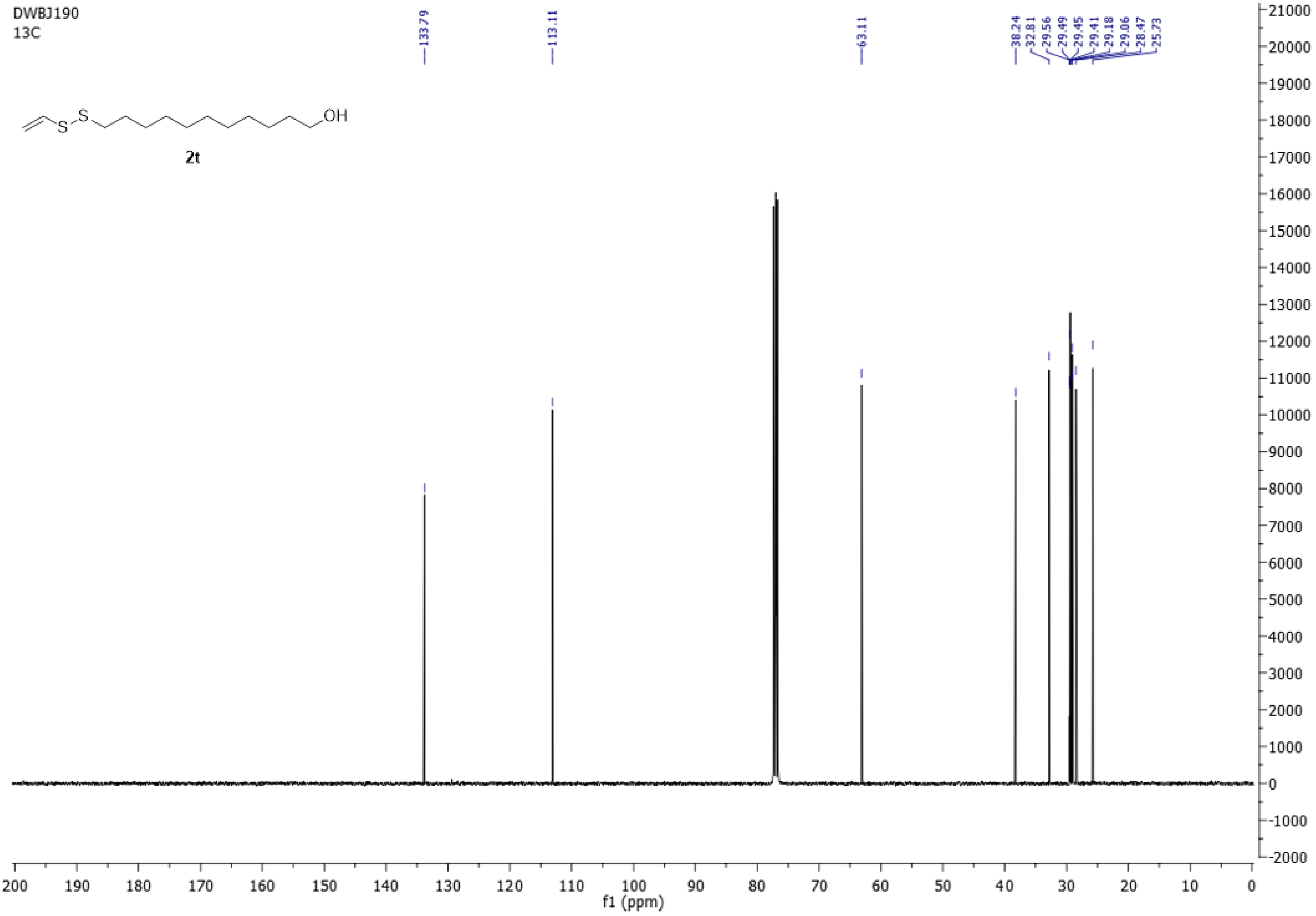
DWBJ190
13C
133.79
113.11
63.11
38.24
32.81
29.56
29.49
29.45
29.41
29.18
29.06
28.47
25.73
OH
S
S
2t
f1 (ppm)

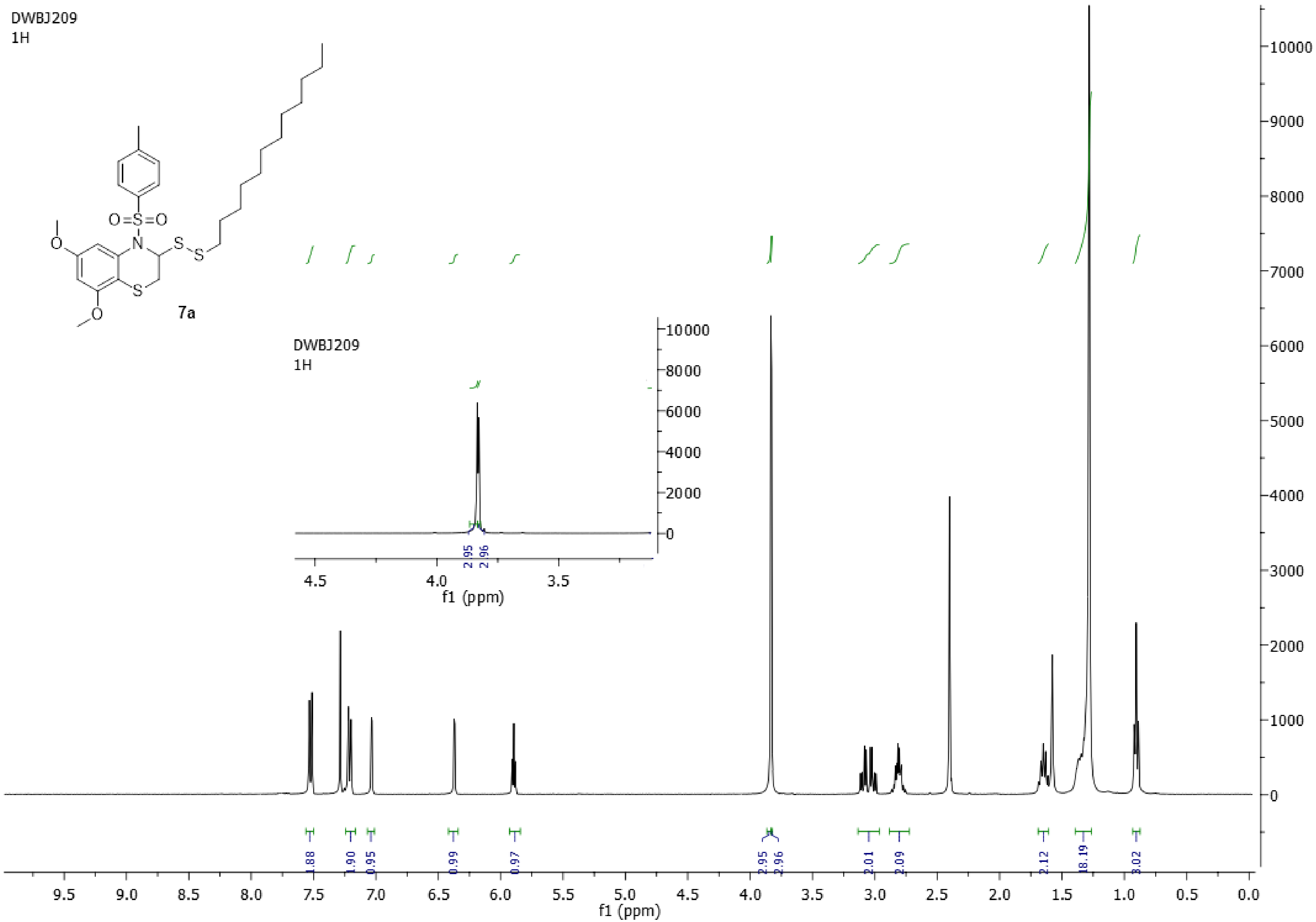
DWBJ209
1H
7a
f1 (ppm)

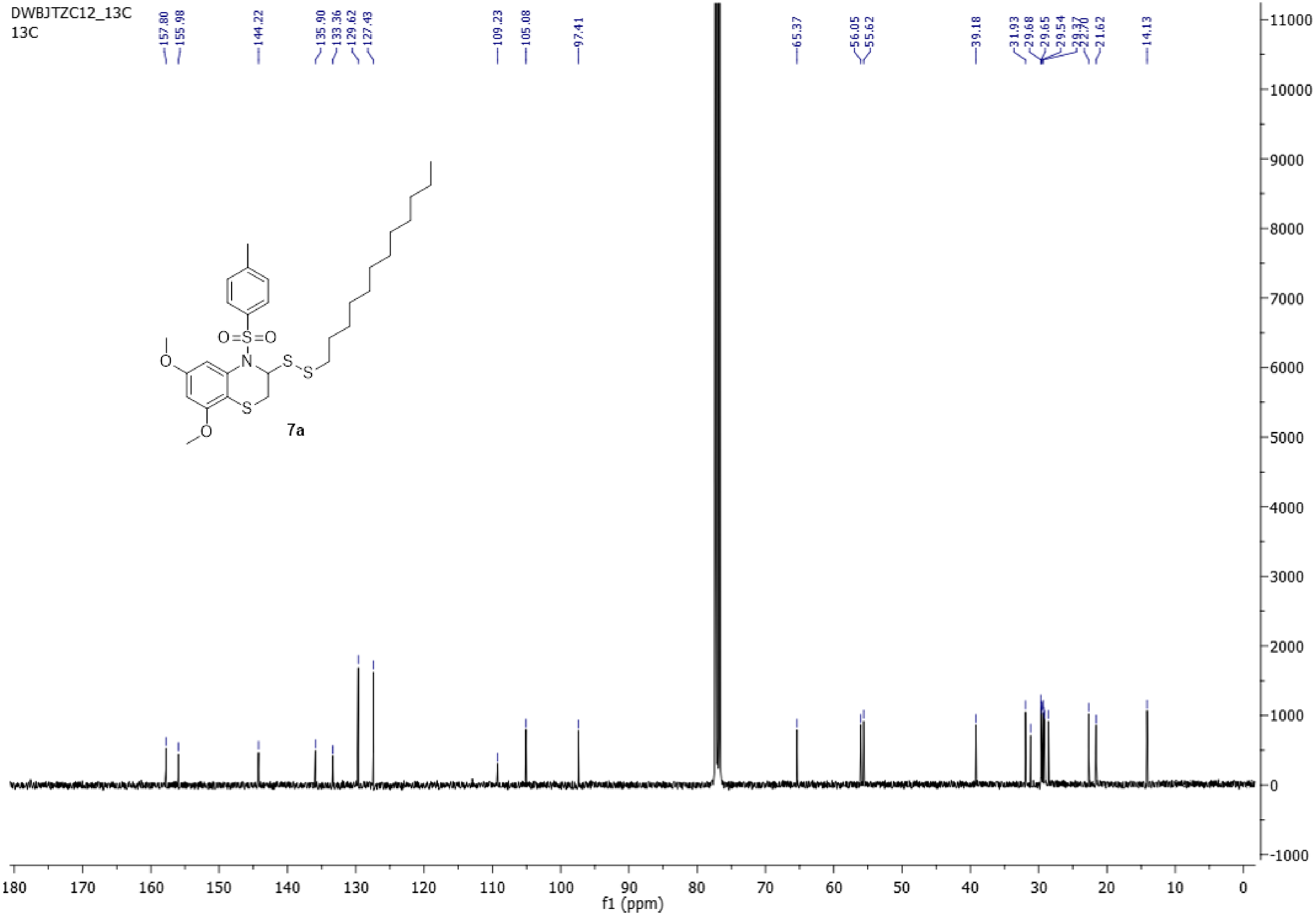
DWBJTZC12_13C
13C
157.80
155.98
144.22
135.90
133.36
129.62
127.43
109.23
105.08
97.41
65.37
56.05
55.62
39.18
31.93
29.68
29.65
29.54
29.37
22.70
21.62
14.13
O=S=O
N
S
S
S
O
O
7a
f1 (ppm)

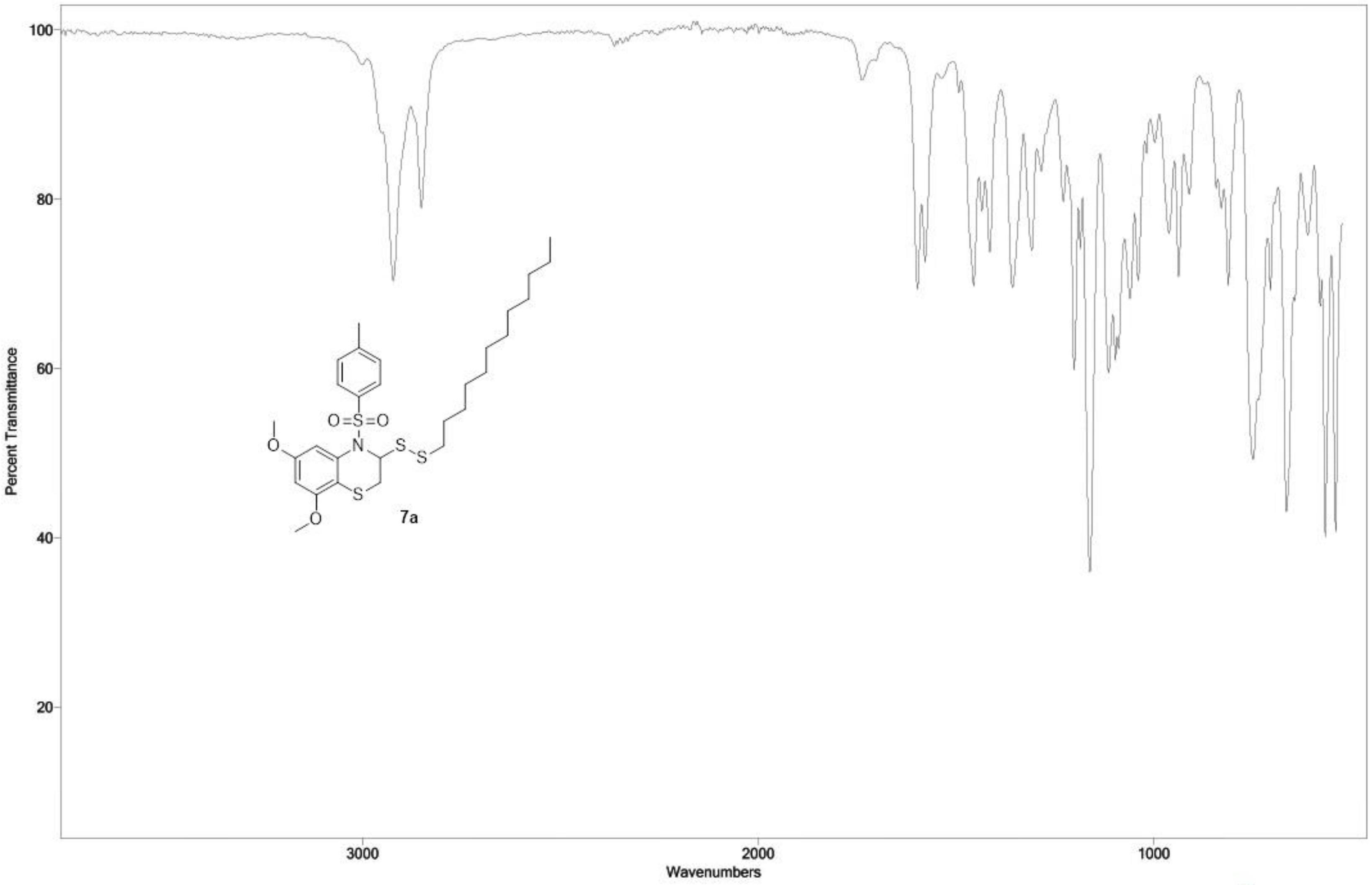

printed on 06/13/2020 14:22:25

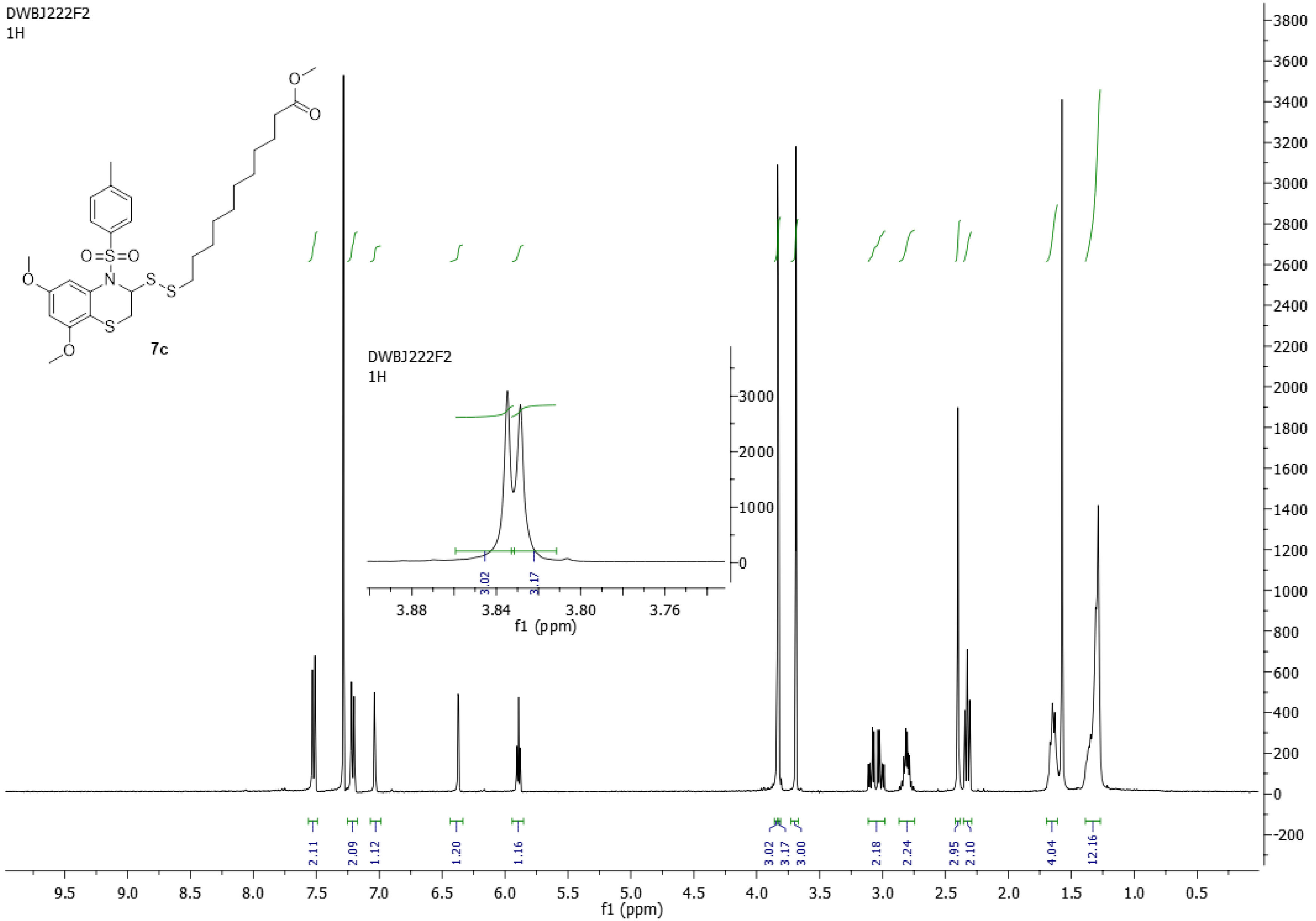

DWBJ222F2
1H
7c
DWBJ222F2
1H
3.02
3.17
3.88
3.84
3.80
3.76
f1 (ppm)
2.11
2.09
1.12
1.20
1.16
3.02
3.17
3.00
2.18
2.24
2.95
2.10
4.04
12.16
f1 (ppm)

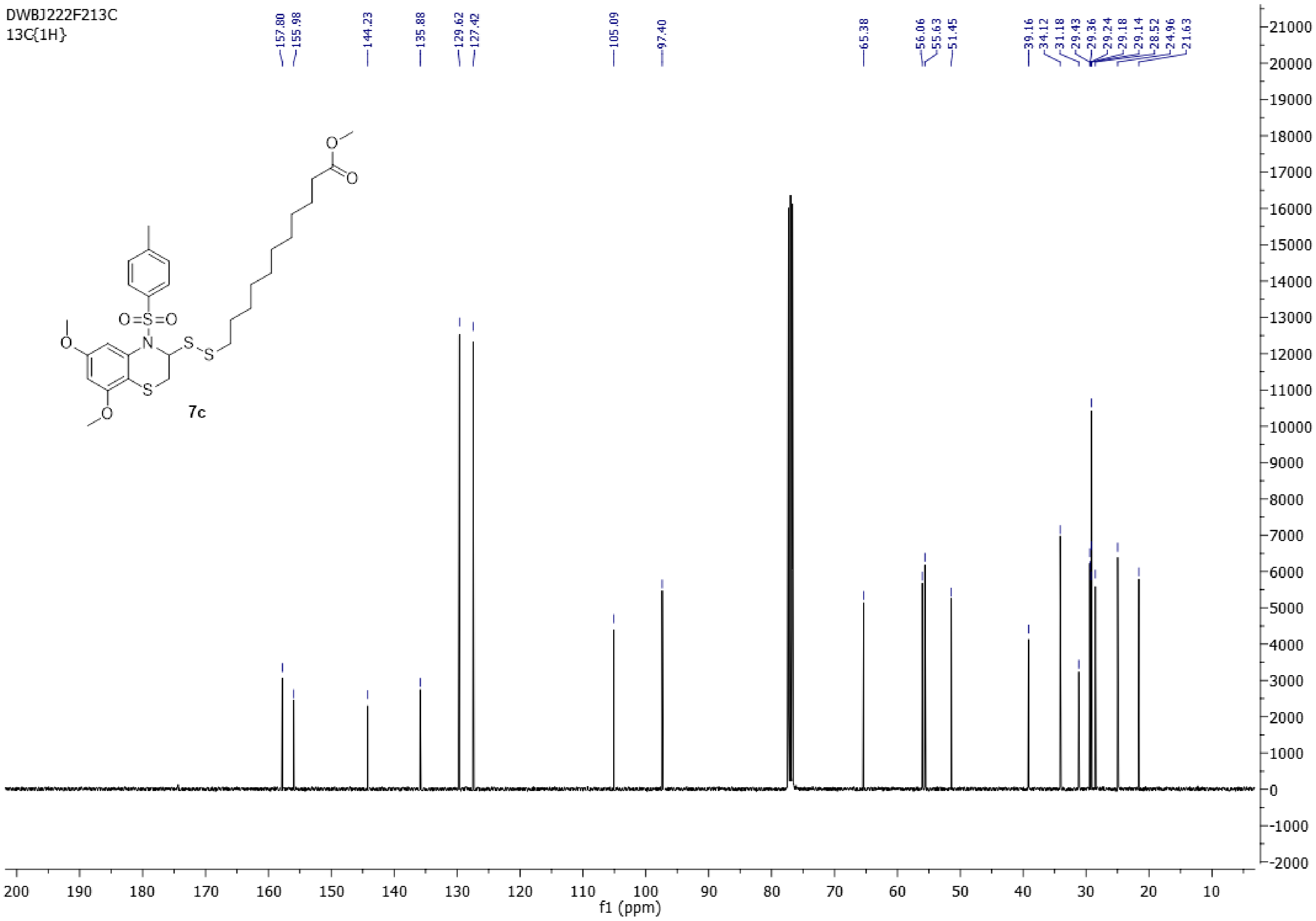
DWBJ222F213C
13C{1H}
157.80
155.98
144.23
135.88
129.62
127.42
105.09
97.40
65.38
56.06
55.63
51.45
39.16
34.12
31.18
29.43
29.36
29.24
29.18
29.14
28.52
24.96
21.63
7c
f1 (ppm)

Wt Cze 09 10-53-50 2020 tia ester.CSV: Column 1

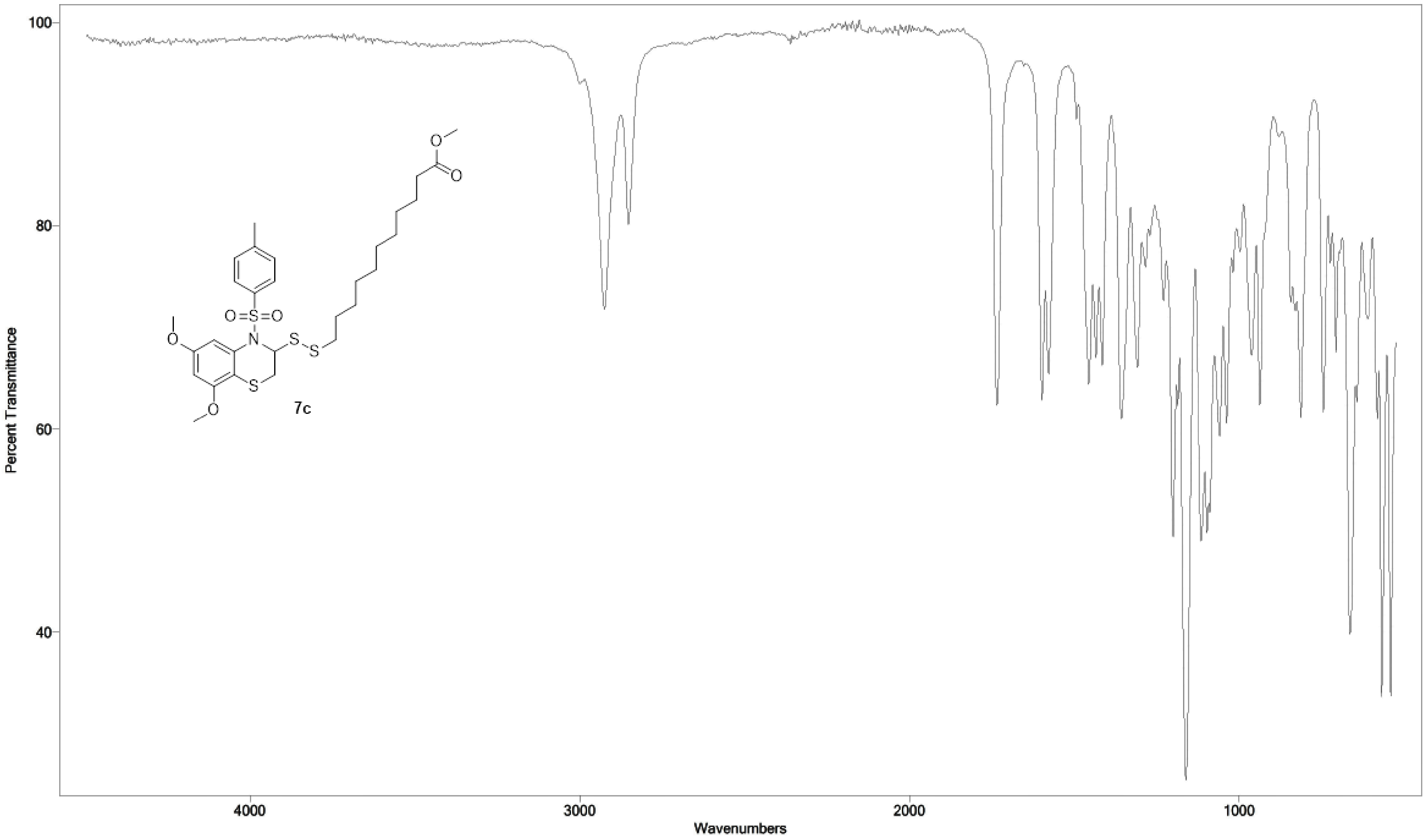

printed on 06/13/2020 14:39:34

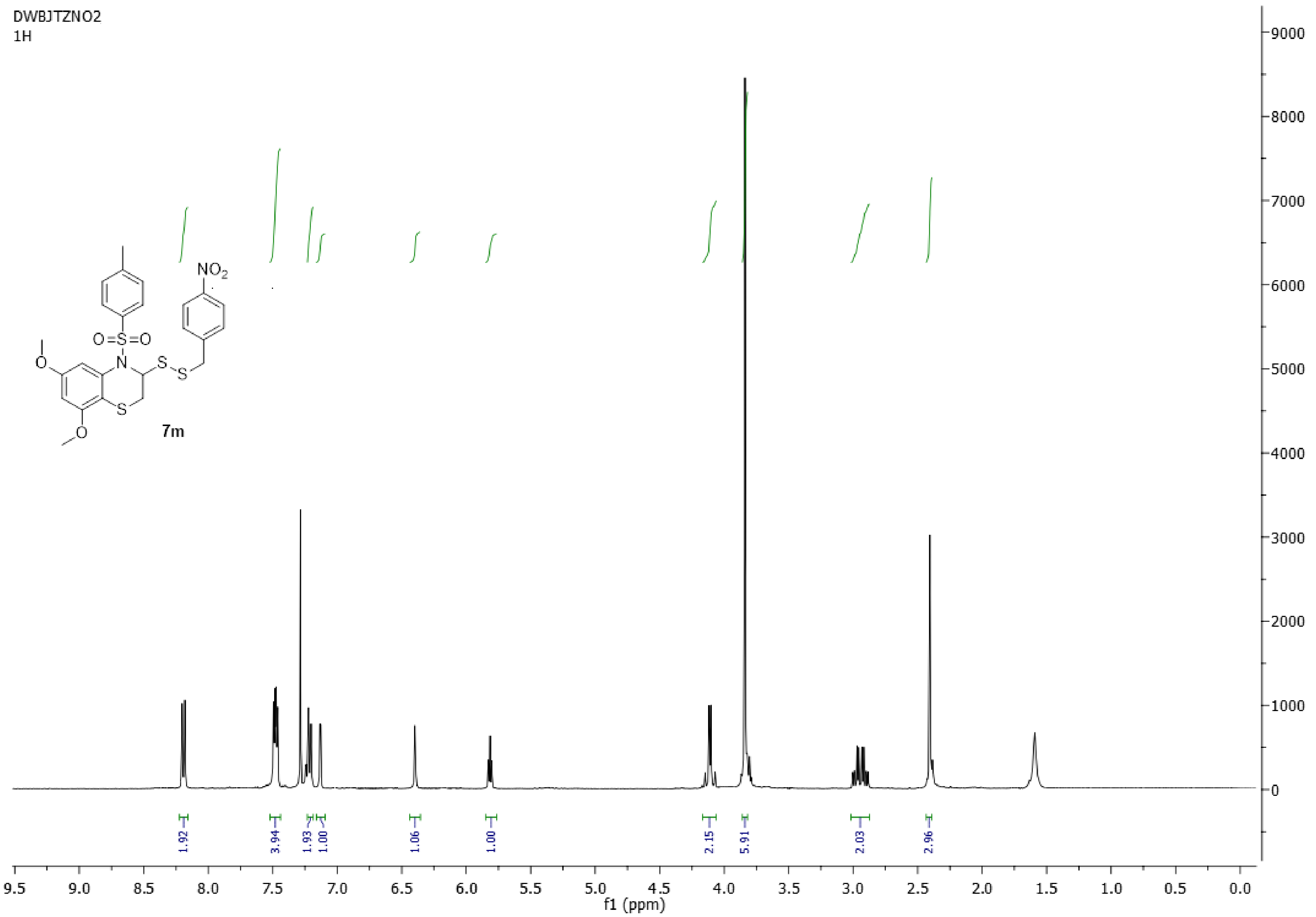
DWBJTZNO2
1H
NO2
O=S=O
N
S
S
S
O
O
1.92
3.94
1.93
1.00
1.06
1.00
2.15
5.91
2.03
2.96
f1 (ppm)

7m

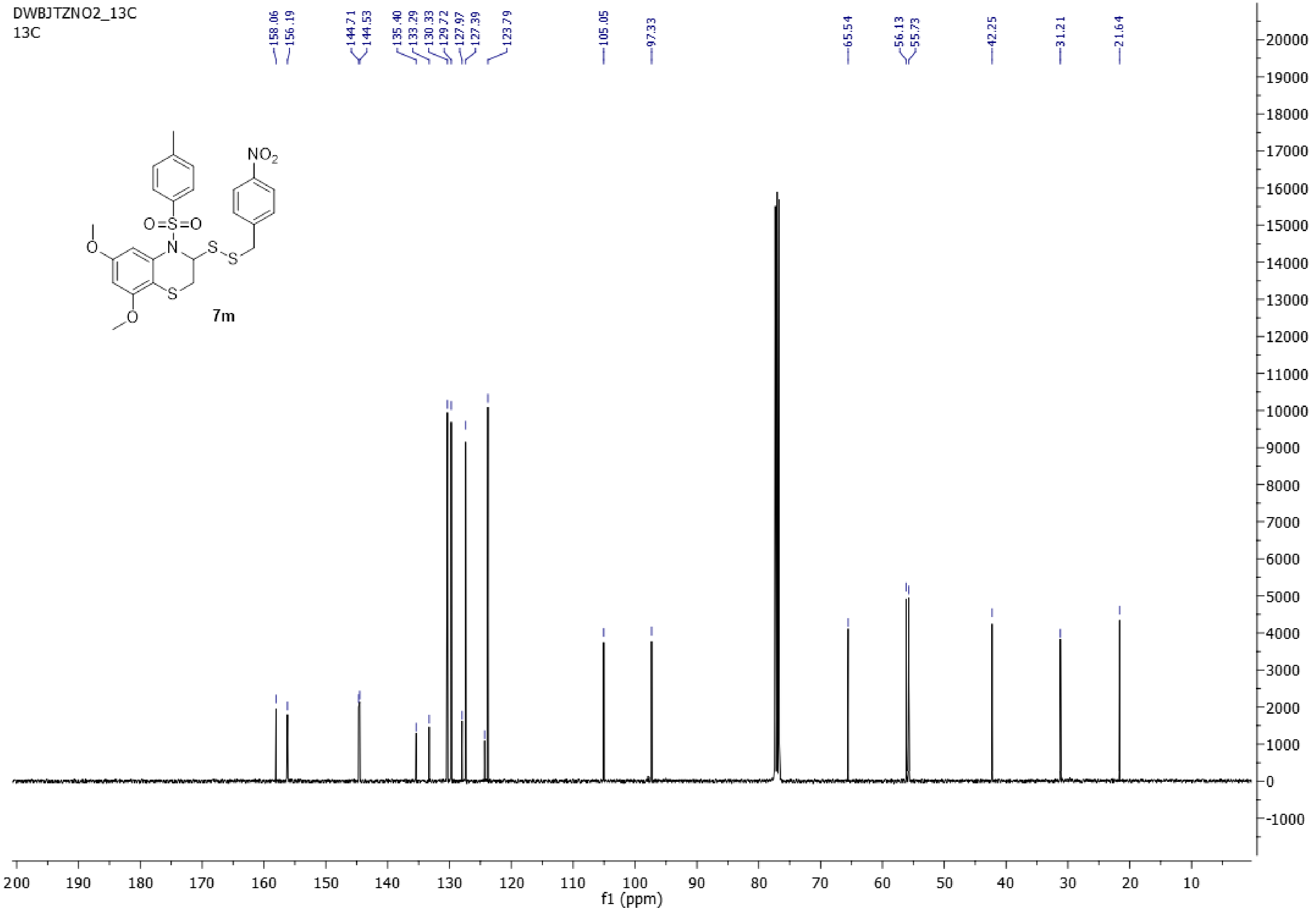
DWBJTZNO2_13C
13C
158.06
156.19
144.71
144.53
135.40
133.29
130.33
129.72
127.97
127.39
123.79
105.05
97.33
65.54
56.13
55.73
42.25
31.21
21.64
NO2
O=S=O
N
S
S
O
S
O
7m
f1 (ppm)

Wt Cze 09 10-49-55 2020 tiaBNNO2.CSV: Column 1

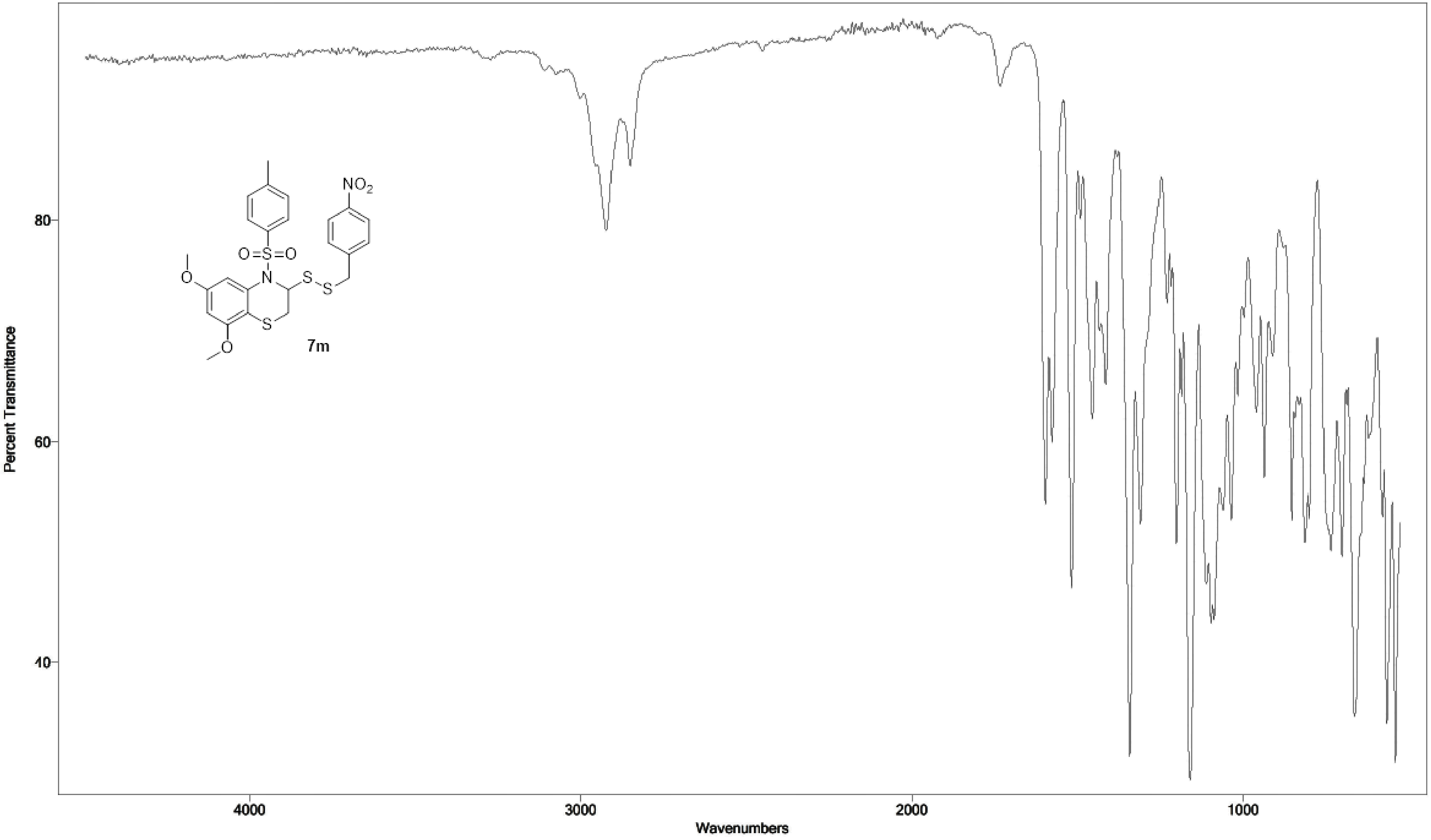

printed on 06/13/2020 14:36:43

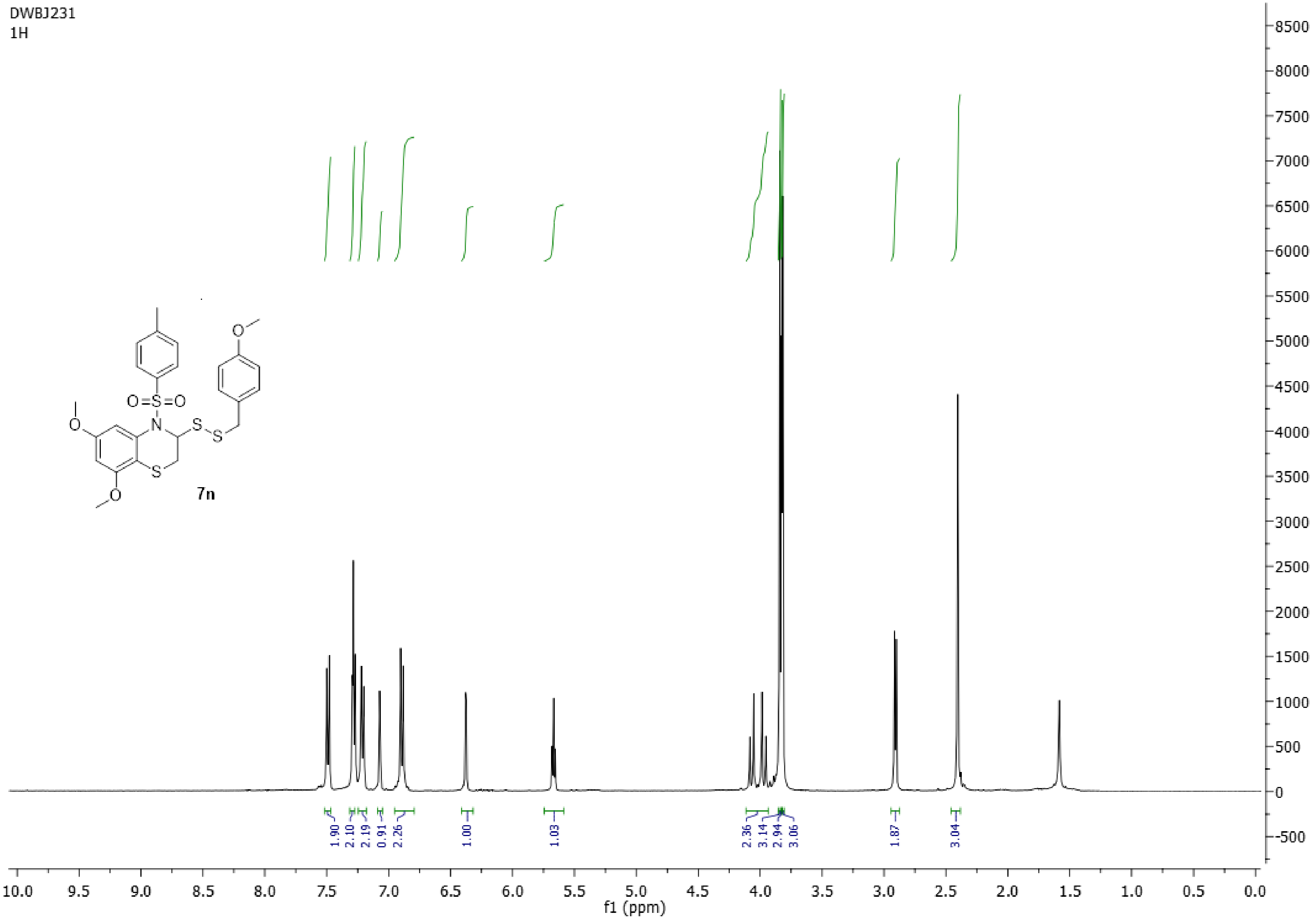
DWBJ231
1H
7n
f1 (ppm)

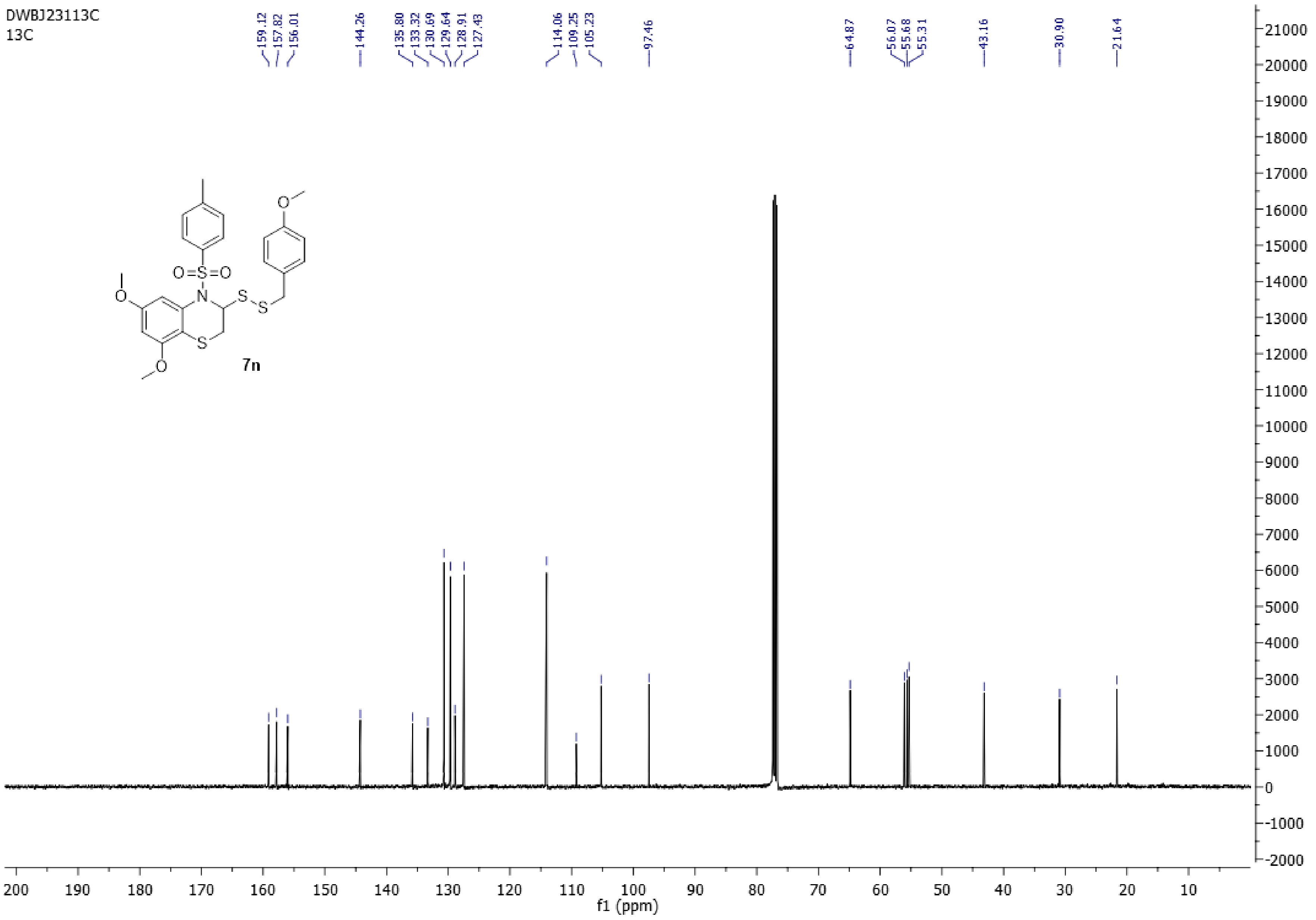
DWBJ23113C
13C
159.12
157.82
156.01
144.26
135.80
133.32
130.69
129.64
128.91
127.43
114.06
109.25
105.23
97.46
64.87
56.07
55.68
55.31
43.16
30.90
21.64
7n
f1 (ppm)

Wt Cze 09 10-44-12 2020 tiaBNOMe.CSV: Column 1

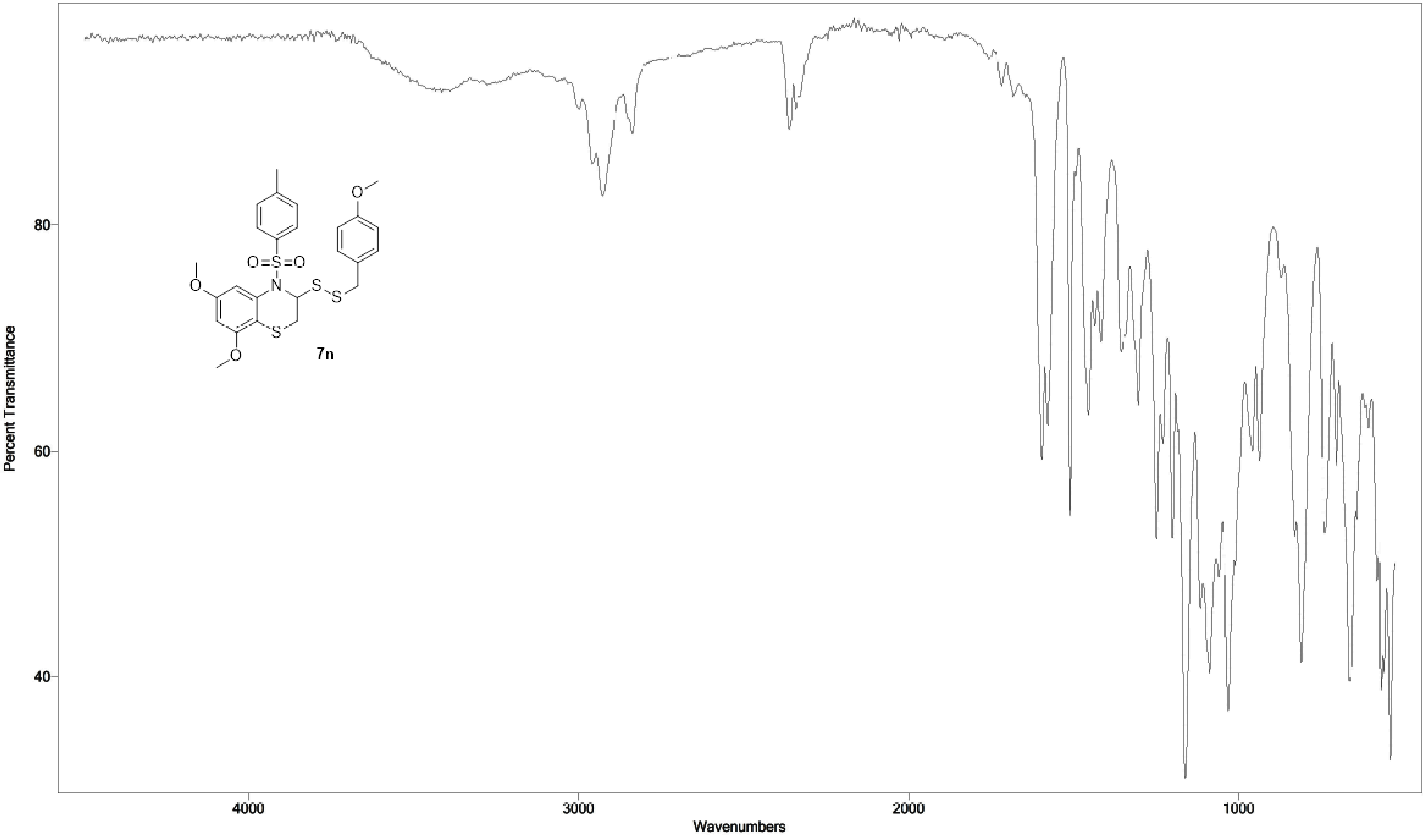

printed on 06/13/2020 14:32:38

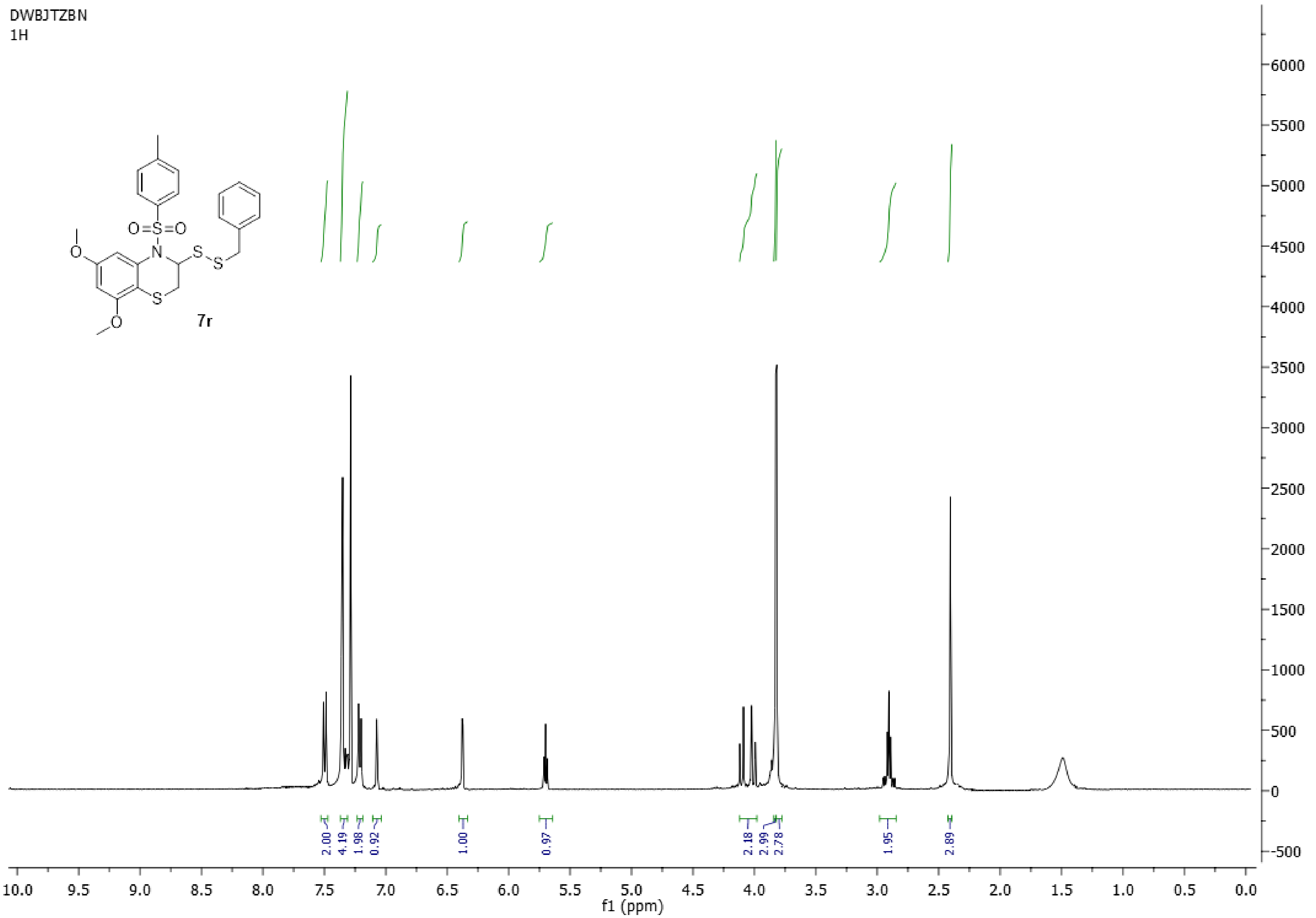
DWBJTZBN
1H
7r
2.00
4.19
1.98
0.92
1.00
0.97
2.18
2.99
2.78
1.95
2.89
f1 (ppm)

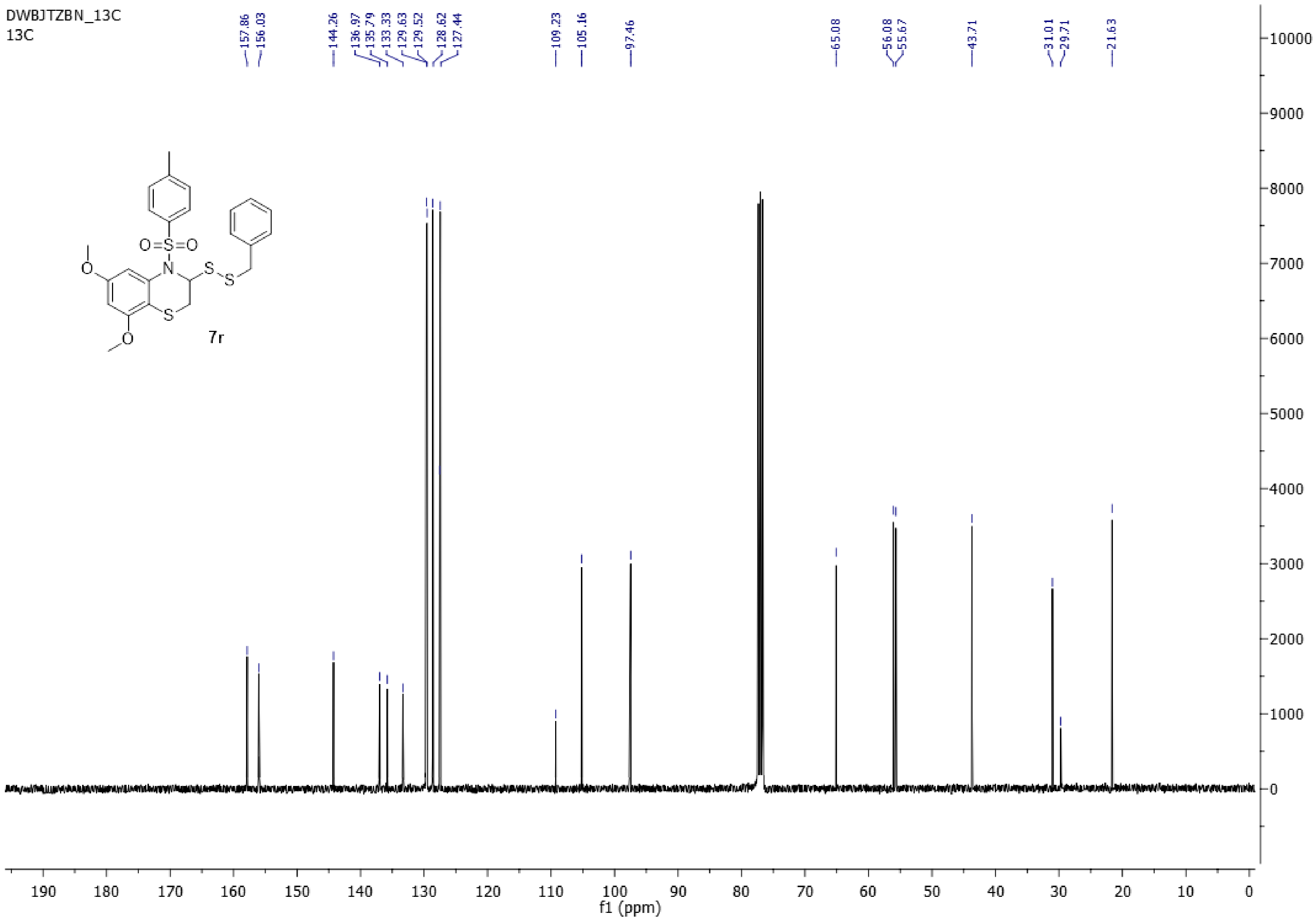
DWBJTZBN_13C
13C
157.86
156.03
144.26
136.97
135.79
133.33
129.63
129.52
128.62
127.44
109.23
105.16
97.46
65.08
56.08
55.67
43.71
31.01
29.71
21.63
7r
f1 (ppm)

Wt Cze 09 10-39-41 2020 tiaBN.CSV: Column 1

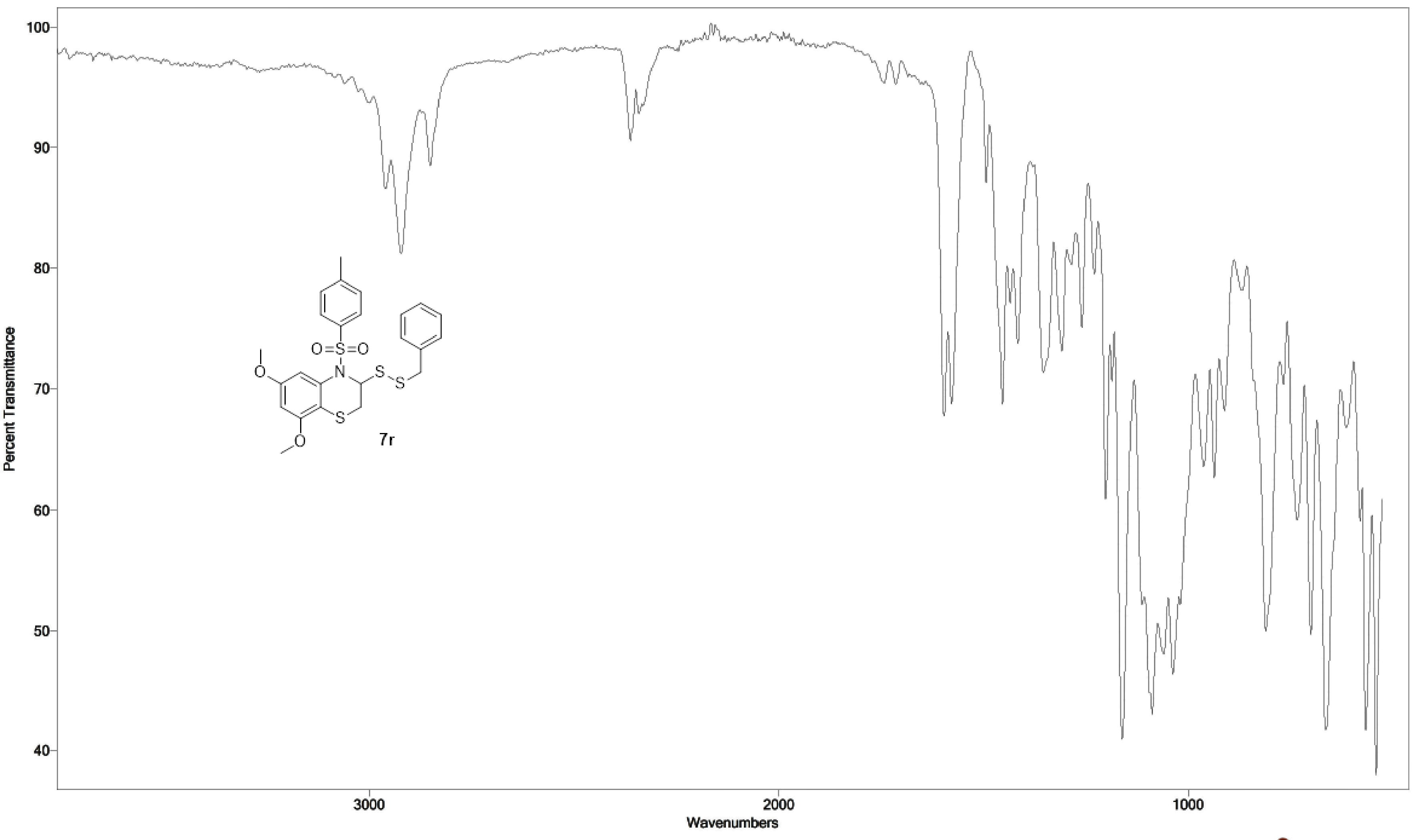

printed on 06/13/2020 14:28:29
